# SCS 5th Annual Conference. São Paulo, Brazil, 2022

**DOI:** 10.3390/sports11050095

**Published:** 2023-04-28

**Authors:** Pedro E. Alcaraz, Elena Marín-Cascales, Anthony J. Blazevich, Lucas A. Pereira, Valter P. Mercer, Túlio B. M. A. Moura, Victor Fernandes, Tomás T. Freitas, Irineu Loturco

**Affiliations:** 1 UCAM Research Center for High Performance Sport, UCAM Universidad Católica de Murcia, 30107 Murcia, Spain; palcaraz@ucam.edu (P.E.A.); tfreitas@ucam.edu (T.T.F.); 2 Facultad de Deporte, UCAM Universidad Católica de Murcia, 30107 Murcia, Spain; 3 Strength & Conditioning Society, 30008 Murcia, Spain; a.blazevich@ecu.edu.au; 4 Centre for Exercise and Sports Science Research, School of Medical and Health Sciences, Edith Cowan University, Joondalup 6027, Australia; 5 NAR-Nucleus of High Performance in Sport, São Paulo 04753-060, Brazil; lucasa_pereira@outlook.com (L.A.P.); valterpreis@gmail.com (V.P.M.); tuliobernardo@gmail.com (T.B.M.A.M.); victorfernandescoach@gmail.com (V.F.); irineu.loturco@terra.com.br (I.L.); 6 Department of Human Movement Sciences, Federal University of São Paulo, São Paulo 11015-020, Brazil; 7 Faculty of Life Sciences and Education, University of South Wales, Pontypridd, Wales CF037 1DL, UK

**Keywords:** congress, performance, exercise, health, training, injury

## Abstract

On behalf of the Strength and Conditioning Society (SCS) and the Nucleus of High Performance in Sport (NAR), we are pleased to present the abstracts of the SCS 5th Annual Conference, which, for the first time, took place outside of Europe. The event was held at NAR’s state-of-the-art facilities in São Paulo, Brazil, on 3–5 November 2022, and comprised several invited sessions from international and national speakers on a variety of topics related to strength and conditioning practices and their application to health, injury prevention and sports performance. These included strength training in high-performance sports and older adults, sleep and recovery in elite athletes, performance optimization of the female athlete, high-intensity interval training, velocity-based resistance training, and running and cycling biomechanics, among others. The Conference also included different practical workshops conducted by renowned academics and practitioners on post-competition recovery strategies, plyometric training, hamstring strain injuries in soccer, and resisted sprint training. Finally, the event disseminated up-to-date strength and conditioning research by providing practitioners and researchers with the opportunity to present their most recent findings. In this regard, all abstracts of the communications presented at the SCS 5th Annual Conference can be found in this Conference Report.

## 1. Introduction

The 5th Annual Conference of the Strength and Conditioning Society (SCS) was SCS’ first international event organized outside of Europe. In accordance with the society’s vision and mission to disseminate high-quality evidence of the performance and health benefits of strength and conditioning practices worldwide, the 2022 Conference took place in South America, on 3–5 November. The event was organized in collaboration with the Nucleus of High Performance in Sport (NAR), an internationally renowned training, assessment and research center located in São Paulo (Brazil), that provides support to hundreds of world-class athletes from more than 70 sport disciplines. The SCS 5th Annual Conference, held at NAR’s state-of-the-art facilities, brought together more than 250 delegates from different areas of expertise (i.e., sports science, sports physiotherapy, sports nutrition, and sports medicine, among others) which provided ample opportunities to exchange and discuss the latest evidence on strength and conditioning practices from multiple perspectives. In a stimulating social and professional environment, practitioners and academics from different countries had the possibility of attending several thought-provoking invited sessions from international and national speakers on a variety of topics related to strength and conditioning and its application to health, injury prevention and sports performance. These topics included strength training in high-performance sports and older adults, sleep and psychobiological responses in athletic populations, performance optimization of women in sport, complex-contrast and circuit training in team sports, velocity-based resistance training, high-intensity interval training, muscle mass assessment in elite soccer and running and cycling biomechanical aspects related to performance and injury. Furthermore, the Conference also offered multiple practical workshops conducted by renowned academics and practitioners (i.e., Olympic and soccer and rugby professional strength and conditioning coaches) on post-competition recovery strategies, plyometric training, hamstring strain injuries in soccer, and resisted sprint training. As in previous years, the event fostered the dissemination of up-to-date strength and conditioning research by providing practitioners and researchers with the opportunity to present and discuss their latest findings, that can be found in the abstracts that compose this Conference Report. Finally, the SCS, in collaboration with NAR, recognized professional and academic excellence in the field of strength and conditioning and presented the “Strength and Conditioning Coach of the Year Award”, the “Emerging Strength and Conditioning Coach of the Year Award” and the “Strength and Conditioning Coach Career Achievement Award” to outstanding coaches, and the “Young Investigator Award” and the “Applied Science Award” to remarkable researchers.

## 2. Conference Abstract

### 2.1. General and Specific Vertical Jumping Ability in Junior and Professional Brazilian Elite Male Volleyball Players

**Cesar Abad, Jerusa Lara, Marcos Lopes, Luiz Lins, Jaime Lansini, Leandro Silva, José Silva, Rodolfo Ferreira, Anderson Rodrigues and Fabiano Teixeira**

Reference Center of Sport Science of Service of Social Industry (CRCE-SESI); cesar.abad@sesisp.org.br (C.A.); jerusa.lara@sesisp.org.br (J.L.); marcos.winicius@sesisp.org.br (M.L.); luiz.lins@sesisp.org.br (L.L.); jaime.lansini@sesisp.org.br (J.L.); leandro.regis@sesisp.org.br (L.S.); jose.lino@sesisp.org.br (J.S.); anderson.orodrigues@sesisp.org.br (A.R.); fteixeira@sesisp.org.br (F.T.)

Vertical jump height is a very important ability related to volleyball performance [1]. Thus, squat jump (SJ) and countermovement jump (CMJ) have been extensively applied to assess lower limb muscle power in many volleyball athletes. However, due to a highest movement pattern related to volleyball, the CMJ with arm swing (CMJa), the block jump (BL) and the spike jump (SP) have been introduced to evaluate volleyball players with more specificity. Overall, upper jump height has been reported in specific jumps (i.e., BL and SP) rather than in general jumps (SJ and CMJ) [2]. In addition to specificity, the competitive level and experience have also been suggested as important factors related to vertical jump performance [3,4]. With this in mind, we assume that professional volleyball players (PRO) may demonstrate a higher jump height than juniors (JUN) whilst the most specific jump tasks would be higher than the general ones. However, the performance of specific and general vertical jump is not completely understood in elite male volleyball athletes. Therefore, the aim of the present study was to compare jump height performance in general and specific vertical jump tests in both PRO and JUN. Overall, 29 elite Brazilian male volleyball players (PRO n = 10; JUN n = 19) from the same club volunteered to participate in the study. Both JUN and PRO team squads are ranked in the top three at the national level. The results of vertical jump tests for PRO and JUN were SJ = 39.33 ± 4.94 and 33.83 ± 5.72 cm, CMJ = 40.80 ± 5.46 and 37.69 ± 6.39 cm, CMJa = 47.98 ± 6.62 and 46.12 ± 7.32 cm, BL = 42.43 ± 6.76 and 40.96 ± 7.46 cm, and SP = 61.73 ± 8.89 and 57.41 ± 9.58 cm, respectively. The two-tailed unpaired Student’s *t*-test revealed that PRO players had a higher SJ than JUN (*p* ≤ 0.05). The one-way ANOVA showed that JUN had significant differences in all vertical jump tests (SP > BL > CMJa > CMJ > SJ; *p* < 0.0001) whereas PRO showed the highest jump height in SP in comparison to all the other vertical jumps (*p* < 0.01). PRO also showed significant higher BL than CMJa (*p* < 0.01) and higher CMJa than both CMJ and SJ (*p* < 0.01). According to our prior hypothesis, the SP jump had the highest jump height in both PRO and JUN. On the other hand, in contrast to our previous assumption, the PRO players showed better results than JUN only in the SJ. In conclusion, these results suggest that JUN and PRO had different vertical jumping ability in concentric-only muscle action such as SJ.

**Funding:** This research received no external funding.

**References**

1. Berriel, G.P.; Schons, P.; Costa, R.R.; Oses, V.H.S.; Fischer, G.; Pantoja, P.D.; Peyré-Tartaruga, L.A. Correlations between jump performance in block and attack and the performance in official games, squat jumps, and countermovement jumps of professional volleyball players. *J. Strength Cond. Res*. **2021**, *35*, S64–S69. https://doi.org/10.1519/JSC.0000000000003858.
2. Fuchs, P.X.; Mitteregger, J.; Hoelbling, D.; Menzel, H.J.K.; Bell, J.W.; von Duvillard, S.P.; Wagner, H. Relationship between general jump types and spike jump performance in elite female and male volleyball players. *Appl. Sci*. **2021**, *11*, 1105. https://doi.org/10.3390/app11031105.
3. Sattler, T.; Hadžic, V.; Derviševic, E.; Markovic, G. Vertical jump performance of professional male and female volleyball players: Effects of playing position and competition level. *J. Strength Cond. Res*. **2015**, *29*, 1486–1493. https://doi.org/10.1519/JSC.0000000000000781.
4. Sheppard, J.M.; Nolan, E.; Newton, R.U. Changes in strength and power qualities over two years in volleyball players transitioning from junior to senior national team. *J. Strength Cond. Res*. **2012**, *26*, 152–157. https://doi.org/10.1519/JSC.0b013e31821e4d5b.

### 2.2. The Performance of Eccentric Hamstring Muscle Strength Produced by Soccer Players throughout the Competitive Season

**Laura Alberti Zandavalli ^1^, Rafael Grazioli ^1^, Filipe Veeck ^1^, Felipe Xavier de Lima e Silva ^2^, Gabriel dos Santos Oliveira ^2^, Bruno Manfredini Baroni ^2^, Ronei Silveira Pinto ^1^ and Eduardo Lusa Cadore ^1^**

^1^ Universidade Federal do Rio Grande do Sul; laurazandavalli@outlook.com (L.A.Z.); rafael_grazioli@hotmail.com (R.G.); filipeveeck.ufrgs@gmail.com (F.V.); ronei.pinto@ufrgs.br (R.S.P.); edcadore@yahoo.com.br (E.L.C.)

^2^ Universidade Federal de Ciências da Saúde de Porto Alegre; fxavierfisioterapia@gmail.com (F.X.L.S.); gabrieldsoliveira26@gmail.com (G.S.O.); bmbaroni@yahoo.com.br (B.M.B.)

Hamstring eccentric muscle strength plays an important role in muscle injuries prevention and physical performance of soccer athletes [1,2]. However, athletes may have to play more than 70 games in a season, playing three games a week on a congested calendar, having little recovery time at baseline levels [3]. Consequently, they present cumulative fatigue, which may result in changes in eccentric muscle strength, generating performance impairment and increasing the risk of injuries [4]. From this perspective, the objective of the present study was to describe and compare the performance of hamstring eccentric strength in soccer players throughout a competitive season in a professional soccer team, in order to verify whether it undergoes variation across the season. We included 20 professional soccer athletes who compete in national competitions and who were evaluated in two moments: at the beginning of the pre-season; and, in the middle of the competitive season. The athletes’ characteristics were age = 25.9 ± 5.4 years old; height = 179.2 ± 8.4 cm; body mass = 79.3 ± 9.7 kg; and, body fat percentage = 11.7 ± 2.4%. The evaluation of hamstring eccentric strength occurred through Nordic Exercise testing on a personalized device, which uses load cells with simultaneous data transfer via bluetooth. For the statistical analysis, the Shapiro–Wilk test was used for normality testing, and the paired *t*-test for comparison between different moments of the season. The effect size (ES) was calculated using Cohen’s d represented by the following formula ES = (MeanPost − MeanPre)/StandardDeviationPre, with MeanPost being the average value of the middle of the competitive season, MeanPre being the average value of the pre-season and StandardDeviationPre the standard deviation pre-season. Comparing to pre-season values, there was a significant reduction in the absolute hamstring eccentric strength (−6.0 ± 11.3%; ES = −0.41; *p* = 0.014) as well as a trend toward a significant decrease in the relative hamstring eccentric strength (−5.2 ± 19.3%; ES = −0.54; *p* = 0.09) in the middle of the season. In summary, the soccer players’ hamstring eccentric strength decreased during the competitive season, which may be a consequence of the cumulative residual fatigue due to the excess of games throughout the year. This cumulative fatigue can induce greater biochemical changes such as muscle damage as well changes in game performance, in addition to exacerbating the risk of injuries.

**Funding:** This research was funded by The National Council for Scientific and Technological Development (CNPq, Brazil) and the Coordination for the Improvement of Higher Education Personnel (CAPES, Brazil).

**References**

1. Timmins, R.G.; Bourne, M.N.; Shield, A.J.; Williams, M.D.; Lorenzen, C.; Opar, D.A. Short biceps femoris fascicles and eccentric knee flexor weakness increase the risk of hamstring injury in elite football (soccer): a prospective cohort study. *Br. J. Sports Med.* **2016**, *50*, 1524–1535. https://doi.org/10.1136/bjsports-2015-095362.
2. Petersen, J.; Thorborg, K.; Nielsen, M.B.; Budtz-Jørgensen, E.; Hölmich, P. Preventive effect of eccentric training on acute hamstring injuries in men’s soccer: a cluster-randomized controlled trial. *Am. J. Sports Med.* **2011**, *39*, 2296–2303. https://doi.org/10.1177/0363546511419277.
3. Mohr, M.; Draganidis, D.; Chatzinikolaou, A.; Barbero-Álvarez, J.C.; Castagna, C.; Douroudos, I.; Avloniti, A.; Margeli, A.; Papassotiriou, I.; Flouris, A.D.; Jamurtas, A.Z.; Krustrup, P.; Fatouros, I.G. Muscle damage, inflammatory, immune and performance responses to three football games in 1 week in competitive male players. *Eur. J. Appl. Physiol.* **2016**, *116*, 179–193. https://doi.org/10.1007/s00421-015-3245-2.
4. Silva, J.R.; Rumpf, M.C.; Hertzog, M.; Castagna, C.; Farooq, A.; Girard, O.; Hader, K. Acute and Residual Soccer Match-Related Fatigue: A Systematic Review and Meta-analysis. *Sports Med.* **2018**, *48*, 539–583. https://doi.org/10.1007/s40279-017-0798-8.

### 2.3. Relationship between Localized, Total % of Fat and the Reduction of Performance in Judo Athletes

Izabela Aparecida dos Santos ^1,2^, Gabriel Felipe Arantes Bertochi ^1^ and Enrico Fuini Puggina ^1,^*

^1^ Post-Graduate Program in Rehabilitation and Functional Performance, Medicine School of Ribeirão Preto, University of São Paulo, São Paulo, Brazil; izabelaeduca94@hotmail.com (I.A.S.); gabrielbertochi1@gmail.com (G.F.A.B.)

^2^ Exercise Physiology in Health and Human Performance Research Group, Department of Physical Education, University of Uberaba (UNIUBE)

* Correspondence: enrico@usp.br (E.F.P.)

Judo is a combat sport with an intermittent physical characteristic, characterized by high intensity with short recovery periods. The total time of the combat is 4 min, which is considered relatively short; however, the physiological demands are high [1]. To achieve a satisfactory performance in judo, athletes need high levels of muscle strength and power, in addition to cardiorespiratory endurance [2]. At competitive level, Judo athletes are categorized by weight, making anthropometric characteristics important for performance in judokas [3,4]. Exploring the relationship between performance tests and anthropometric data can help to understand how these variables are related and consequently, their importance for sport; therefore, the objective of this study was to correlate the anthropometric profile by body segment and total body fat with the performance. Twenty-five elite judokas—13 men (21 ± 3.8 years; 85 ± 16.7 kg; 176 ± 9.3 cm), 12 women (21 ± 3.5 years; 63.5 ± 19.7 kg; 163 ± 10.1 cm)—were evaluated. Initially, they were submitted to dual-energy x-ray absorptiometry (DXA), followed by the countermovement vertical jump (CMJ), squat jump (SJ), force push-ups (FPU) and plyometric push-ups (PPU). Correlations were tested using Pearson’s test. SJ was negatively correlated with leg fat mass (r = −0.763), with torso fat mass (r = −0.663) and with total fat percentage (%fat) (r = −0.864). CMJ was also negatively correlated with leg fat mass (r = −0.751), torso fat mass (r = −0.635), %fat torso (r = −0.770) and %fat total (r = −0.874). In the upper limb tests, FPU was negatively correlated with arm fat mass (r = −0.596), %fat arm (r = −0.788), %fat torso (r = −0.630), and positively with lean arm mass (r = 0.540). Finally, CMP was negatively correlated with arm fat mass (r = −0.531), %fat arm (r = −0.688), torso fat mass (r = −0.428), %fat torso (r = −0.523), total fat mass (r = −0.566) and the %fat total (r = −0.658). Fat mass in different body segments was negatively correlated with the performance in the physical tests, as well as with a positive correlation with lean mass. Therefore, optimizing body composition, especially fat reduction and increase/maintenance of muscle mass can positively influence the performance in judokas.

**Funding:** This research was funded by Coordination for the Improvement of Higher Education Personnel (CAPES), grant number (88887.684581/2022-00).

**References**

1. Franchini, E.; Artioli, G.G.; Brito, C.J. Judo Combat: Time-Motion Analysis and Physiology. *Int. J. Perform. Anal.* **2013**, *13*, 624–641.
2. Franchini, E.; Del Vecchio, F.B.; Matsushigue, K.A.; Artioli, G.G. Physiological Profiles of Elite Judo Athletes. *Sports Med.* **2011**, *41*, 147–166. https://doi.org/10.2165/11538580-000000000-00000.
3. Prieske, O.; Chaabene, H.; Gäbler, M.; Herz, M.; Helm, N.; Markov, A.; Granacher, U. Seasonal Changes in Anthropometry, Body Composition, and Physical Fitness and the Relationships with Sporting Success in Young Sub-Elite Judo Athletes: An Exploratory Study. *Int. J. Environ. Res. Public Health* **2020**, *17*, E7169. https://doi.org/10.3390/ijerph17197169.
4. Drid, P.; Casals, C.; Mekic, A.; Radjo, I.; Stojanovic, M.; Ostojic, S.M. Fitness and Anthropometric Profiles of International vs. National Judo Medalists in Half-Heavyweight Category. *J. Strength Cond. Res.* **2015**, *29*, 2115–2121. https://doi.org/10.1519/JSC.0000000000000861.

### 2.4. Can Auto-Regulating Horizontal Jump Performance Using Minimal Individual Differences Be Used to Regulate Training Load in Young Soccer Players?

**Rodrigo Aquino ^1^, Breno Bonetti ^1^, Mauro Guerra Junior ^2^, Vinicius Ribeiro Silva ^1^ and Luiz Guilherme Gonçalves ^1,3^**

^1^ LabSport, Post-graduate Program in Physical Education, Centre of Physical Education and Sport (CEFD), Federal University of Espírito Santo, Vitória/ES, Brazil; aquino.rlq@gmail.com (R.A.); brenobonetti94@hotmail.com (B.B.); vinicius-ribeiro12@outlook.com (V.R.S.); goncalves.lgui@gmail.com (L.G.G.)

^2^ Fitness Center Anatomy, Vitória/ES, Brazil; guerrajr2@yahoo.com.br (M.G.J.)

^3^ Department of Physiology, Association Ferroviária of Sports, Araraquara/SP, Brazil

A previous study suggested that vertical jump (i.e., countermovement jump-CMJ) could be used to regulate and monitor training load in young futsal players [1]. In addition, horizontal plyometric training presented a positive effect in the acceleration phase (i.e., 10 m sprint time—determinant in soccer) of high-level young soccer players [2]. Therefore, horizontal jump performance (HJ) could be an alternative method to regulate the training load in young players. This study aimed to determine whether auto-regulating horizontal jump performance using minimal individual difference (MID) could be used to regulate training load in young soccer players. Nineteen Brazilian players (15.5 ± 0.8 y) participated in this study and were randomly assigned to either a regulate group (RG; n = 10) or a control group (CG; n = 9). The participants were familiarized with the HJ and then the HJ distance reliability was quantified to determine the MID [1]. The RG performed 6 weeks of training with the training load regulated by a mean distance of HJ with MID, whereas the CG performed the pre-planned training. The outcome measures included tests for the assessment of jump performance (CMJ and HJ), speed (10–30 m sprint), and maximal running speed (MSR) achieved in the 30–15 Intermittent Fitness Test. Internal load (rating of perceived exertion-based training load—sRPE; version Borg’s CR10) was calculated by multiplying the CR10 scale by training/match duration. The following internal load indicators were calculated: acute-sRPE, monotony-sRPE, and strain-sRPE. Data were analyzed using ANOVA two way to compare the physical measures according to the time (T0: pre-training, T1: mid-training, and T2: post-training) and group (RG and CG). The *t*-test for repeated measures was used to compare the internal load indicators between groups according to each week. A significance level of *p* < 0.05 was adopted. There were no significant differences between groups at baseline in performance measures. HJ increased only for RG (T2 > T0; *p* = 0.042, ES = 0.78, very large). In addition, the RG and CG presented higher MSR in T1 (*p* < 0.001, ES = 1.20, very large) and T2 (*p* < 0.001, ES = 0.98, very large) compared to T0. The RG presented higher values of jumps during the training period than the CG (*t* = 3.490, *p* = 0.007, ES = 1.52, very large). In conclusion, the RG improved HJ distance more than traditional preplanned periodized training in young soccer players. Researchers and practitioners could use this auto-regulating method to regulate the training load in young soccer players.

**Funding:** This research received no external funding.

**References**

1. Claudino, J.G.; Cronin, J.B.; Mezêncio, B.; Pinho, J.P.; Pereira, C.; Mochizuki, L.; Amadio, A.C.; Serrão, J.C. Autoregulating Jump Performance to Induce Functional Overreaching. *J. Strength Cond. Res.* **2016**, *30*, 2242–2249. https://doi.org/10.1519/JSC.0000000000001325.
2. Loturco, I.; Pereira, L.A.; Kobal, R.; Zanetti, V.; Kitamura, K.; Abad, C.C.C.; Nakamura, F.Y. Transference Effect of Vertical and Horizontal Plyometrics on Sprint Performance of High-Level U-20 Soccer Players. *J. Sports Sci.* **2015**, *33*, 2182–2191. https://doi.org/10.1080/02640414.2015.1081394.

### 2.5. Does the External Load Vary According to the Match Outcome in Elite-Level Young Brazilian Soccer Players?

**Rodrigo Aquino ^1^, Vinícius Costa ^2^, Rafael José Silva Barbosa ^2^, Gabriel Gazone dos Santos ^2^, Carlos Fabiano Machado ^2^, Luiz Guilherme Gonçalves ^1,3^, Alejandro Pastor ^4^, Eduardo Rostaiser ^4^ and Bruno Pasquarelli ^4^**

^1^ LabSport, Post-graduate Program in Physical Education, Centre of Physical Education and Sport (CEFD), Federal University of Espírito Santo, Vitória/ES, Brazil; aquino.rlq@gmail.com (R.A.); goncalves.lgui@gmail.com (L.G.G.)

^2^ Department of Health and High Performance, Club Regatas of Flamengo, Rio de Janeiro/RJ, Brazil; vinicius.costa@flamengo.com.br (V.C.); rafael.rafael10@hotmail.com (R.J.S.B.); gabriel.gazone@flamengo.com.br (G.G.S.); fabiano.medici@flamengo.com.br (C.F.M.)

^3^ Department of Physiology, Association Ferroviária of Sports, Araraquara, SP, Brazil

^4^ Catapult Group International Ltd., Melbourne, Australia; alejandro.pastor@catapultsports.com (A.P.); eduardo.rostaiser@catapultsports.com (E.R.); bruno.pasquarelli@catapultsports.com (B.P.)

In soccer, distance- and accelerometer-based variables (i.e., total distance and Player Load) have contributed to a better understanding of the external load of match-play [1]. Moreover, in this regard, authors have reported that myriad of factors can influence these parameters (e.g., match outcome—win vs. draw vs. loss) [2]. However, a previous systematic review showed that the potential impact of match contextual factors on external load is frequently overlooked in young players [3]. Therefore, this study aimed to compare the external load parameters according to the match outcome in elite-level young Brazilian soccer players. Fifty male outfield players (U16, n = 18; U17, n = 15; U20, n = 17) were monitored during 15 official matches. The players who participated ≥ 70% of the match time were considered as inclusion criteria. The following variables were obtained using GPS and accelerometer devices (Vector S7, Catapult): (i) total distance covered (meters); (ii) total distance covered in high-speed running (HIR: ≥20 km·h^−1^); (iii) frequency of jumps > 20 cm; vi) frequency of accelerations and decelerations (Acc + Dec; Acc > ^2^ m·s2; Dec < −2 m·s^2^); (iv) Player Load forward (PLfwd; a.u.); (v) Player Load side (PLside; a.u.); (vi) Player Load up (PLup; a.u.); (vii) maximal velocity (km·h^−1^). The match outcome was considered: won (n = 9 matches), draw (n = 2 matches) and loss (n = 4 matches). Separate linear mixed models were performed to compare (fixed effects) match outcome with “position ID” included as a random effect. Multiple comparisons were adjusted using the Bonferroni method. The t-statistics from the mixed models were converted to effect size (ES) correlations and classified as [4]: trivial (r < 0.1), small (r = 0.1–0.3), moderate (r = 0.3–0.5), large (r = 0.5–0.7), very large (r = 0.7–0.9), and almost perfect (r > 0.9). A significance level of *p* < 0.05 was adopted. Data were analyzed using the software Jasp. The main results were (i) total distance covered (F = 0.188; *p* = 0.83), HIR (F = 1.210; *p* = 0.45), jumps > 20 cm (F = 2.110; *p* = 0.14), Acc + Dec (F = 1.157; *p* = 0.41), PLside (F = 2.270; *p* = 0.12), PLup (F = 2.276; *p* = 0.11), and maximal velocity (F = 0.232; *p* = 0.79) does not vary according to the match outcome; (ii) the PLfwd presented greater values in won (355.9 ± 60.7) vs. loss matches (306.56 ± 59.65) (*t* = 3.488, *p* = 0.001, ES = 0.46, moderate). In general, the external load does not vary according to the match outcome in elite-level young soccer players. Coaches and practitioners should consider other contextual factors to interpret the fluctuations of the match external load (e.g., match location, quality of opposition).

**Funding:** This research received no external funding.

**References**

1. Buchheit, M.; Simpson, B.M. Player-Tracking Technology: Half-Full or Half-Empty Glass? *Int. J. Sports Physiol. Perform.* **2017**, *12*, S235–S241. https://doi.org/10.1123/ijspp.2016-0499.
2. Lago-Peñas, C. The Role of Situational Variables in Analysing Physical Performance in Soccer. *J. Hum. Kinet.* **2012**, *35*, 89–95. https://doi.org/10.2478/v10078-012-0082-9.
3. Palucci Vieira, L.H.; Carling, C.; Barbieri, F.A.; Aquino, R.; Santiago, P.R.P. Match Running Performance in Young Soccer Players: A Systematic Review. *Sports Med.* **2019**, *49*, 289–318. https://doi.org/10.1007/s40279-018-01048-8.
4. Rosnow, R.L.; Rosenthal, R.; Rubin, D.B. Contrasts and Correlations in Effect-Size Estimation. *Psychol. Sci.* **2000**, *11*, 446–453. https://doi.org/10.1111/1467-9280.00287.

### 2.6. Calcium Lactate Supplementation Impairs Sprint and RAST Performance in Young Soccer Players

**Henrique Azevedo ^1^, Vitor Azevedo ^1^, Daniel Padilha ^1^, Guilherme Artioli ^2^, David Santos ^3^ and Paulo Azevedo ^1^**

^1^ Universidade Federal de São Paulo; Grupo de Estudos e Pesquisas em Fisiologia do Exercício 1; henrique_azevedo@hotmail.com.br (H.A.); azevedov337@gmail.com (V.A.); daanielpaadilha@hotmail.com (D.P.); paulo.azevedo@unifesp.br (P.A.)

^2^ Centre for Bioscience; Department of Life Sciences; School of Science and Engineering; Manchester Metropolitan University; g.giannini.artioli@mmu.ac.uk (G.A.)

^3^ Universidade Federal de São Paulo; Laboratório de Neurociência e Nutrição (LabNeuN); davidpsantos02@gmail.com (D.S.)

Blood lactate is one of the main energy substrates for cardiomyocytes, skeletal muscle, and the brain [1,2]. Lactate supplementation, in the form of polylactate, sodium lactate, or calcium lactate, has emerged as an alternative to enhance buffering capacity during intense exercises [3] or as the main source of energy [1,2]. In a pilot study conducted in our laboratory, we observed a better performance when soccer players were supplemented with calcium lactate. They executed soccer-specific tasks in less time than control conditions. Thus, we seek to verify whether its ergogenic effect could be reproducible when specific physical tasks are applied. Fifteen soccer athletes (age: 13.6 ± 0.5 years; height: 166.9 ± 7.1 cm; body mass: 57.2 ± 6.8 kg) from the same soccer club participated in this study. The study was approved by the local Ethics Committee (number 5.289.670). This randomized crossover trial consisted of three testing sessions. Before testing, a familiarization session was performed. All sessions were separated for a minimum of 48 h. On the same day, athletes performed Squat Jump (SJ), Countermovement Squat Jump (CMJ), 20 m linear sprint, 20 m zigzag, and running anaerobic speed test (RAST) tests. Between each test, a 5 min interval was provided to allow adequate recovery. The first session served as a control. On each of the experimental sessions, the participants ingested one of the following: a dose of calcium lactate (21.5 mg.kg^−1^ body mass) or a placebo (PL, calcium carbonate, 21.5 mg.kg^−1^ body mass). The treatments were provided in gelatin capsules identical in size, color, and overall appearance. Sixty minutes after the ingestion of the capsules, the participants started the tests. There were no significant differences among conditions for SJ, CMJ, zigzag, and relative minimum power during RAST tests (*p* > 0.05). On the other hand, we observed a significant difference (*p* < 0.05) for 20 m linear sprint, relative peak power, relative mean power, and fatigue index in the RAST test. We conclude that calcium lactate supplementation 60 min before the tasks impairs acceleration, repetitive running sprint ability, and exacerbates fatigue.

**Funding:** This research received no external funding.

**References**

1. Ide, K.; Schmalbruch I.K.; Quistoff B.; Horn A.; Secher N.H. Lactate, glucose and O_2_ uptake in human brain during recovery from maximal exercise. *J. Phys.* **2000**, *22*, 159–164. https://doi.org/10.1111/j.1469-7793.2000.t01-2-00159.xm.
2. Chatam, J.C. Lactate- The forgotten fuel! *J. Phys.* **2002**, *542*, 333. https://doi.org/10.1113/jphysiol.2002.020974.
3. Oliveira, L.F.; Painelli V.S.; Nemezio K.; Gonçalves L.S.; Yamaguchi G.; Gualano S.B.; Artioli G.G. Chronic lactate supplementation does not improve blood buffering capacity and repeated high-intensity exercise. *Scand. J. Med. Sci. Sport* **2017**, *27*, 1231–1239. https://doi.org/10.1111/sms.12792.

### 2.7. Training Characteristics Prior to Personal Best Performances of World-Class Paralympic Swimming Sprinters

Augusto Barbosa ^1,2,^*, Leonardo Araújo ^1^, Thiago Lourenço ^1^ and Renato Barroso ^3^

^1^ Brazilian Paralympic Committee; leonardo.tomasello@cpb.org.br (L.A.); thiago.lourenco@cpb.org.br (T.L.)

^2^ Meazure Sport Sciences

^3^ School of Physical Education, University of Campinas; rbarroso@unicamp.br (R.B.)

* Correspondence: augusto.barbosa@cpb.org.br (A.B.)

This retrospective study described the training characteristics of World-class Paralympic swimming sprinters prior to their personal best performances (PB). Six male (S7, S8, 2 S9s, S10, and S11) and three female swimmers (2 S12s and S14) participated in this study. They reached finals in major international competitions (eight were medalists in individual or relay events), and their PBs were world-ranked among the top-10 in 50 or 100m races (ranging from 1st to 10th). The preparation was divided into four periods [1]: taper (weeks 1 to 2 before competition), short- (SHORT, weeks 3 to 5), medium- (MED, weeks 6 to 8), and long-term (LONG, weeks 9 to 11). The in-water external training load was described by the weekly volume and training intensity distribution (TID) across low-, moderate- and high-intensity volumes, defined according to the coach’s intended prescription. The intensity was determined by the session RPE method with the CR-10 Borg scale [2,3]. Internal training load (ITL) was calculated by multiplying the session RPE score by the session duration in minutes. Monotony (daily mean/standard deviation) and strain (ITL*monotony) were also determined [2,3]. Logistical restrictions prevented the full data collection of four athletes in week 1 (taper). Due to the short duration of the season, three athletes did not present LONG at all. Performance improved 0.68 ± 0.50% (competition time reductions ranged from −1.59 to −0.29%) in comparison with their previous PB. LONG, MED, SHORT and taper presented a volume of 12.9 ± 0.2 km (TID: 62-5-34%), 10.8 ± 1.7 km (TID: 66-2-32%), 10.0 ± 0.9 km (TID: 67-1-32%), 9.5 ± 1.0 km (71-3-26%), an ITL of 2640 ± 176 UA, 2429 ± 687 UA, 2611 ± 663 UA, 2284 ± 689 UA; a monotony of 1.11 ± 0.14, 1.06 ± 0.21, 1.11 ± 0.22, 1.20 ± 0.39; and a strain of 2971 ± 501 UA, 2633 ± 796 UA, 3131 ± 1465 UA, 2831 ± 1612 UA, respectively. One-way ANOVA followed by Bonferroni’s post-hoc test showed a higher volume in LONG than in the other periods (*p* ≤ 0.02). The percentage of low-intensity training was lower in LONG than in taper (*p* < 0.01). The percentage of high-intensity training was lower in taper than in MED and LONG (*p* < 0.05). No differences were found in the percentage of moderate-intensity training, ITL, monotony, and strain across the periods. The low levels of monotony may have favored the adaptations and prevented the potential negative effects of training [3]. World-class Paralympic swimming sprinters present a stable pattern of internal training load parameters prior to their PBs, higher volumes at the early stages of the season and reductions in the high-intensity training loads during taper.

**Funding:** This research received no external funding.

**References**

1. Hellard, P.; Scordia, C.; Avalos, M.; Mujika, I.; Pyne, D. Modelling of optimal training load patterns during the 11 weeks preceding major competition in elite swimmers. *Appl. Physiol. Nutr. Metab.* **2017**, *42*, 1106–1117. https://doi.org/10.1139/apnm-2017-0180.
2. Foster, C.; Florhaug, J.; Franklin, J.; Gottschall, L.; Hrovatin L.; Parker, S.; Doleshal, P.; Dodge, C. A New Approach to Monitoring Exercise Training. *J. Strength Cond. Res.* **2001**, *15*, 109–115.
3. Foster, C. Monitoring training in athletes with reference to overtraining syndrome. *Med. Sci. Sports Exerc.* **1998**, *30*, 1164–1168. https://doi.org/10.1097/00005768-199807000-00023.

### 2.8. Maximum Strength Training in Young Female Wrestling Athletes: A Case Study

**Larissa Bastos ^1^, Giullio da Silva ^2,3,4^, Yuri Silva ^2,3,4^, Andressa dos Santos ^2,3,4^, João de Oliveira ^2,4,5^, Dayane Costa ^1^, Henri Lima ^1^, Rodrigo Vale ^2,3,4,6^ and Vicente Lima ^1,2,3,4^**

^1^ Biodynamics of performance, exercise and health research group (BIODESA/UERJ); larissaruizbastoss@gmail.com (L.B.); dayane.costamarins10@gmail.com (D.C.); henrilima@gmail.com (H.L.); professorvicentelima@gmail.com (V.L.)

^2^ Institute of Physical Education and Sports, State University of Rio de Janeiro, Rio de Janeiro, Brazil. (IEFD/UERJ); giulliocesar.gc@hotmail.com (G.S.); professoryurirolim@gmail.com (Y.S.); professoraoliveira.andressa@gmail.com (A.S.); professorjoaogabrielmdo@gmail.com (J.O.); rodrigogsvale@gmail.com (R.V.)

^3^ Exercise and Sport Laboratory, Institute of Physical Education and Sports, State University of Rio de Janeiro, Rio de Janeiro, Brazil (LABEES/UERJ) 

^4^ Postgraduate Program in Exercise and Sport Sciences, State University of Rio de Janeiro, Rio de Janeiro, Brazil (PPGCEE/UERJ)

^5^ Laboratory of Philosofical Themes in Knowledge Applied to Physical Education and Sports, Institute of Physical Education and Sports, State University of Rio de Janeiro, Rio de Janeiro, Brazil (LAFIL/UERJ) 

^6^ Estácio de Sá University, Exercise Physiology Laboratory, Cabo Frio, Rio de Janeiro, Brazil (LAFIEX/UNESA)

Since its entry into the Olympic Games in 2004, the number of women wrestling practitioners has increased [1]. Wrestling is a combat modality that involves a series of repeated movements with the aim of knocking down the opposing fighter to receive a score [2]. The ability to produce strength is important for the application of attack and defense techniques, proven by the strong correlation between strength and performance [3]. This case study describes the fluctuations in performance following a four-week training cycle during the season that combined a maximum strength training program (five sets of five repetition-maximum load at 3 min of rest interval) with wrestling-specific sessions on neuromuscular variables and VO_2max_. This study is described as a case study in order to establish unique characteristics of a given conditioning [4]. The sample consisted of two 22-years-old female wrestling athletes, who train at a training center located in the south zone of the Rio de Janeiro, with 69.25 ± 11.53kg of total body mass and 1.64 ± 0.02m of height. The exercises used on the two first training weeks were bent over close grip row, squat, bench press, crunch, trunk rotation and biceps curl, in that execution order. The exercises used on the two last training weeks were bent over wide grip row, deadlift, shoulder press, dorsal extension, oblique crunch and biceps curl, in that execution order. Testing was performed before and after the four weeks of training. Tests included the 1 min push up (1PU), 1 min crunch (1C), 1 min pull up (1PL), vertical jump without countermovement (SQ), with countermovement (CMJ) and with free arms (Abalakov), Yo yo recovery 1 (YR1) and sit and reach (SR). There were improvements in neuromuscular tests results: 1C (14.66%), 1PU (11.71%) e SJ (7.34%). There were also improvements in the parameters of distance (20.39%), level (3.41%) and VO_2max_ (2.77%) of the YR1 test. The 1PL test showed no pre- and post-intervention change. The CMJ (−3.79%), Abalakov (−12.13%) and SR (−1.26%) tests showed a reduction in percentage values. From these results, it is possible to conclude that positive performance changes were observed following the four-week maximum strength training program in 1C, 1PU, SQ and in all YR1 parameters.

**Funding:** This research received no external funding.

**References**

1. Zi-Hong, H.; Lian-Shi, F.; Hao-Jie, Z.; Kui-Yuan, X.; Feng-Tang, C.; Da-Lang, T.; et al. Physiological profile of elite Chinese female wrestlers. *J. Strength Cond. Res.* **2013**, *27*, 2374–2395. https://doi.org/10.1519/JSC.0b013e31827f543c.
2. Dehnou, V.V.; Azadi, S.; Gahreman, D.; Doma, K. The effect of a 4-week core strengthening program on determinants of wrestling performance in junior Greco-Roman wrestlers: a randomized controlled trial. *J. Back Musculoskelet. Rehabil.* **2020**, *33*, 423–430. https://doi.org/10.3233/BMR-181328.
3. Basar, S.; Duzgun, I.; Guzel, N.A.; Cicioglu, I.; Çelik, B. Differences in strength, flexibility, and stability in freestyle and Greco-Roman wrestlers. *J Back Musculoskelet Rehabil* **2014**, *27*, 321–330. https://doi.org/10.3233/BMR-130451.
4. Thomas, J.R.; Nelson, J.K.; Silverman, S.J. *Métodos de Pesquisa em Atividade Física*, 6th ed.; Artmed: Porto Alegre, Brazil, 2012; p. 40.

### 2.9. Correlations between Speed of Displacement, Maximum Oxygen Consumption and Fatigue Index in Young Male Football Athletes

Larissa Bastos ^1,^*, Yuri Silva ^2,3^, Théo Pinto ^1^, Anderson Cantuária ^3^, Giullio da Silva ^2,3^, Dayane Costa ^1^, Rodolfo Nunes ^2,3^ and Vicente Lima ^1,2,3^

^1^ Biodynamics of Performance, Exercise and Health Research Group, State University of Rio de Janeiro (UERJ); Rio de Janeiro-RJ, Brazil; theosilva83@hotmail.com (T.P.); dayane.costamarins10@gmail.com (D.C.); professorvicentelima@gmail.com (V.L.)

^2^ Institute of Physical Education and Sports (IEFD), Graduate Program in Exercise and Sports Science (PPGCEE), State University of Rio de Janeiro (UERJ); Rio de Janeiro-RJ, Brazil; professoryurirolim@gmail.com (Y.S.); giulliocesar.gc@hotmail.com (G.S.); rodolfoalkmim@gmail.com (R.N.)

^3^ Laboratory of Exercise and Sport (LABEES), State University of Rio de Janeiro (UERJ); Rio de Janeiro-RJ, Brazil; andersylvacantu@gmail.com (A.C.)

* Correspondence: larissaruizbastoss@gmail.com (L.B.) 

Football is recognized as a long-term intermittent sport in which athletes are in constant motion with and without ball as sprints, side and back displacements, jumps and braking [1]. Soccer athletes aged between 14 and 15 years run an average of 7 to 9 km per match, showing that they must have adequate physical preparation in order to delay the onset of fatigue during the game [2]. Therefore, the present investigation aims to correlate displacement speed, maximum oxygen consumption (VO_2max_) and fatigue index (FI) in young male soccer athletes. Twelve under-15 soccer athletes (age: 14–15 years, height: 1.71 ± 0.09 m, body mass: 61.79 ± 5.59 kg) of a team located in the city of Rio de Janeiro participated in this research. Height, total body mass and the Yoyo recovery test level 1 (YR1) data were collected in the training center on the first visit. From the distance covered in YR1, the VO_2max_ was calculated using the formula proposed by Bangsbo et al. (2008) [3]. On the second visit, the anaerobic power tests (rast test) were applied, which estimates the fatigue index (FI) through the formula proposed by Zagatto et al. (2009) [4]. Also realized was the 20 m speed test (V20m), with 20 min interval between them. The descriptive results point to VO_2max_ values of 44.9 ± 2.2 Lo2/min considered regular for the studied sample, IF with values of 40.3 ± 11.2% considered weak, demonstrating a need to improve the anaerobic capacity for intermittent efforts, and the values found for V20m were 3.39 ± 0.17s. There was no correlation between FI and V20m (r = 0.280; *p* = 0.378), FI and VO_2max_ (r = −0.153; *p* = 0.635) and V20m and VO_2max_ (r = 0.037; *p* = 0.910). In conclusion, it can be observed that this sample of young male soccer athletes displayed regular levels of VO_2max_, despite these not interfering with the ability to sustain anaerobic efforts.

**Funding:** This research received no external funding.

**References**

1. Castillo, D.; Raya-González, J.; Weston, M.; Yanci, J. Distribution of external load during acquisition training sessions and match play of a professional soccer team. *J. Strength Cond. Res.* **2019**, *35,* 3453–3458. https://doi.org/10.1519/JSC.0000000000003363.
2. Buschheit, M.; Mendez, A.; Simpson, B.M.; Bourdon, P.C. Match running performance and fitness in your soccer. *Int. J. Sports Med.* **2010**, *31*, 818–825. https://doi.org/10.1055/s-0030-1262838.
3. Bangsbo, J.; Iaia, F.M.; Krustrup, P. The Yo-Yo intermittent recovery test. *Sports Med.* **2008**, *38*, 37–51. https://doi.org/10.2165/00007256-200838010-00004.
4. Zagatto, A.M.; Beck, W.R.; Gobatto, C.A. Validity of the running anaerobic sprint test for assessing anaerobic power and predicting short-distance performances. *J. Strength Cond. Res.* **2009**, *23*, 1820–1827. https://doi.org/10.1519/JSC.0b013e3181b3df32.

### 2.10. Acute Effects of Concurrent Strength and Endurance Training at Different Intensities in Recreational Athletes

Beltrán Cáceres ^1,^*, Tomás T. Freitas ^1,2,3,4,5^, Cristian Marín-Pagán ^2^, Antonio Martínez-Serrano ^2,3^ and Pedro E. Alcaraz ^2,3^

^1^ Facultad de Deporte, UCAM Universidad Católica de Murcia, Murcia, Spain; tfreitas@ucam.edu (T.T.F.)

^2^ UCAM Research Center for High Performance Sport, UCAM Universidad Católica de Murcia, Murcia, Spain; cmarin@ucam.edu (C.M.P.); amartinez30@ucam.edu (A.M.S.); palcaraz@ucam.edu (P.E.A.)

^3^ Strength & Conditioning Society, Murcia, Spain

^4^ NAR-Nucleus of High Performance in Sport, São Paulo 04753060, Brazil

^5^ Department of Human Movement Sciences, Federal University of São Paulo, Santos, Brazil

* Correspondence: beltran_2301@hotmail.com (B.C.)

The control of variables in concurrent training plays a very important role in neuromuscular and metabolic adaptations [1,2,3]; among which, intensity is responsible for causing more or less physiological adaptations at a central or peripheral level [4]. The purpose of this research was to quantify the neuromuscular and peripheral fatigue produced by the concurrent training of strength and endurance at different intensities in order to predict the most favorable conditions for experiencing the interference phenomenon in relation to the variable intensity. Twelve amateur athletes (age: 23.6 ± 2.61 years, height: 178 ± 6.26 cm, weight: 73.5 ± 8.74 kg) undertook four concurrent training protocols (P) at different intensities (P1: 10RM + 25’ at 75%VO_2max_, P2: 5RM + Repeated sprint training (RST) of 24 sprint at 100% maximal sprinting speed (MSS), P3: 5RM + 25’ at 75%VO_2max_, P4: 10RM + RST of 24 sprint at 100% MSS) in different weeks. Pre, post and 24 h post-training were evaluated in function of the variables of countermovement jump, jump height from take off (Hmax), relative maximal power (Pmax), force at peak eccentric, force at peak concentric and braking time; in addition to the magnitude of central fatigue with its variables of absolute maximum voluntary contraction (MVC), peak torque and voluntary activation, and residual peripheral fatigue (RPF) at different frequencies (1, 10, 20, 20, 50 and 100 Hz) through the interpolation twitch technique. Physiological parameters of resting heart rate, systolic blood pressure and diastolic blood pressure, body mass and the rating of fatigue scale, were also evaluated. Results demonstrated a significant decrease in Hmax pre–post (*p* = 0.011) in all groups only exceeding the initial value of the studio in P2 (↑0.29%). Pmax values decreased in all groups and recovered almost completely only at P2 (↓0.19%) after 24 h. A significant decrease in post MVC values (*p* = 0.05) occurred in all groups, being higher in P2 (↓8.36%) and P4 (↓12.14%). RPF10 significantly decreased pre–post (*p* = 0.004) and pre–post 24 h (*p* = 0.025), to a higher degree and identically in P2 and P4 (↓21.64% and ↓10. 45%), while RPF100 decreased significantly in pre–post (*p* < 0.001) and pre–post 24 h (*p* = 0.005) to a greater degree in P2 (↓20.71% and ↓10%) and P4 (↓20.29% and ↓7.97%). In conclusion, concurrent training intensity influences both central and peripheral adaptations, which will prolong recovery time to a greater or lesser extent.

**Funding:** This research received no external funding.

**References**

1. Methenitis, S. A brief review on concurrent training: from laboratory to the field. *Sports* **2018**, *6*, 127. https://doi.org/ 10.3390/sports6040127.
2. Wilson, J.M.; Marin, P.J.; Rhea, M.R.; Wilson, S.M.C.; Loenneke, J.P.; Anderson, J.C. Concurrent training: a meta-analysis examining interference of aerobic and resistance exercises. *J. Strength Cond. Res.* **2012**, *26*, 2293–2307. https://doi.org/10.1519/JSC.0b013e31823a3e2d.
3. Fyfe, J.; Bishop, D.; Stepto, N. Interference between concurrent resistance and endurance exercise: molecular bases and the role of individual training variables. *Sports Med.* **2014**, *44*, 743–762. https://doi.org/10.1007/s40279-014-0162-1.
4. Docherty, D.; Sporer, B. A proposed model for examining the interference phenomenon between concurrent aerobic and strength training. *Sports Med.* **2000**, *30*, 385–394. https://doi.org/10.2165/00007256-200030060-00001.

### 2.11. The Effect of a Long-Term Training Program on the Physical Fitness of Brazilian Professional Judo Athletes

**Bruno Campos, Alexandre Lee, Aline Tritto, Danilo Jesus, Marcos Ignácio, Victor Leopoldo, Fabiano Teixeira and Cesar Abad**

Reference Center of Sport Science of Social Service of Industry (CRCE-SESI); bruno.campos@sesisp.org.br (B.C.); alexandre.lee@sesisp.org.br (A.L.); aline.tritto@sesisp.org.br (A.T.); danilo.jesus@sesisp.org.br (D.J.); marcos.inacio@sesisp.org.br (M.I.); victor.leopoldo@sesisp.org.br (V.L.); fteixeira@sesisp.org.br (F.T.); cesar.abad@sesisp.org.br (C.A.) 

Judo may be considered an intermittent sport that requires energy from both aerobic and anaerobic systems [1,2]. During official judo combat, high aerobic endurance capacity, muscular endurance, muscular strength, and power are required [3]. Therefore, the training program must contain multiple exercises to develop specific match skills and physical fitness related to match performance to improve judo performance [4]. The effect of a long-term training program (>4 weeks) on upper and lower limb performance in professional judo athletes remains underdescribed in the literature. Thus, the present study aimed to verify the physical performance variations of judo athletes during a competitive period. Eleven male professional judo athletes (19 ± 1 years; 78.9 ± 16.7 kg; 174.9 ± 13.0 cm) volunteered to participate in the study. The training program lasted 8 weeks and comprised 72 training sessions (48 technical-tactical and 24 strength and power). The strength training session was based on undulating strength training periodization. The session was distributed into maximal strength exercises, power exercises, plyometrics, and ballistic exercises. The vertical jumps (SJ and CMJ); the maximal power output for half-squat (HS), bench-press (BP), and prone-row (PR) exercises; the repetitions of pull-down (PD) with the Kimono; and the Hikidashi test (HK) were assessed prior (PRE) and 8 weeks after (POST) the training program. Paired Student’s *t*-Tests were performed to analyze the pre and post-assessment. Significant increases were noted for the power outputs in the HS and PR exercises (HSpre = 1886 ± 189 W; HSpost = 2553 ± 357 W (*p* < 0.0001); PRpre = 2038 ± 314 W; PRpost: 2548 ± 329 W; *p* < 0.0001) when comparing pre-and post-assessments. No significant changes were noticed for the other variables tested [SJpre = 33.1 ± 5.2 cm; SJpost = 32.7 ± 3.9 cm (*p* = 0.77); CMJpre = 35.7 ± 4.8 cm; CMJpost = 35.7 ± 5.9 cm (*p* = 0.9); PDpre = 18.3 ± 6.6; PDpost = 18.2 ± 4.4 (*p* = 0.39); HKpre = 45.5 ± 2.3; HKpost = 46.3 ± 4.7 (*p* = 0.90)]. In conclusion, our results showed that muscle power varied positively after 8 weeks of training. However, variables such as VJ and specific judo tasks did not vary significantly over 8 weeks. The muscle power of HS and PR exercises seems more responsive to changes than VJ and specific judo tasks during a long-term training program. This finding may help judo professionals think about tests to monitor performance throughout a competitive season.

**Funding:** This research received no external funding.

**References**

1. Franchini, E.; Panissa, V.L.; Julio, U.F. Physiological and performance responses to intermittent Uchi-komi in Judo. *J. Strength Cond. Res.* **2013**, *27*, 1147–1155. https://doi.org/10.1519/JSC.0b013e3182606d27.
2. Julio, U.F.; Panissa, V.L.; Esteves, J.V.; Cury, R.L.; Agostinho, M.F.; Franchini, E. Energy-system contributions to simulated judo matches. *Int. J. Sport. Physiol. Perform.* **2017**, *12*, 676–683. https://doi.org/10.1123/ijspp.2015-0750.
3. Kostrzewa, M.; Laskowski, R.; Wilk, M.; Błach, W.; Ignatjeva, A.; Nitychoruk, M. Significant predictors of sports performance in elite men judo athletes based on multidimensional regression models. *Int. J. Environ. Res. Public Health* **2020**, *17*, 8192. https://doi.org/10.3390/ijerph17218192.
4. Franchini, E.; Branco, B.M.; Agostinho, M.F.; Calmet, M.; Candau, R. Influence of linear and undulating strength periodization on physical fitness, physiological, and performance responses to simulated judo matches. *J. Strength Cond. Res.* **2015**, *29*, 358–367. https://doi.org/10.1519/JSC.0000000000000460.

### 2.12. Biomechanical Properties of Quadruped Exercises Performed by Healthy Women

Patrícia Cardoso Clemente ^1,2^, Bruno Pascoalini da Silva ^1^, Luane Landim de Almeida ^1,2^, Eduardo José Danza Vicente ^3^, Diogo Simões Fonseca ^1,3^, Victor Hugo Souza ^1,4,5^, Diogo Carvalho Felício ^1^ and Marco Antonio Cavalcanti Garcia ^1,5,6,^*

^1^ Programa de Pós-Graduação em Ciências da Reabilitação e Desempenho Físico Funcional, Faculdade de Fisioterapia, Universidade Federal de Juiz de Fora, Minas Gerais, Brazil; profpatriciacardosojf@gmail.com (P.C.C.); bruno_pascoalini@yahoo.com.br (B.P.S.); lua_landim@hotmail.com (L.L.A.); diogo.simoes@ufjf.br (D.S.F.); victor.souza@aalto.fi (V.H.S.); diogofelicio@yahoo.com.br (D.C.F.)

^2^ Faculdade de Ciências Médicas e da Saúde de Juiz de Fora (SUPREMA), Juiz de Fora, Minas Gerais, Brazil

^3^ Departamento de Fisioterapia Cardiorespiratória e Musculoesquelética, Faculdade de Fisioterapia, Universidade Federal de Juiz de Fora, Minas Gerais, Brazil; eduardo.vicente@ufjf.br (E.J.D.V.) 

^4^ Department of Neuroscience and Biomedical Engineering, Aalto University, School of Science, Espoo, Finland

^5^ Grupo de Neuro Biomecânica, Faculdade de Fisioterapia, Universidade Federal de Juiz de Fora, Minas Gerais, Brazil

^6^ Departamento de Fisiologia, Instituto de Ciências Biológicas, Universidade Federal de Juiz de Fora, Minas Gerais, Brazil

* Correspondence: marco.garcia@ufjf.br (M.A.C.G.)

Different symmetrical and asymmetrical quadruped exercises (QE) have been widely adopted in rehabilitation and sports programs [1,2,3,4], although there seems to be a lack of understanding about their suitability. This study investigated the perceived exertion, postural demands, and muscle recruitment profiles imposed by three different QE postures. Thirty healthy women (22.1 ± 1.55 years old; 1.60 ± 0.06 m; body mass: 54.4 ± 9.02 kg; laterality score: +80.4 ± 33.8) participated in the study. The local ethics committee approved the study (n. 2.634.323). The QE postures were investigated under isometric contraction while the participants maintained the right (dominant) hand on the ground. They performed the following three QE postures: a. Classic Quadruped (CQ): 180° shoulder flexion with external shoulder rotation and forearm in the neutral position. Contralateral hip extension up to 0° with maximum plantar flexion; b. Functional quadruped (FQ): maximal extension of the upper limb and contralateral lower limb extension to 0° and maximum plantar flexion; Homolateral quadruped (HQ): same as the classic, but the homolateral lower limb and upper limb. Each QE posture was performed three times for 10″ each. The Borg scale (BS) was used at the end of each attempt to obtain the perceived exertion index from each participant. The elliptical center of pressure (CoP) area from the statokinesiogram was used to evaluate how much the quadrupedal postural stability control challenged the palmar support base. The surface myoelectric activity (sEMG) of four different trunk muscles (*transverse abdominis*; *iliocostalis lumborum*; *longissimus dorsi*; and *multifidus*, bilaterally) was recorded for analysis. Higher values were found for the BS in HQ (4.35 ± 1.8) than in the other two QE (CQ: 2.1 ± 0.8; FQ: 2.9 ± 1.6; *p* < 0.05). Concerning the elliptical CoP area, HQ presented significantly (*p* < 0.05) greater areas ($\bar{x}$ = 89.06 mm^2^) than CQ ($\bar{x}$ = 57.01 mm^2^) and FQ ($\bar{x}$ = 63.90 mm^2^). In relation to the sEMG signal, it was significantly greater for CQ in contrast to HQ (*p* = 0.01) but not in relation to FQ (*p* = 0.36). There was no interaction among the three factors (QE × Hemibody Side × Muscle; *p* = 0.86). Our findings suggest that HQ was the most challenging exercise regarding CoP and BS, although the magnitude of sEMG did not follow the BS. Interestingly, CQ presented a higher symmetrical myoelectrical activity. Understanding the characteristics of QE may help health professionals to prescribe them more suitably.

**Funding:** This study was financed in part by the [Coordenação de Aperfeiçoamento de Pessoal de Nível Superior—Brasil (CAPES) #1] under Grant [Finance Code 001], [Fundação de Amparo à Pesquisa do Estado de Minas Gerais (FAPEMIG) #2] under Grant [CDS-APQ-01730-09-51900] and [CDS-APQ-00923-11-213588], and [Conselho Nacional de Desenvolvimento Científico e Tecnológico (CNPq) #3]. VHS was funded by the European Research Council (ERC) under the European Union’s Horizon 2020 research and innovation programme (ConnectToBrain; grant agreement number 810377).

**References**

1. Knox, M.F.; Chipchase, L.S.; Schabrun, S.M.; Marshall, P.W.M. Improved compensatory postural adjustments of the deep abdominals following exercise in people with chronic low back pain. *J*. *Electromyogr*. *Kinesiol*. **2017**, *14*, 133–140. https://doi.org/10.1016/j.jelekin.2017.10.009.
2. Manchikanti, L.; Singh, V.; Falco, F.J.; Benyamin, R.M.; Hirsch, J.A. Epidemiology of Low Back Pain in Adults. *Neuromodulation* **2014**, 17, 3–10. https://doi.org/10.1111/ner.12018.
3. Shah, J.; Tanwar, T.; Iram, I.; Aldabbas, M.; Veqar, Z. Effect of Increased Lumbar Lordosis on Lumbar Multifidus and Longissimus Thoracis Activation During Quadruped Exercise in Patients With Chronic Low Back Pain: An EMG Study. *J*. *Appl*. *Biomech*. **2020**, *36*, 436–443. https://doi.org/10.1123/jab.2020-0040.
4. Gupta, G.; Alok, M. Effectiveness of plank exercise in low back pain. *Int*. *J*. *Sci*. *Res*. **2020**, *9*, 1182–1186. https://doi.org/10.21275/SR201011145832.

### 2.13. Effects of Combined Training on Cold-Stimulated Muscle Glucose Uptake in Individuals with Overweight and Type 2 Diabetes

**Cláudia Cavaglieri ^1^, Ivan Bonfante ^1,2^, Enrico Finardi ^1^, Milena Monfort-Pires ^3^, Renata Duft ^1^, Keryma Mateus ^1^, Sergio Brunetto ^4^, Mara Patricia Chacon-Mikahil ^1^, Licio Velloso ^3^ and Celso Ramos ^4^**

^1^ Laboratory of Exercise Physiology, School of Physical Education, University of Campinas; cavaglieri@fef.unicamp.br (C.C.); ivanlpb@hotmail.com (I.B.); enricofinardi@hotmail.com (E.F.); renataduft@gmail.com (R.D.); keryma.chaves@gmail.com (K.M.); marapatricia@fef.unicamp.br (M.P.C.M.) 

^2^ Federal Institute of Education, Science and Technology of São Paulo

^3^ Laboratory of Cell Signaling, Department of Internal Medicine, University of Campinas; monfortpiresm@gmail.com (M.M.P.); lavelloso.unicamp@gmail.com (L.V.) 

^4^ Department of Radiology, University of Campinas; serqbru@gmail.com (S.B.); cdramos@unicamp.br (C.R.)

Combined training (CT) has been shown to be a non-pharmacological treatment for overweight and type 2 diabetes (T2D) [1,2]. In addition, the increase in thermogenic activity of adipocytes induced by physical training is also associated with metabolic improvement [1]. However, it is not known whether physical training could increase muscle thermogenic activity non-shivering. The goal of present work was to evaluate the effects of CT on cold-stimulated muscle glucose uptake in individuals with overweight and T2D. The sample consisted of 12 individuals with T2D, of both sexes and with a body mass index between 25–35kg/m^2^. In the pre–post experimental period, image analyses were performed by Positron coupled to Computed Tomography (PET-CT) with 18Fluordeoxyglucose (18FDG). The CT program consisted of strength training (1–3 series of 10–12 submaximal repetitions, from 1′–1.15″ interval) followed by aerobic training (35 min at 50–70% of VO_2max_) in the same session, with three sessions weekly, for 16 weeks. In pre- and post-training, an increase was observed in the uptake of 18FDG by the trapezius (right side) (*p* = 0.01) and heart muscles (*p* = 0.01). When comparing standard uptake value (SUV) differences between muscles in pre moment, it was observed that right and left longus colli muscle presents higher SUV than all skeletal muscles (*p* < 0.01 for all analyses). The right and left sternocleidomastoid also presented higher SUV than the right trapezius (*p* = 0.01; *p* = 0.04), left vastus lateralis (*p* = 0.02; *p* = 0.04), right (*p* = 0.01, *p* = 0.01) and left (*p* = 0.01; *p* = 0.03) biceps femoris, and right (*p* = 0.02; *p* = 0.04) and left (*p* = 0.02; *p* = 0.04) semitendinosus. The heart presented higher SUV than all skeletal muscles (*p* < 0.01 for all analyses), except for right and left longus colli (*p* = 0.45; *p* = 0.99) and right and left sternocleidomastoid (*p* = 0.57; *p* = 0.42), while the aorta artery presented higher SUV than all skeletal muscles (*p* < 0.01 for all analyzes) and heart (*p* < 0.01). Finally, in post moment, higher SUV in right and left longus colli was observed than all skeletal muscles (*p* < 0.01 for analyses). The heart showed higher SUV than all skeletal muscles (*p* < 0.01 for all analyses). The aorta artery presented higher SUV than all skeletal muscles (*p* < 0.01 for all analyses), except for right and left longus colli (*p* = 0.42). CT increases the glycemic uptake of the heart and trapezius muscles induced by exposure to cold. Muscles in the neck region have greater glycemic uptake, which may attribute a thermogenic role to them.

**Funding:** This research was funded by São Paulo State Research Support Foundation (FAPESP)—São Paulo/Brazil—Regular Research Grants—Process: 2016/08751-3; Coordination for the Improvement of Higher Education Personnel (CAPES)—Brazil—Financing Code 001; National Council for Scientific and Technological Development (CNPQ)—Brazil—Financing Code 303571/2018-7.

**References**

1. Colberg, S.R.; Sigal, R.J.; Yardley, J.E.; Riddell, M.C.; Dunstan, D.W.; Dempsey, P.C.; Horton, E.S.; Castorino, K.; Tate, D.F. Physical Activity/Exercise and Diabetes: A Position Statement of the American Diabetes Association. *Diabetes Care* **2016**, *39*, 2065–2079. https://doi.org/10.2337/dc16-1728.
2. Bonfante, I.L.P.; Monfort-Pires, M.; Duft, R.G.; da Silva Mateus, K.C.; de Lima Júnior, J.C.; dos Santos Trombeta, J.C.; Finardi, E.A.R.; Brunelli, D.T.; Morari, J.; de Lima, J.A.B.; et al. Combined training increases thermogenic fat activity in patients with overweight and type 2 diabetes. *Int. J. Obes. (Lond.)* **2022**, *46*, 1145–1154. https://doi.org/10.1038/s41366-022-01086-3.

### 2.14. Explosive Performance and Lactate Production: Is It Indicative of Muscular Properties in Swimmers?

Francisco Cuenca-Fernández *, Óscar López-Belmonte, Jesús Juan Ruiz-Navarro, Ana Gay, Esther Morales-Ortíz, Gracia López-Contreras and Raúl Arellano

Aquatics Lab, Department of Physical Education and Sport. Faculty of Sports Sciences. University of Granada (Spain); oscarlobel@ugr.es (O.L.B.); jesusruiz@ugr.es (J.J.R.N.); anagay@ugr.es (A.G.); esthermo@ugr.es (E.M.O.); gracia@ugr.es (G.L.C.); r.arellano@ugr.es (R.A.) 

* Correspondence: cuenca@ugr.es (F.C.F.)

Lactate [La^−^] is continuously formed as a product of one metabolic pathway (glycolysis), which acts as a substrate for another pathway (mitochondrial respiration) [1]. In response to the energy crisis in muscle cells, the rate of [La^−^] accumulation increases, as glycolysis has a much higher activity than the oxidative enzymes of mitochondrial respiration [2]. However, this imbalance may be even more pronounced in individuals with a high percentage of fast-twitch fibers due to their higher glycolytic activity [2,3]. In a counterbalanced crossover design, in fourteen competitive swimmers (males: 18.95 ± 1.63; females: 19.02 ± 0.78 years; 100 m freestyle time, males: 56.35 ± 1.44 s; females: 63.01 ± 1.60 s), [La^−^] and countermovement jump (CMJ) height were measured after two swimming training sets. The sets consisted of 10 × 100 m swimming bouts (Race-pace training [RPT]), and 20 × 50 m swimming bouts (Ultra-short race-pace training [USRPT]), and swimmers were given individualized target and recovery times based on specific 200 m times. CMJ-height was assessed 2 min before, and 2 and 5 min after the experimental set, while [La^−^] samples were collected at the same time-points. A two-way repeated measures ANOVA was applied to study the differences in CMJ-height between protocols and time-points. Pearson correlation coefficient (r) was used to verify the associations between [La^−^] and CMJ-height (statistical significance *p* < 0.05). A significant time (F_2,26_ = 22.177, *p* < 0.001), and time × set interaction (F_2,26_ = 6.951, *p* < 0.004) was identified for CMJ-height when relative 2 min pre- to 2 and 5 min post-exercise were compared between the protocols (RPT [2 min pre- vs. 2 min post-, ∆ = −11.09%; vs. 5 min post-, ∆ = −4.94%]) vs. (USRPT [2 min pre- vs. 2min post-, ∆ = −5.89%; vs. 5 min post-, ∆ = 4.24%]), indicating that there was lower neuromuscular fatigue after USRPT to perform an explosive task afterward [4]. The correlation analysis only revealed a positive correlation for females between [La^−^] and CMJ-height after USRPT, both at min 2 (r = 0.89; p = 0.006) and at min 5 (r = 0.80; p = 0.027), thus showing that those producing more [La^−^] performed better in the CMJ test. This relationship may be a consequence of a higher percentage of fast-twitch fibers because exceeding [La^−^] could not be easily managed in the mitochondrial reticulum of those muscle cells (1–3). This procedure constitutes a practical way to monitor the muscular properties of swimmers and could be used to prescribe more individualized swimming training protocols (e.g., RPT or USRPT).

**Funding:** This research received no external funding.

**References**

1. Brooks, G.A. Lactate as a fulcrum of metabolism. *Redox Biol*. **2020**, *35*, 101454. https://doi.org/10.1016/j.redox.2020.101454.
2. Ferguson, B.S.; Rogatzki, M.J.; Goodwin, M.L.; Kane, D.A.; Rightmire, Z.; Gladden, L.B. Lactate metabolism: historical context, prior misinterpretations, and current understanding. *Eur. J. Appl. Physiol*. **2018**, *118*, 691–728. https://doi.org/10.1007/s00421-017-3795-6.
3. Greenwood, J.D.; Moses, G.E.; Bernardino, F.M.; Gaesser, G.A.; Weltman, A. Intensity of exercise recovery, blood lactate disappearance, and subsequent swimming performance. *J. Sports Sci*. **2008**, *26*, 29–34. https://doi.org/10.1080/02640410701287263.
4. Jiménez-Reyes, P.; Pareja-Blanco, F.; Cuadrado-Peñafiel, V.; Ortega-Becerra, M.; Párraga, J.; González-Badillo, J.J. Jump height loss as an indicator of fatigue during sprint training. *J. Sports Sci*. **2019**, *37*, 1029–1037. https://doi.org/10.1080/02640414.2018.1539445.

### 2.15. Maximum Strength Training in Young Male Wrestling Athletes: A Case Study

**Giullio da Silva ^1,2,3^, Yuri Silva ^1,2,3^, João de Oliveira ^1,2,3^, Andressa dos Santos ^1,2,3^, Larissa Bastos ^4^, Dayane Costa ^4^, Henri Lima ^4^, Vicente Pinheiro Lima ^1,2,3,4^ and Rodrigo Vale ^1,2,3,5^**

^1^ Institute of Physical Education and Sports, State University of Rio de Janeiro, Rio de Janeiro, Brazil. (IEFD/UERJ); giulliocesar.gc@hotmail.com (G.S.); professoryurirolim@gmail.com (Y.S.); professorjoaogabrielmdo@gmail.com (J.O.); professoraoliveira.andressa@gmail.com (A.S.); professorvicentelima@gmail.com (V.P.L.); rodrigogsvale@gmail.com (R.V.) 

^2^ Exercise and Sport Laboratory, Institute of Physical Education and Sports, State University of Rio de Janeiro, Rio de Janeiro, Brazil (LABEES/UERJ)

^3^ Postgraduate Program in Exercise and Sport Sciences, State University of Rio de Janeiro, Rio de Janeiro, Brazil (PPGCEE/UERJ) 

^4^ Biodynamics of performance, exercise and health research group (BIODESA/UERJ); larissaruizbastoss@gmail.com (L.B.); dayane.costamarins10@gmail.com (D.C.); henrilima@gmail.com (H.L.)

^5^ Estácio de Sá University, Exercise Physiology Laboratory, Cabo Frio, Rio de Janeiro, Brazil (LAFIEX/UNESA)

Wrestling is a combat modality with a high-intensity interval characteristic [1] in which the success of athletes’ performance is related to parameters such as the ability to produce maximum dynamic strength, isometric strength, power and muscular endurance [2]. This case study analyzes the effect of a four-week maximum strength training program (five sets of five repetitions at 3 min of rest interval) on neuromuscular variables and VO_2max_. The present research is characterized as a case study, which aims to determine unique characteristics of a given condition [3]. The study had as a sample two male wrestling athletes aged 20 ± 1.41 years, who train in a training center located in the Flamengo neighborhood, south zone of city of Rio de Janeiro, with 78.20 ± 13.86 kg of total body mass and 1.74 ± 0.08 m of height. The exercises were performed on the first two training weeks: bent over close grip row, squat, bench press, crunch, trunk rotation and biceps curl, in that order. The following exercises were performed on the last two training weeks: bent over wide grip row, deadlift, shoulder press, dorsal extension, oblique crunch and biceps curl. Before and after the training period, the right (RHG) and left (LHG) handgrip, 1 min crunch (1C), 1 min push up (1PU), 1 min pull up (1PL), squat jump without countermovement (SJ), with countermovement (CMJ) and with arms help (Abalakov), Yoyo recovery 1 (YR1) and sit and reach (SR) tests were performed. There were improvements in absolute values in the RHG (0.7N), LHG (0.2N), SR (12 cm), 1PU (3 repetitions), 1C (2 repetitions) e 1PL (1 repetition). On the contrary, there was a reduction in performance in the SJ (−2.2 cm), CMJ (−0.8 cm), Abalakov (−3.6 cm) and in the parameters of YR1 (−360 m, −1.1 level and −3Lo2/min). Thus, it is possible to conclude that a four-week maximum strength training program improved RHG, LHG, SR, 1PU, 1C and 1CU. However, it reduced the performance of power production capacities of vertical jump tests and in all parameters of the YR1.

**Funding:** This research received no external funding.

**References**

1. Francino, L.; Villaroel, B.; Valdés-Badilla, P.; Ramirez-Campillo, R.; Martín, EB.; Ojeda-Aravena, A.; et al. Effect of a six week in-season training program on wrestling-specific competivive performance. *Int. J. Environ. Res. Public Health* **2022**, *19*, 9325. https://doi.org/10.3390/ijerph19159325.
2. Chaabene, H.; Negra, Y.; Bouguezzi, R.; Mkaouer, B.; Franchini, E.; Julio, U.; et al. Physical and physiological attributes of wrestlers: an update. *J. Strength Cond. Res.* **2017**, *31*, 1411–1442. https://doi.org/10.1519/JSC.0000000000001738.
3. Thomas, J.R.; Nelson, J.K.; Silverman, S.J. *Métodos de Pesquisa em Atividade Física*, 6th ed.; Artmed: Porto Alegre, Brazil, 2012; p. 40.

### 2.16. Impact of the Menstrual Cycle on Physical and Psychological Factors in Elite Academy Women Soccer Players

Tom Douchet ^1,2,^*, Etienne Juillard ^1^, Christos Paizis ^2^ and Nicolas Babault ^2^

^1^ Dijon Football Côte d’Or (DFCO), 17 rue du Stade, 21000 Dijon, France; e.juillard1295@gmail.com (E.J.) 

^2^ Center for Performance Expertise, CAPS, U1093 INSERM, Sport Science Faculty, University of Bourgogne-Franche-Comté, 3 allée des Stades Universitaires, BP 27877, CEDEX, 21078 Dijon, France; christos.paizis@u-bourgogne.fr (C.P.); nicolas.babault@u-bourgogne.fr (N.B.)

* Correspondence: tom.douchet@gmail.com (T.D.); Tel.: +33-3-80-39-67-01

The impact of menstrual cycles on athletic performance has been widely discussed without reaching a consensus. Some authors demonstrated that the estrogen secreted during the menstrual cycle’s follicular phase (FP) favored strength development and aerobic capacities [1,2]. In contrast, other authors have shown that the menstrual cycle (FP and Luteal Phase (LP)) did not influence aerobic, and muscle contractile capacity [3]. Additionally, a recent study highlighted that a majority of women reported through a survey that their menstrual cycle negatively impacted their performances [4]. However, each study independently analyzed physical and psychological indicators without combining them. Furthermore, previous studies analyzed the menstrual cycles through hormone peaks to determine the different phases without considering weekly indicators’ fluctuation. Therefore, the present study aimed to combine physical and psychological markers through weekly monitoring to understand if the menstrual cycle can explain athletic performance fluctuations. Ten elite academy women soccer (age = 18.1 ± 0.4 years) players were monitored for 9 weeks. Players had to report through an online anonymous survey the start and end of their menstrual cycle to determine precisely their different phases. Players were tested twice weekly (on match day + 1 and match day − 2) on the Illinois Agility Test (IAT). Moreover, they answered the Hooper questionnaire (Sleep, Stress, Fatigue, DOMS, Hooper Index) to get an insight into their subjective fitness level. Each player’s menstrual cycle was divided into week 1 and week 2 for FP and week 1 and week 2 of LP. Therefore, two-way repeated measures ANOVAs were performed to evaluate the impact of the different weeks in each phase on physical and psychological factors. Statistical significance was set at *p* < 0.05. ANOVAs results for psychological factors did not demonstrate any significant difference with Sleep (*p* = 0.570), Stress (*p* = 0.437), DOMS (*p* = 0.060), Fatigue (*p* = 0.568), and Hooper Index (*p* = 0.403). Moreover, physical factors, as witnessed by IAT performances, did not demonstrate any significant difference either (*p* = 0.633). Our study failed to demonstrate any link between hormonal fluctuations and physical and psychological factors during weekly menstrual cycle monitoring. As previously suggested, FP and LP did not seem to impact physical factors, as IAT results witnessed [3]. However, unlike the previous, abovementioned study, hormonal fluctuations did not impact psychological markers, as highlighted by the Hooper questionnaire. Therefore, further studies with a larger sample size and different physical and psychological tests are required to explain how the menstrual cycles might impact performance in elite academy women soccer players.

**Funding:** This research received no external funding.

**References**

1. Martínez-Lagunas, V.; Niessen, M.; Hartmann, U. Women’s football: Player characteristics and demands of the game. *J. Sport Health Sci.* **2014**, *3*, 258–272. https://doi.org/10.1016/j.jshs.2014.10.001.
2. UEFA Women’s football across the national associations 2016/17. **2016**.
3. Mohr, M.; Krustrup, P.; Andersson, H.; Kirkendal, D.; Bangsbo, J. Match activities of elite women soccer players at different performance levels. *J. Strength Cond. Res.* **2008**, *22*, 341–349. https://doi.org/10.1519/JSC.0b013e318165fef6.
4. Manson, S.A.; Brughelli, M.; Harris, N.K. Physiological characteristics of international female soccer players. *J. Strength Cond. Res.* **2014**, *28*, 308–318. https://doi.org/10.1519/JSC.0b013e31829b56b1.

### 2.17. French Female Soccer Player’s Physiological Profile: Differences between Professional and Amateur Young Players

Tom Douchet ^1,2,^*, Nicolas Babault ^1^, Marie Sounie ^1^ and Christos Paizis ^1^

^1^ Center for Performance Expertise, CAPS, U1093 INSERM, Sport Science Faculty, University of Bourgogne-Franche-Comté, 3 allée des Stades Universitaires, BP 27877, CEDEX, 21078 Dijon, France; nicolas.babault@u-bourgogne.fr (N.B.); marie.sounie@etu.u-bourgogne.fr (M.S.); christos.paizis@u-bourgogne.fr (C.P.) 

^2^ Dijon Football Côte d’Or (DFCO), 17 rue du Stade, 21000 Dijon, France 

* Correspondence: tom.douchet@gmail.com (T.D.); Tel.: +33-3-80-39-67-01

Female participation in soccer has grown in popularity in recent years, with over 29 million players worldwide [1,2]. Speed, maximal aerobic velocity, and leg strength seem to be crucial aspects in the performance of elite women soccer players [3,4]. However, information about the influence of different practice levels on physical characteristics of female soccer players is lacking. Therefore, we assessed muscular strength, anaerobic power, and maximal aerobic running velocity of elite and amateur female French soccer players to clarify which parameters distinguish the top players from the less successful players. We tested 35 females soccer players from the French first division (elite), and amateurs division and determined body mass, height, squat jump (SJ), countermovement jump (CMJ), countermovement jump with arm swing (CMJ A), drop jump (DJ), and calf reactivity jump test. Six repetitions maximum (RM) squat, six RM hip thrust, 5 m sprint, 10 m sprint, 15 m sprint, and maximal aerobic velocity (45s–15s intermittent running field test (VMAi)) were also measured. Unpaired Student’s t tests were used to assess differences between professional and amateur young players. Statistical significance was set at *p* < 0.05. Players from a professional team were older, taller (*p* < 0.05), and had greater muscle mass than the young amateur players (*p* < 0.05). Professional players were faster in 5 m sprint, 10 m sprint, and 15 m sprint (*p* < 0.05) and had a higher maximal aerobic velocity (*p* < 0.05). Countermovement jump, countermovement jump with arm swing, calf reactivity jump test, and 6 RM squat were not different between professional, and amateur players, while squat jump, drop jump, and 6 RM hip thrust were significantly lower in amateur players and higher in the elite group (*p* < 0.05). Although performance in soccer is not determined only by measurable variables, professional players differ from amateurs in terms of rate of force development, plyometric capacity, hip extensors muscle strength, short-distance sprinting speed and maximal aerobic velocity. Coaches should emphasize the development of speed, maximal aerobic velocity, and leg strength using concentric and eccentric work in developing female soccer players.

**Funding:** This research received no external funding.

**References**

1. Martínez-Lagunas, V.; Niessen, M.; Hartmann, U. Women’s football: Player characteristics and demands of the game. *J. Sport Health Sci.* **2014**, *3*, 258–272. https://doi.org/10.1016/j.jshs.2014.10.001.
2. UEFA Women’s football across the national associations 2016/17. **2016**.
3. Mohr, M.; Krustrup, P.; Andersson, H.; Kirkendal, D.; Bangsbo, J. Match activities of elite women soccer players at different performance levels. *J Strength Cond Res* **2008.** https://doi.org/10.1519/JSC.0b013e318165fef6.
4. Manson, S.A.; Brughelli, M.; Harris, N.K. Physiological characteristics of international female soccer players. *J. Strength Cond. Res.* **2014**, *28*, 308–318. https://doi.org/10.1519/JSC.0b013e31829b56b1.

### 2.18. Effectiveness and Applicability of Resisted Sled Training in Sprint Performance: A Systematic Review with Meta-Analysis

**Javier Estellés ^1^ and Pedro E. Alcaraz ^2^**

^1^ Facultad de Deporte, UCAM Universidad Católica de Murcia, Murcia, Spain; cjestelles@alu.ucam.edu (J.E.) 

^2^ UCAM Research Center for High Performance Sport, UCAM Universidad Católica de Murcia, Murcia, Spain; palcaraz@ucam.edu (P.E.A.)

Sprinting is one of the most important skills associated with sports performance [1]. One of the resisted sprint methods associated with the development of this skill is resisted sled training (RST), which appears to be effective for the development of sprinting, especially for the acceleration phase [2]. However, these adaptations may depend on variables such as intensity, volume, and duration of the program [2]. The main objective of this review was to analyze the current state of the literature on RST and its effects on sprint performance in both the acceleration and maximal velocity phases. On the other hand, an attempt has been made to determine which RST load characteristics produce the greatest improvements in sprint performance. A literature search was performed in the major databases (PubMed, SPORT Discus and Web of Science) to identify all articles published up to 18 May 2022, that relate RST training to sprint performance. A total of 505 articles were found. After applying the inclusion criteria, a total of 21 articles were finally included in this meta-analysis. Considering that the standardized mean difference (SMD) was used as an outcome measurement, significant improvements were observed between baseline and post-training in full sprint performance (SMD = −0.36) and acceleration phase (SMD = −0.49). However, these improvements were not observed in the maximum velocity phase (SMD = −0.19). No significant improvements were found when comparing the results of the RST and control groups in any of the phases analyzed. In terms of loading conditions, similar significant improvements were observed when using loads of <20%BM (SMD = −0.46) and ≥20% BM (SMD = −0.51) in the acceleration phase and full sprint (SMD = −0.36). In addition, greater adaptations were observed when using high volumes in the acceleration phase (SMD = −0.65) and full sprint (SMD = −0.49). In relation to the sex of participants, greater results were found in male’s groups in the acceleration phase (SMD = −0.54). Based on these results, it appears that RST produces greater improvements in sprinting in male athletes, especially in the acceleration phase. Both high (≥20%BM) and low (<20%BM) sled loads produce similar improvements in sprint performance. Finally, high volumes (>2.680 m) were shown to be more effective for sprint improvement.

**Funding:** This research received no external funding.

**References**

1. Haugen, T.; Tønnessen, E.; Hisdal, J.; Seiler, S. The role and development of sprinting speed in soccer. *Int. J. Sports Physiol. Perform.* **2014**, *9*, 432–441. https://doi.org/10.1007/s40279-018-0947-8.
2. Alcaraz, P.E.; Carlos-Vivas, J.; Oponjuru, B.O.; Martínez-Rodríguez, A. The effectiveness of resisted sled training (RST) for sprint performance: A systematic review and meta-analysis. *Sports Med.* **2018**, *48*, 2143–2165. https://doi.org/10.1007/s40279- 018-0947-8.

### 2.19. Pre-Competitive Period: Tapering Technique for Performance Optimization a Literature Review

**Ygor Fajardo**

Independent Researcher; ygorf2301@gmail.com (Y.F.)

This study aimed to demonstrate one of the various formats of techniques used by professionals in the physical preparation of athletes in the pre-competition phase. At the end of a long training journey, athletes prepare for a target competition, where coaches seek the best way to make them increase their physical performance to improve technical movements. In the final weeks of training, coaches use specific strategies with the goal that athletes achieve the best physical and technical results in the sport for which they compete. There are many strategies and protocols that can be used successfully in this phase of training [1]. The purpose of this review was to present current evidence on the use of tapering, monitoring, and key exercise methods [2]. Tapering is a strategy used by coaches on their athletes to optimize performance in maintenance and adaptation induced by training by reducing variables such as volume, to ultimately develop the physiological demands in the pre-competitive period. Using “optimal power load” models and monitoring through devices, it is possible to determine loads and exercises of training sessions for better neuromuscular adaptive inductions [3]. The ideal load is determined through the speed of movement that the athlete performs in the exercise, and the use of this tool aims to increase the power of a given exercise. Through the movements of the vertical jump, half squat, and squat jump, it is possible to determine and measure the speed and power generated by the athlete, making it possible to verify how fast he/she is and if there was any increase or decrease in his/her performance in order to manipulate and then adjust the training loads. In certain exercises, such as the squat jump, individuals who show greater force production by moving the bar with more speed tend to develop greater force production capacity when they are in their technical training routine [3]. Using the monitoring and tapering devices for athletes in the pre-competitive period, the chances of increased performance are low to moderate, which can define the final result in a competition.

**Funding:** This research received no external funding.

**References**

1. Bosquet, L.; Montpetit, J.; Arvisais, D.; Mujika I. Effects of tapering on performance: a meta-analysis. *Med. Sci. Sports Exerc.* **2007**, *39*, 1358–1365. https://doi.org/10.1249/mss.0b013e31806010e0.
2. Jiménez-Reyes, P.; Castaño-Zambudio, A.; Cuadrado-Peñafiel, V.; González-Hernández, J.M.; Capelo-Ramírez, F.; Martínez-Aranda, L.M.; González-Badillo, J.J. Differences between adjusted vs. non-adjusted loads in velocity-based training: consequences for strength training control and programming. *PeerJ* **2021**, *9*, e10942. https://doi.org/10.7717/peerj.10942.
3. Loturco, I.; Dello Iacono, A.; Nakamura, F.Y.; Freitas, T.T.; Boullosa, D.; Valenzuela, P.L.; Pereira, L.A.; McGuigan, M.R. The Optimum Power Load: A Simple and Powerful Tool for Testing and Training. *Int. J. Sports Physiol. Perform.* **2022**, *17*, 151–159. https://doi.org/10.1123/ijspp.2021-0288.

### 2.20. Seasonal Variations in the Neuromuscular Performance in Professional Soccer Players

**Ricardo Ferreira ^1,2^, Regiane Albertini Carvalho ^1^ and Rodolfo de Paula Vieira ^2^**

^1^ Universidade Federal de São Paulo; ricardocalves@hotmail.com (R.F.); regiane.albertini@unifesp.br (R.A.C.) 

^2^ Universidade São Lucas; rodrelena@yahoo.com.br (R.P.V.)

To reach a superior performance in a complex sport such as football, a series of physical abilities are required such as aerobic endurance, sprinting speed, and strength [1]. That complexity is related to the unpredictable environment of the game such as players’ interactions, tactical and technical influences, and climate conditions, among others. Neuromuscular strength is one of the most important physical abilities for professional soccer players and several training methods have been implemented to improve this capacity during distinct phases of the season [2,3]. More recently, the optimum power load (OPL) has been used as a practical and effective alternative from the traditional strength methods to improve speed and power performance in elite soccer players [1,3]. This method consists of moving a moderate load with a maximum speed, which could be of great advantage against the more “traditional” methods, due to its simplicity and ease to implement in practical scenarios [2,3]. The aim of this study was to investigate the neuromuscular variations of professional football players submitted to 3 months of training comprising high volumes of soccer-specific sessions (six sessions per week) and strength/power training based on the OPL, performed once in a week. Thirty professional soccer players (body mass = 68.36 ± 9.31 kg; height = 165.11 ± 4.75 cm; age = 22.80 ± 2.01 years) from a “B league” in Brazil were involved in this study. The countermovement jump (CMJ) was assessed in three different occasions: pre-season (T1), pre-competitive (T2) and in the competitive phase (T3), with an interval of 45 days between the tests. An ANOVA with repeated measures was used to compare the CMJ in the three different assessments, and the level of significance was set as *p* < 0.05. The jump height was higher in the pre-competitive period and in the competitive period when compared to a pre-season period, and in the competitive period the CMJ performance was maintained when compared to the pre-competitive (T1: 36.65 ± 4.08 cm; T2: 42.89 ± 4.91; T3: 41.72 ± 4.00 cm; *p* < 0.05). Our study shows that a training strategy based on the OPL can be a practical and safe method to improve the vertical jumping ability in periods of high soccer-specific training volumes.

**Funding:** This research received no external funding.

**References**

1. Turner, A.N.; Stewart, P.F. Strength and Conditioning for Soccer Players. *Strength Cond. J.* **2014**, *36*, 1–13. https://doi.org/10.1519/SSC.0000000000000054.
2. Loturco, I.; Nakamura, F.Y.; Tricoli, V.; Kobal, R.; Cal Abad, C.C.; Kitamura, K. et al. Determining the Optimum Power Load in Jump Squat Using the Mean Propulsive Velocity. *PLoS ONE* **2015**, *10*, e0140102. https://doi.org/10.1371/journal.pone.0140102.
3. Loturco, I.; Pereira, L.A.; Reis, V.; Bishop, C.; Zanetti, V.; Alcaraz, P.E.; Freitas, T.T.; Mcguigan, M.R. Power training in elite young soccer players: Effects of using loads above or below the optimum power zone. *J Sports Sci* **2020**, *38*, 1416–1422. https://doi.org/10.1080/02640414.2019.1651614.

### 2.21. Can Weight Loss Affect Muscle Power Performance on Taekwondo Competition Day?

**Thais Frois, Larissa Faria, José Geraldo, Amanda Ribeiro and Maicon Albuquerque**

Sports Laboratory, Department of Sports, Federal University of Minas Gerais, Brazil; thaisfroisefisica@gmail.com (T.F.); lof.ufv@gmail.com (L.F.); josegeraldo100@gmail.com (J.G.); aribeiroufmg@gmail.com (A.R.); lin.maicon@gmail.com (M.A.) 

The control of body mass is an aspect of fundamental importance for Taekwondo. In addition, a common practice in this modality is weight loss in order to gain a competitive advantage [1]. However, this practice can compromise health and performance [2]. Regarding performance, muscular power of the lower limbs is a determining component for scoring points during the fight, and if compromised by the practice of weight loss, it can generate a loss in performance during the competition [3]. Muscle power can be indirectly and easily measured on the day of the fight by countermovement jump (CMJ). Therefore, it seems to be important to verify if weight loss affects muscular power performance of taekwondo athletes in a competition [3]. So, the aim of the present study was to verify whether weight loss affects the neuromuscular performance on the competition day of taekwondo athletes. Ten Taekwondo athletes (x = 17.4 ± 3.29) participated in the study, in which the height of the CMJ was evaluated using a contact mat and body mass assessed using a digital scale at three different times: before the competition (day 1), on the day of the official weighing (day 2) and on the day of the competition (day 3). Data analysis was performed using one-way ANOVA, Effect Size (eta-square) and Bonferroni’s Post Hoc. The results showed that there was a significant difference in CMJ F(11.64; df = 2.18; *p* < 0.01; η^2^ = 0.56), and in body mass between the three moments F(10.44; df = 2.18; *p* < 0.01; η^2^ = 0.53). Post hoc indicated that the height of the CMJ decreased at the moment of competition and weighing when compared to the moment before the competition (*p* < 0.01). Body mass increased at the moment of competition compared to the moment before the competition (*p* < 0.01) and reduced at the moment of weighing when compared to the moment before the competition (*p* < 0.01). The results indicate that on the day of the competition, the athletes showed worsening muscle power when compared to the moment before the competition. It is also noteworthy that several factors may have affected performance, such as weight loss, travel, and sleep quality, among others [1,4]. In conclusion, muscle power on competition day was affected by weight loss, but other factors may contribute to this result.

**Funding:** This study was financed by the Emenda Parlamentar awarded by the Deputado Federal Luis Tibé (no.: 27620007). Also, this study was financed in part by Coordenação de Aperfeiçoamento de Pessoal de Nível Superior -Brasil (CAPES) -Finance Code 001.

**References**

1. Silva Santos, J.F.; Takito, M.Y.; Artioli, G.G.; Franchini, E. Weight loss practices in Taekwondo athletes of different competitive levels. *J. Exerc. Rehabil.* **2016**, *12*, 202–208. https://doi.org/10.12965/jer.1632610.305.
2. Artioli, G.G.; Saunders, B.; Iglesias, R.T.; Franchini, E. It is Time to Ban Rapid Weight Loss from Combat Sports. *Sports Med.* **2016**, *46*, 1579–1584. https://doi.org/10.1007/s40279-016-0541-x.
3. Albuquerque, M.R.; Tavares, L.D.; Longo, A.R.; Caldeira Mesquita, P.H.; Franchini, E. Relationship between Indirect Measures of Aerobic and Muscle Power with Frequency Speed of Kick Test Multiple Performance in Taekwondo Athletes. *Int. J. Sports Med.* **2022**, *43*, 254–261. https://doi.org/10.1055/a-1546-9221.
4. Garthe, I.; Raastad, T.; Refsnes, P.E.; Koivisto, A.; Sundgot-Borgen, J. Effect of two different weight-loss rates on body composition and strength and power-related performance in elite athletes. *Int. J. Sport Nutr. Exerc. Metab.* **2011**, *21*, 97–104. https://doi.org/10.1123/ijsnem.21.2.97.

### 2.22. Viability of a Deep Learning Algorithm for Pose Estimation and Tracking of Basketball Athletes during an Official Match

**Bruno Giovanini and Felipe Arruda Moura**

Laboratory of Applied Biomechanics, Londrina State University, Londrina, Brazil; bruno.giovanini@uel.br (B.G.) 

Athlete tracking is considered paramount nowadays, and most methods involve global- or local-positioning systems where some limitations are present—such as a high cost, device wearing rules or ability to function in enclosed facilities [1]. Optical tracking overcomes these issues, being equally valid and reliable but implying time consuming approaches with manual intervention [2]. To handle limitations of conventional optical tracking and potentialize this method, we propose a novel method using pose estimation algorithms. This process involves three components that require attention: pose estimation (key-point detection), assembly (grouping key-points to distinct athletes) and tracking along a video. Thus, this study aimed to examine the viability of a deep learning algorithm for pose estimation and tracking of basketball athletes during an official match. We used the DeepLabCut—a tool combining pre-trained deep neural networks and customized feature detection to track key-points in videos [3]. First, we trained a model using 50 frames from a video of an official match of basketball. On these frames, we labeled three key-points of each athlete: left foot, right foot, and sternum. We trained the model for 200,000 iterations. The trained model was tested on a video with 10,366 frames (30 Hz) of the same official match used to train the model. To evaluate pose estimation, we calculated the root mean square error (RMSE) of labeled and estimated key-points (in pixels) in the testing dataset. Quality of assemblies was assessed by the percentage of manual and automatic tracking achieved for each player in a sample of 5000 frames. To potentially improve our model, 50 outlier frames (with low-confidence estimations) were extracted, relabeled correctly, and the model was retrained. This process was repeated until 200 frames were labeled. Overall RMSE of the testing dataset was 5.40, 4.87, 4.96, and 4.47 pixels using a model with, 50, 100, 150, and 200 labeled frames, respectively. Automatic tracking was successfully achieved in 80.8% (min.: 71.5%; max.: 84.4%), 80.2% (min.: 69.7%; max.: 86.1%), 81.6% (min.: 72.8%; max.: 88.9%), and 83.1% (min.: 73.1%; max.: 91.1%) of the analyzed frames using a model with, 50, 100, 150, and 200 labeled frames, respectively. Pose estimation and assembly using the DeepLabCut presented promising results that could represent a viable and low-cost tool to successfully track athletes during training or competition.

**Funding:** This research was funded by Conselho Nacional de Desenvolvimento Científico e Tecnológico, grant number 401004/2022-8.

**References**

1. Torres-Ronda, L.; Beanland, E.; Whitehead, S.; Sweeting, A.; Clubb, J. Tracking systems in team sports: a narrative review of applications of the data and sport specific analysis. *Sport. Med.–Open* **2022**, *8*, 15. https://doi.org/10.1186/s40798-022-00408-z.
2. Linke, D.; Link, D.; Lames, M. Validation of electronic performance and tracking systems EPTS under field conditions. *PLoS ONE* **2018**, *13*, e0199519. https://doi.org/10.1371/journal.pone.0199519.
3. Lauer, J.; Zhou, M.; Ye, S.; Menegas, W.; Schneider, S. et al. Multi-animal pose estimation, identification and tracking with DeepLabCut. *Nat. Methods* **2022**, *19*, 496–504. https://doi.org/10.1038/s41592-022-01443-0.

### 2.23. Differences and Associations between Strength Deficit and Selected Performance Variables in Highly Trained Female Volleyball Players

Julián Giráldez ^1,2^, Javier Gálvez González ^2^ and Santiago Zabaloy ^1,^*

^1^ University of Flores, Faculty of Phyisical Activity and Sports, Buenos Aires, Argentina; julgiraldez@gmail.com (J.G.)

^2^ Pablo de Olavide University, Faculty of Sports Science; jgalgon@upo.es (J.G.G.)

* Correspondence: santiagozabaloy@hotmail.com (S.Z.) 

The strength deficit (SDef) is an important indicator of athletic performance [1,2], and it was previously reported to be able to discriminate between fast and slow athletes [1]. For example, SDef was lower when sprinters were compared to elite rugby players [1] and amateur rugby backs also demonstrated a lower SDef versus forwards [2]. Nonetheless, more information is needed with regard to SDef differences and associations to some selected measures of performance in highly trained female volleyball athletes. Therefore, this study aimed to (i) compare the differences in SDef between female volleyball players of different performance levels (elite vs. sub-elite); and (ii) investigate the relationships between SDef and various performance variables. Twenty-six elite female volleyball players representing a top club in Argentina were divided into two groups according to their competitive level (elite [n = 13; age, 21.77 ± 4.13 years; height, 176.84 ± 7.63 cm; body mass, 74.25 ± 9.97 kg] and sub-elite [n = 13; age, 19.08 ± 2.02 years; height, 173.15 ± 4.74 cm; body mass, 68.78 ± 5.05 kg]). Participants completed the following tests: countermovement jump (CMJ), repeated jumps (RJT 10/5), loaded CMJ (load needed to attain a 20-cm height [CMJ20]) and an incremental loading test in the Squat exercise to determine the 1 repetition maximum (1-RM SQ, absolute and relative to body mass [BM]), SDef (from 40% to 90% 1RM) and other force-derived variables (i.e., maximum SQ mean propulsive power (MPP), MPP load, Peak Force). An independent Student’s *t*-test was used to examine differences between elite and sub-elite across all variables. Pearson’s correlation coefficient was used to evaluate the relationships between SDef and the selected performance variables. No significant differences were observed between groups for any of the selected measures including SDef (*p* > 0.05, ES = trivial to moderate). In addition, significant associations were found between MPP relative to BM and SDef at 80% and 90% 1-RM (r = −0.423 to −0.408). Furthermore, CMJ was significantly related to 1-RM SQ (r = 0.481), MPP load (r = 0.449), MPP relative (r = 0.619) and CMJ20 (r = 0.779). In female volleyball players, SDef is not capable of discriminating between competitive levels. However, optimum power loads may be a suitable option to reduce SDef values at high loads (80% and 90%). Additionally, training programs should focus on targeting the optimal power zone in the squat in order to improve jumping ability.

**Funding:** This research received no external funding.

**References**

1. Loturco, I.; Pereira, L.A.; Freitas, T.T.; Bishop, C.; Pareja-Blanco, F.; McGuigan, M.R. Maximum strength, relative strength, and strength deficit: Relationships with performance and differences between elite sprinters and professional rugby union players. *Int. J. Sports Physiol. Perform.* **2021**, *16*, 1148–1153. https://doi.org/10.1123/ijspp.2020-0342.
2. Zabaloy, S.; Giráldez, J.; Fink, B.; Alcaraz, P.E.; Pereira, L.A.; Freitas, T.T.; Loturco, I. Strength deficit in elite young rugby players: differences between playing positions and associations with sprint and jump performance. *J. Strength Cond. Res.* **2022**, *36*, 920–926. https://doi.org/10.1519/JSC.0000000000004234.

### 2.24. Effects of Including Different Combinations of Optimum Power Load Training in Military Physical Training on Maximal Strength and Muscle Damage Markers

**Michel Gonçalves ^1,2^, Irineu Loturco ^3^, Paula Ferreira ^4^, Marcio Antonio Sena ^4^, Marly Zanetti ^4^ and Humberto Miranda ^2^**

^1^ Brazilian Army of Physical Training Center; michel_fitness@hotmail.com (M.G.) 

^2^ Performance, Training, and Physical Exercise Laboratory, Federal University of Rio de Janeiro; humbertomirandaufrj@gmail.com (H.M.)

^3^ Nucleus of High Performance in Sport; São Paulo; irineu.loturco@terra.com.br (I.L.) 

^4^ Brazilian Army Research Institute of Phyisical Fitness; paulafferr89@gmail.com (P.F.); mabsmarcio@gmail.com (M.A.S.); marly_zanetti@hotmail.com (M.Z.)

This study examined the effects of the inclusion of resistance training (RT) in military physical training (MPT) on maximal strength (MS) and muscle damage markers (MDM). Twenty-six healthy young adults, Brazilian Army military personnel, were randomized into three groups, as follows: G1 (started with light-load and then progressed to heavy-load), G2 (started with heavy-load and then progressed to light-load) and CON (performed traditional military physical training, namely running and calisthenics). Each group performed RT three times per week for 8 weeks with a similar training volume. Training intensity was defined according to the load that maximized power output (i.e., optimum power load; OPL) (i.e., light-load = OPL × 0.8; heavy-load = OPL × 1.2) [1]. They were evaluated in MS and MDM in three different moments: PRE, INT, and POST. MS increase similarly within the groups (G1: PRE-130.63 ± 23.59 Kg, INT-150.50 ± 26.52 Kg, POST-168.75 ± 30.44 Kg, *p* = 0.046; *p* < 0.001; G2: PRE-109.75 ± 12.89 Kg, INT-125.63 ± 18.41 Kg, POST-135.63 ± 20.60 Kg; *p* = 0.018; *p* < 0.001). No significant gains were found in MS in CON (*p* = 0.558). There were no increases in MDM in all groups in any time-point (G1: LDH *p* = 0.657; TGO *p* = 0.358; TGP *p* = 0.150; G2: LDH *p* = 0.135; TGO *p* = 0.197; TGP *p* = 0.140; CON:LDH *p* = 0.273; TGO *p* = 0.073; TGP *p* = 0.358). In conclusion, 8 weeks of RT with different combinations of OPL training (below/above the OPL) included in MPT produced significant gains in maximal strength, without accumulating muscle damage throughout training in military young adults.

**Funding:** This research received no external funding.

**Reference**

1. Loturco, I.; Pereira, L.A.; Reis, V.P.; Bishop, C.; Zanetti, V.; Alcaraz, P.E.; Freitas, T.T.; Mcguigan, M.R. Power Training in Elite Young Soccer Players: Effects of Using Loads above or below the Optimum Power Zone. *J. Sports Sci.* **2019**, 38, 1416–1422. https://doi.org/10.1080/02640414.2019.1651614.

### 2.25. Effects of Pitch Size and Scoring Methods during Small-Sided Games on Mechanical Load in Brazilian Professional Soccer Players

**Luiz Guilherme Gonçalves ^1,2^, Raul Victor Fernandes da Costa ^2^, Eduardo Rostaiser ^3^, Alejandro Pastor ^3^, Bruno Pasquarelli ^3^ and Rodrigo Aquino ^2^**

^1^ Department of Physiology, Association Ferroviárica of Sports, Araraquara, SP, Brazil; goncalves.lgui@gmail.com (L.G.G.) 

^2^ LabSport, Post-graduate Program in Physical Education, Centre of Physical Education and Sport (CEFD), Federal University of Espírito Santo, Vitória, ES, Brazil; raulvfcosta@gmail.com (R.V.F.C.); aquino.rlq@gmail.com (R.A.)

^3^ Catapult Group International Ltd., Melbourne, Australia; eduardo.rostaiser@catapultsports.com (E.R.); alejandro.pastor@catapultsports.com (A.P.); bruno.pasquarelli@catapultsports.com (B.P.) 

The use of small-sided games (SSGs) seems to be an effective practice for optimizing technical skills, tactical behaviors, and, consequently, physical fitness in soccer training [1]. A previous umbrella review showed that pitch size and scoring methods influence the external load parameters during SSGs [2]. However, the mechanical load obtained by new metrics of inertial movement analyses (e.g., Football Movement Profile—FMP, Catapult Sports metric based on linear acceleration and angular velocity data) was scarcely investigated in previous studies. Therefore, this study aimed to investigate the effects of pitch size and scoring methods on mechanical load in Brazilian professional soccer players (n = 7, (23.1 ± 4.0 y). The SSGs conditions were 4 vs. 4 (possession game, PosG; progression game, PG scoring in endzones) and GK + 4 vs. 4 + GK (regular game; RG). The three types were performed in small (SSG_S: 32 × 22 m; area per player = 58.6 m^2^) and large pitch size (SSG_L: 45 × 35 m; area per player = 131.2 m^2^). All the conditions had 3 × 4 min; 3 min passive rest, giving a total of nine SSG bouts. The mechanical load parameters were collected using inertial movement sensors (Catapult Vector S7). The ANOVA two-way (*p* < 0.05) was used to compare the mechanical load parameters according to the SSGs constraints (pitch size and scoring methods). In small pitch, FMP in medium dynamic duration (%) presented greater values in ball possession games than progression without GK (*p* = 0.001; ES = 2.86, very large) and progression with GK (*p* = 0.004; ES = 2.60, very large). In addition, ball possession configuration showed high values of FMP medium running when compared to progression without GK in large pitch (*p* = 0.04; ES = 1.90, very large). Players showed lower FMP medium dynamic in large than small pitch in ball possession configuration (*p* < 0.005; ES = 2.12, very large). Moreover, there were differences between ball possession and progression with GK configurations in player load, number of repeated high-intensity efforts (RHIE) and FMP medium running duration (*p* < 0.001–0.005; ES = 1.05–2.26, very large). In general, the possession game in larger pitch size was the most intense in mechanical load. Therefore, coaches and practitioners can implement these task constraints to increase the mechanical load in professional soccer players during SSGs session training. The micro-technology metrics driven by inertial movement sensors represent an important tool for coaches’ daily training prescription.

**Funding:** This research received no external funding.

**References**

1. Lacome, M.; Simpson, B.M.; Cholley, Y.; Lambert, P.; Buchheit, M. Small-Sided Games in Elite Soccer: Does One Size Fit All? *Int. J. Sports Physiol. Perform.* **2018**, *13*, 568–576. https://doi.org/10.1123/ijspp.2017-0214.
2. Clemente, F.M.; Afonso, J.; Sarmento, H. Small-Sided Games: An Umbrella Review of Systematic Reviews and Meta-Analyses. *PLoS ONE* **2021**, *Ahead*-*of*-*p*.

### 2.26. Does the Change of Head Coach Affect the Running Performance According to the Different Phases of Soccer Match-Play?

**Luiz Guilherme Gonçalves ^1,2^, Raul Victor Fernandes da Costa ^2^, Maxwell Viana Moraes-Neto ^2^, Eduardo Rostaiser ^3^, Alejandro Pastor ^3^, Bruno Pasquarelli ^3^ and Rodrigo Aquino ^2^**

^1^ Department of Physiology, Association Ferroviária of Sports, Araraquara, SP, Brazil; goncalves.lgui@gmail.com (L.G.G.) 

^2^ LabSport, Post-graduate Program in Physical Education, Centre of Physical Education and Sport (CEFD), Federal University of Espírito Santo, Vitória, ES, Brazil; raulvfcosta@gmail.com (R.V.F.C.); netomax03@gmail.com (M.V.M.N.); aquino.rlq@gmail.com (R.A.)

^3^ Catapult Group International Ltd., Melbourne, Australia; eduardo.rostaiser@catapultsports.com (E.R.); alejandro.pastor@catapultsports.com (A.P.); bruno.pasquarelli@catapultsports.com (B.P.) 

Coach replacement can influence the external load of soccer match-play [1]. However, match analysis of running performance according to different phases of the match (e.g., organization offensive/defensive; transition offensive/defensive) would provide a more holistic approach to understanding running performance [2]. Therefore, this study aimed to investigate the effects of a change of head coach on running performance according to different phases of match-play in Brazilian professional soccer players (n = 24; 26.2 ± 5.6 y). The players who participated ≥ 60 min of the match time were considered as inclusion criteria. A timeline of the match’s duration was generated by SBG Sports Software to define ball possession, no-ball possession, organization offensive/defensive, and transition offensive/defensive. The distance- and accelerometry-based measures were recorded during 14 matches using a global position system (Catapult Vector S7): (i) total distance covered relative per minute (TDrel; m/min); (ii) total distance covered in moderate-speed running relative per minute (MSRrel: 14.3–19.7 km·h^−1^; m/min); (iii) total distance covered in high-speed running relative per minute (HSRrel: 19.8–25.1 km·h^−1^; m/min); (iv) total distance in sprint running relative per minute (SRrel: ≥25.2 km·h^−1^; m/min); (v) relative distance covered in high-acceleration per minute played (ACCrel: >2 m·s^2^; m/min); (vi) relative distance covered in high-deceleration per minute played (DECrel < −2 m·s^2^; m/min); (vii) Player Load relative per minute (PLrel; a.u./min). Head coach was considered coach #1 (n = 6 matches; mean idea based on the indirect style of play) and coach #2 (n = 8 matches; mean idea based on the direct style of play). Separate linear mixed models were performed to compare (fixed effects) change coach, with “player ID” included as a random effect. A significance level of *p* < 0.05 was adopted. Coach #1 presented greater values of TDrel, MSRrel, HSRrel, SRrel, ACCrel, DECrel and, PLrel compared to coach #2 during no-ball possession phase (*t* = 2.247–5.824, *p* < 0.001–0.041, ES = large-very large). In defensive transition phase, coach #1 showed higher TDrel, MSRrel, HSRrel, ACCrel and, PLrel (*t* = 2.626–4.412, *p* < 0.001–0.013, ES = 0.25–0.66, small-large) than coach #2. In defensive organization, coach #1 revealed greater TDrel compared to head coach #2 (*t* = 2.683, *p* = 0.021, ES = 0.62, large). Overall, the indirect style of play (coach #1) presented higher running demands in organization/transition defensive phases than the direct style of play (coach #2). Coaches and practitioners can design more specific physical training to better prepare players for different phases of match-play.

**Funding:** This research received no external funding.

**References**

1. Augusto, D.; Brito, J.; Aquino, R.; Figueiredo, P.; Eiras, F.; Tannure, M.; Veiga, B.; Vasconcellos, F. Contextual Variables Affect Running Performance in Professional Soccer Players: A Brief Report. *Front. Sport Act. Living* **2021**, *3*, 778813. https://doi.org/10.3389/fspor.2021.778813.
2. Aquino, R.; Machado, J.C.; Clemente, F.M.; Praca, G.M.; Goncalves, L.G.C.; Melli-Neto, B.; Ferrari, J.V.S.; Palucci Vieira, L.H.; Puggina, E.F.; Carling, C. Comparisons of ball possession, match running performance, player prominence and team network properties according to match outcome and playing formation during the 2018 FIFA World Cup. *Int. J. Perform. Anal. Sport* **2019**, *19*, 1026–1037. https://doi.org/10.1080/24748668.2019.1689753.

### 2.27. Speed-Related Abilities Are Similarly Improved after Sled Training under Different Magnitudes of Velocity Loss in Professional Soccer Players

Rafael Grazioli ^1^, Irineu Loturco ^2,3,4^, Filipe Veeck ^1^, Igor Setuain ^5^, Martinho Inácio ^1^, Ronei S. Pinto ^1^ and Eduardo L. Cadore ^1,^*

^1^ Exercise Research Laboratory, School of Physical Education, Physiotherapy, and Dance, Universidade Federal do Rio Grande do Sul, Porto Alegre, RS, Brazil; rafael_grazioli@hotmail.com (R.G.)

^2^ NAR—Nucleus of High Performance in Sport, São Paulo, Brazil; irineu.loturco@terra.com.br (I.L.)

^3^ Department of Human Movement Sciences, Federal University of São Paulo, São Paulo, Brazil

^4^ University of South Wales, Pontypridd, Wales, United Kingdom

^5^ Department of Health Sciences, Public University of Navarra, Tudela, Navarra, Spain; isetuain@tdnclinica.es (I.S.)

* Correspondence: edcadore@yahoo.com.br (E.L.C.) 

Resisted sprint training (RST) with sled towing has been commonly used by practitioners in soccer and other team sports, which has led to an increased number of studies on this topic [1,2]. Nevertheless, there are important controversies regarding the optimal magnitude of the sled loads to use during sled training programs [1,3,4]. The present study examined and compared the effects of two 8-week RST interventions using different sled loads on the neuromuscular performance of soccer players. Twenty-one elite soccer players (age 25.9 ± 5.4 y) completed the study. Athletes were randomly allocated to two groups who trained under distinct magnitudes of velocity loss (VL) and, hence, loading conditions: (1) “moderate-load group”-sled loads that induced 15% VL relative to unloaded sprints MLG, n = 11); and (2) “heavy-load group”—sled loads that induced 40% VL relative to unloaded sprints (HLG, n = 10). Linear sprint (10 m), change of direction (COD) speed, curve sprint (CS), resisted sprint (at 15% and 40% VL), vertical jump, hamstring eccentric strength, and intermittent cardiorespiratory fitness (Yo-Yo IR1) tests were conducted pre- and post-training. A two-way repeated measures analysis of variance revealed a time effect for decreases in 10 m sprint, COD speed, and CS times (*p* = 0.003, *p* = 0.05, *p* = 0.004, respectively). Furthermore, both 15% VL and 40% VL resisted sprint times decreased significantly at post-training (*p* = 0.036 and *p* = 0.019, respectively). Moreover, there was a time effect for increases in intermittent cardiorespiratory fitness (*p* = 0.002). Lastly, soccer players presented significant decreases in hamstring eccentric strength after the 8-week period (*p* = 0.017). The remaining variables did not change significantly (*p* > 0.05). Importantly, there was no time vs. group interaction for any outcome (*p* > 0.05). In conclusion, both moderate (15% VL) or heavy sled loads (40% VL) improved 10 m sprint, CS, and COD speed performances after 8 weeks of RST in elite soccer players.

**Funding:** This research received no external funding.

**References**

1. Zabaloy, S.; Freitas, T.T.; Pareja-Blanco, F.; Alcaraz, P.E.; Loturco I. Narrative review on the use of sled training to improve sprint performance in team sport athletes. *Strength Cond. J.* **2022**, *45*, 13–28. https://doi.org/10.1519/SSC.0000000000000730.
2. Grazioli, R.; Loturco, I.; Lopez, P.; Setuain, I.; Goulart, J.; Veeck, F.; Inácio, M.; Izquierdo, M.; Pinto, R.S.; Cadore, E.L. Effects of moderate-to-heavy sled training using different magnitudes of velocity loss in professional soccer players. *J. Strength Cond. Res.* **2020**, *37*, 629–635. https://doi.org/10.1519/JSC.0000000000003813.
3. Petrakos, G.; Morin, J.B.; Egan, B. Resisted sled sprint training to improve sprint performance: a systematic review. *Sports Med.* **2016**, *46*, 381–400. https://doi.org/10.1007/s40279-015-0422-8.
4. Alcaraz, P.E.; Carlos-Vivas, J.; Oponjuru, B.O.; Martínez-Rodríguez, A. The effectiveness of resisted sled training (RST) for sprint performance: a systematic review and meta-analysis. *Sports Med.* **2018**, *48*, 2143–2165. https://doi.org/10.1007/s40279-018-0947-8.

### 2.28. Effect of Vitamin D and Calcium on Falls and Fractures in the Home Population: A Review Systematic and Meta-Analysis

**Victor Jorge Guerreiro ^1^, Lucas de Souza Campos Santos ^2^ and Pablo Christiano Bardosa Lollo ^1^**

^1^ Programa de Pós-Graduação, Faculdade de Ciências da Saúde, Universidade Federal da Grande Dourados (UFGD); victorjorgeguerreiro@hotmail.com (V.J.G.); pablo.christiano@gmail.com (P.C.B.L.) 

^2^ Faculdade de Ciências da Saúde, Universidade Federal da Grande Dourados (UFGD); lucasouzacampoz@hotmail.com (L.S.C.S.)

Advancing age directly impairs bone functions, promoting an imbalance between remodeling and absorption. In the elderly, this process can result in an increased risk of falls and bone fractures. Calcium and vitamin D supplementation, generally in a co-ingestion model, has been one of the most used interventions to mitigate the risk of falls and fractures [1]. Substances have a direct role in bone physiology and can be implemented in a practical and inexpensive way. However, there is still no academic consensus on the real effectiveness of this type of supplementation in the risk of fractures and falls [2]. The main objective of this systematic review was to verify the effect of vitamin D or vitamin D in combination with calcium on falls and fractures in the elderly. A systematic review was performed following the PRISMA protocol, using the following databases: PubMed, Web of Sciences, CENTRAL and Scopus. Randomized controlled trials in older adults (65 years and older; postmenopausal women were considered due to bone risk similar to older adults) with intervention of vitamin D or vitamin D plus calcium (no other substances) and placebo, with no intervention or control group of calcium included. The risk of bias was assessed by RoB 2.0. The hazard ratio (RR) was calculated in each intervention group to summarize the results into a single effect measure using the Mantel–Haenszel method for the fixed effects model and the DerSimonian and Laird method for the random effects model. The database search returned 4180 studies, of which 28 met all eligibility criteria and were included in the final analysis. Of the 28 studies, 27 were blindly controlled. Sample sizes analyzed ranged from 64 to 5292 participants and intervention/follow-up intervals ranged from 4 to 208 weeks. The results showed that supplementation with vitamin D alone or vitamin D plus calcium has distinct effects on fractures and falls. The presence/absence of corresponding blood calcium levels can largely influence the results of the vitamin D intervention. Compared to placebo or no treatment, vitamin D itself showed no protective effect on fractures or falls. However, compared to placebo or no treatment, vitamin D plus calcium demonstrated a protective effect on total, non-vertebral and hip fractures. In conclusion, this meta-analysis showed that supplementing older adults with vitamin D had no protective effect. However, co-intervention with vitamin D plus calcium plays a protective role in fractures and possibly falls in the elderly.

**Funding:** This research received no external funding.

**References**

1. Anagnostis, P.; Bosdou, J.K.; Kenanidis, E.; Potoupnis, M.; Tsiridis, E.; Goulis, D.G. Vitamin D supplementation and fracture risk: Evidence for a U-shaped effect. *Maturitas* **2020**, *141*, 63–70. https://doi.org/10.1016/j.maturitas.2020.06.016.
2. Benini, C.; Esposito, D. Adami, G.; Vantaggiato, E.; Gatti, D.; Rossini, M.; Fassio, A. Calcium and vitamin D supplementation: when and why. *Minerva Obstet. Gynecol.* **2021**, *73*, 704–713. https://doi.org/10.23736/S2724-606X.20.04682-1.

### 2.29. Acute Effects of Different Complex-Contrast Protocols on Neuromuscular Performance in Resistance-Trained Individuals

Daniel Gutiérrez-Flores ^1,2^, Antonio Martínez-Serrano ^1,4^, Pedro E. Alcaraz ^1,4^ and Tomás T. Freitas ^1,3,4,5,6,^*

^1^ UCAM Research Center for High Performance Sport, UCAM Universidad Católica de Murcia, Murcia, Spain; dgutierrez21@outlook.es (D.G.F); amartinez30@ucam.edu (A.M.S.); palcaraz@ucam.edu (P.E.A.)

^2^ C.D. Balopal, Palencia, Spain

^3^ Facultad de Deporte, UCAM Universidad Católica de Murcia, Murcia, Spain 

^4^ Strength & Conditioning Society, Murcia, Spain 

^5^ NAR—Nucleus of High Performance in Sport, São Paulo, Brazil

^6^ Department of Human Movement Sciences, Federal University of São Paulo, Santos, Brazil

* Correspondence: tfreitas@ucam.edu (T.T.F.) 

The ability to maximize neuromuscular performance is essential to execute high-intensity explosive actions [1]. Thus, methodologies such as complex-contrast (CT) (i.e., combination of biomechanically similar high-load, low-velocity and low-load, high-velocity exercises) have been proposed to improve athletic capabilities [2] and are commonly used by strength and conditioning practitioners. Notably, when putting CT into practice, coaches find that the long recovery periods typically employed may hinder its application in real-world settings [3]. Therefore, developing CT time-efficient programming strategies is crucial. The aim of this study was to analyze the acute effects of including different exercises during the intra-contrast rest interval (ICRI) of a CT session on subsequent performance of explosive movements. Nineteen resistance-trained males (age 25.35 ± 3.07 years, body mass 78.15 ± 8.29 kg; height 174.95 ± 6.64 cm) completed, in consecutive weeks in randomized order, three different CT protocols. Programs consisted of three sets of a contrast pair combining three repetitions of the half-squat at 0.6 m/s with five vertical jumps and only differed on the activities performed during the 2 min 30 s ICRI. (1) passive recovery (CCT_PASS_); (2) a mobility drill of the non-involved limbs (CCT_MOB_); and (3) a high-intensity strength exercise of the upper body (i.e., bench press) (CCT_STR_). Countermovement jump (CMJ) and bench press throw (BPT) were evaluated at baseline and after each set during the workout. Rate of perceived exertion was recorded post-session. Data were analyzed using a Linear mixed model fit test. Significant differences (*p* < 0.05) were found for most CMJ and BPT variables among protocols, with CCT_STR_ displaying greater decreases in performance than CCT_PASS_ and CCT_MOB_. Additionally, CCT_STR_ was perceived as more intense than both CCT_PASS_ and CCT_MOB_ (*p* < 0.001). When analyzing each set and repetition separately, results identified that most performance metrics (i.e., CMJ height, peak power or modified reactive strength index and BPT peak velocity) significantly decreased (*p* < 0.05) in the last 2–3 repetitions of each set. From a practical perspective, reducing the number of repetitions per set and increasing the number of sets may be a good programming strategy to minimize performance decreases during a single CT session. Passive ICRIs seem to be more suitable when the aim is to maximize power production and minimize fatigue during the workout. However, in time-limited contexts, including a mobility exercise in the ICRI appears to be a good alternative as CCT_MOB_ displayed smaller declines in CMJ and BPT performance and was perceived as less intense than the CCT_STR_.

**Funding:** This research received no external funding.

**References**

1. Suchomel, T.J.; Nimphius, S.; Stone, M.H. The Importance of Muscular Strength in Athletic Performance. *Sports Med.* **2016**, *46*, 1419–1449. https://doi.org/10.1007/s40279-016-0486-0.
2. Cormier, P.; Freitas, T.T.; Loturco, I.; Turner, A.; Virgile, A.; Haff, G.G; Blazevich, A.J.; Agar-Newman, D.; Henneberry, M.; Baker, D.G.; McGuigan, M.; Alcaraz, P.E.; Bishop, C. Within Session Exercise Sequencing During Programming for Complex Training: Historical Perspectives, Terminology, and Training Considerations. *Sports Med.* **2022**, *52*, 2371–2389. https://doi.org/10.1519/JSC.0000000000003493.
3. Weldon, A.; Duncan, M.M.; Turner, A.; Lockie, R.G.; Loturco, I. Practices of strength and conditioning coaches in professional sports: a systematic review. *Biol. Sport* **2022**, *39*, 715–726. https://doi.org/10.5114/biolsport.2022.107480.

### 2.30. Reliability of Time to Exhaustion above the Power Output at VO_2max_ in Trained Mountain Bikers

Allan Inoue ^1,2,^*, Eduardo Lattari ^3^, Everton Crivoi do Carmo ^4^, Bruno Ribeiro Ramalho Oliveira ^5^, Elirez Bezerra da Silva ^1,2^ and Tony Meireles Santos ^6^

^1^ Exercise and Sport Sciences Postgraduate Program, Rio de Janeiro State University; elirezsilva@cosmevelho.com.br (E.B.S.) 

^2^ Research Group on Exercise and Health Science, Rio de Janeiro State University 

^3^ Physical Activity Sciences Postgraduate Program, Salgado de Oliveira University; eduardolattari@yahoo.com.br (E.L.) 

^4^ Physical Education Department, Senac University Center; evertoncrivoi@hotmail.com (E.C.C.)

^5^ Physical Education and Sports Department, Federal Rural University of Rio de Janeiro; brunoramalho87@gmail.com (B.R.R.O.) 

^6^ Physical Education Department, Pernambuco Federal University; tonymsantos@gmail.com (T.M.S.)

* Correspondence: allan_inoue@hotmail.com (A.I.)

Exercise physiologists have widely used the time-to-exhaustion (TTE) test to assess individuals’ tolerance for specific exercise intensities, with applications for high-intensity interval training prescription [1]. To evaluate the benefits of training interventions, a test needs to be reliable so as to detect changes due to training rather than inter-individual differences or measurement errors [2]. To our knowledge, no study has investigated the reliability of TTE during constant-load trials in mountain bike (MTB) athletes. In the MTB cross-country circuit races, ~30% of the race time is spent above Wmax [3], which highlights the importance of performing numerous efforts above Wmax during the MTB race [4]. In this sense, investigating the reliability of TTE tests at high intensities would be relevant for this modality. Thus, the aim of the present study was to analyze the reliability of the TTE test at intensities above maximum oxygen uptake (VO_2max_) in trained mountain bike cross-country riders. Fifteen male cross-country MTB riders (mean ± SD: age 31.5 ± 6.6 years, stature 174.0 ± 5.4 cm, body mass 67.2 ± 5.1 kg, VO_2max_ 64.5 ± 4.7 mL∙kg^−1^∙min^−1^) completed two TTE tests on the cycle ergometer with four different intensities above the maximal work rate in the incremental test (W_max_) (105%, 120%, 130%, and 140% of W_max_). There was a moderate reliability between TTE tests at 105% (intraclass correlation coefficient (ICC) = 0.81, *p* ≤ 0.001; coefficient of variation (CV) = 9.1%; standard error of measurement (SEM) = 18.3%), and 120% (ICC = 0.88, *p* ≤ 0.001; CV = 6.6%; SEM = 9.3%) W_max_. For intensities of 130% (ICC = 0.53, *p* = 0.018; CV = 9.2%; SEM = 15.8%) and 140% (ICC = 0.56, *p* = 0.012; CV = 12.2%; SEM = 13.5%) W_max_, the reliability results proved to be questionable. In addition, no significant differences were found between the two TTE tests in all intensities (*p* > 0.05). In conclusion, caution should be taken when assessing TTE above VO_2max_ or when using it as a performance indicator given its moderate to low reliability.

**Funding:** This research received no external funding.

**References**

1. Laursen, P.B.; Shing, C.M.; Peake, J.M.; Coombes, J.S.; Jenkins, D.G. Interval training program optimization in highly trained endurance cyclists. *Med. Sci. Sports Exerc.* **2002**, *34*, 1801–1807. https://doi.org/10.1097/00005768-200211000-00017.
2. Atkinson, G.; Nevill, A.M. Selected issues in the design and analysis of sport performance research. *J. Sports Sci.* **2001**, *19*, 811–827. https://doi.org/10.1080/026404101317015447.
3. Prinz, B.; Simon, D.; Tschan, H.; Nimmerichter, A. Aerobic and Anaerobic Power Distribution During Cross-Country Mountain Bike Racing. *Int. J. Sports Physiol. Perform.* **2021**, *16*, 1610–1615, https://doi.org/10.1123/ijspp.2020-0758.
4. Inoue, A.; Sa Filho, A.S.; Mello, F.C.; Santos, T.M. Relationship between anaerobic cycling tests and mountain bike cross-country performance. *J. Strength Cond. Res.* **2012**, *26*, 1589–1593. https://doi.org/10.1519/JSC.0b013e318234eb89.

### 2.31. Differences in Linear and Curvilinear Sprint Performances between Young and Senior Female Soccer Players

**Ronaldo Kobal ^1,2^, Renato Barroso ^2^, Marcelo Rossetti ^1^, Raíssa Jacob ^1^, Lucas A. Pereira ^3,4^ and Irineu Loturco ^3,4,5^**

^1^ Sport Club Corinthians Paulista, São Paulo, Brazil; rk.sportperformance@gmail.com (R.K.); marcelorossetti@outlook.com.br (M.R.); raissajacob@ufrj.br (R.J.) 

^2^ School of Physical Education, University of Campinas, Campinas, Brazil; rbarroso@unicamp.br (R.B.)

^3^ NAR—Nucleus of High Performance in Sport, São Paulo, Brazil; lucasa_pereira@outlook.com (L.A.P.); irineu.loturco@terra.com.br (I.L.) 

^4^ Department of Human Movement Sciences, Federal University of São Paulo, São Paulo, Brazil

^5^ University of South Wales, Pontypridd, Wales, United Kingdom

Sprinting is the most frequent action preceding goal situations in soccer [1]. In elite soccer, sprints are usually performed over short distances (i.e., <10 m), up to 60 times per match, and this varies according to playing position and competitive level [2,3]. Importantly, ~85% of these intense efforts may be classified as “curve sprints” (CS), which highlights the key role played by CS ability in soccer performance [3,4]. Therefore, the proper and progressive development of CS ability is essential for preparing the modern soccer player. A first step in this direction is to examine how this complex physical skill evolves across different age categories. A previous study on male soccer players revealed that under-20 players perform better than under-15 and under-17 players only on the “good side” (i.e., faster CS side), but not on the “weak side” (i.e., slower CS side) [4]. Nonetheless, to date, no study has investigated this phenomenon in female soccer players, especially up to the senior level. The current research project analyzed the evolution of linear and CS performances across different age categories in female soccer. Sixty-one female players from three different age categories (under-17, n = 21; under-20, n = 18; and senior, n = 22) participated. Players were assessed at the beginning of the pre-season and performed the following tests: linear sprint velocity with split times in 10 and 17 m, and the standardized and reliable 17 m CS test [3,4]. An ANOVA one-way was used to test the differences between age categories. The level of significance was set at *p* < 0.05. Senior players showed superior performances in all speed-related measurements (i.e., 10 and 17 m linear sprint velocity, and CS good and weak sides) compared to under-17 and under-20 players (*p* < 0.001 and < 0.004, respectively). No significant differences were detected in any of the tested variables when comparing under-17 and under-20 age categories (*p* ranging from 0.456 to 0.839). In conclusion, senior female soccer players were able to sprint faster than their younger peers, both in linear and curvilinear trajectories. In contrast, linear sprint and CS performances remained unchanged from under-17 to under-20 age categories, which can be interpreted as a negative outcome. Coaches working with young female soccer players are encouraged to reexamine and optimize their speed training strategies, especially during the later stages of players’ specialization (i.e., from 17 to 20 years old).

**Funding:** This research received no external funding.

**References**

1. Faude, O.; Koch, T.; Meyer, T. Straight sprinting is the most frequent action in goal situations in professional football. *J. Sports Sci.* **2012**, *30*, 625–631. https://doi.org/10.1080/02640414.2012.665940.
2. Barnes, C.; Archer, D.T.; Hogg, B.; Bush, M.; Bradley, P.S. The evolution of physical and technical performance parameters in the English Premier League. *Int. J. Sports Med.* **2014**, *35*, 1095–1100. https://doi.org/10.1055/s-0034-1375695.
3. Loturco, I.; Pereira, L.A.; Filter, A.; Olivares-Jabalera, J.; Reis, V.P.; Fernandes, V.; Freitas, T.T.; Requena, B. Curve sprinting in soccer: Relationship with linear sprints and vertical jump performance. *Biol. Sport* **2020**, *37*, 277–283. https://doi.org/10.5114/biolsport.2020.96271.
4. Filter-Ruger, A.; Gantois, P.; Henrique, R.S.; Olivares-Jabalera, J.; Robles-Rodríguez, J.; Santalla, A.; Requena, B.; Nakamura, F.Y. How does curve sprint evolve across different age categories in soccer players? *Biol. Sport* **2022**, *39*, 53–58. https://doi.org/10.5114/biolsport.2022.102867.

### 2.32. Differences in Vertical and Horizontal Mechanical Jump Parameters between Elite Sprinters and National Team Rugby Players

**Irineu Loturco ^1,2,3^, Tomás T. Freitas ^1,2,4,5,6^, Túlio B. M. A. Moura ^1^, Valter P. Mercer ^1^, Victor Fernandes ^1^, Marina T. Betelli ^7^, Ademir F. S. Arruda ^7^ and Lucas A. Pereira ^1,2^**

^1^ NAR—Nucleus of High Performance in Sport, São Paulo, Brazil; irineu.loturco@terra.com.br (I.L.); tuliobernardo@gmail.com (T.B.M.A.M.); vaterpreis@gmail.com (V.P.M.); victorfernandescoach@gmail.com (V.F.); lucasa_pereira@outlook.com (L.A.P.) 

^2^ Department of Human Movement Sciences, Federal University of São Paulo, São Paulo, Brazil

^3^ University of South Wales, Pontypridd, Wales, United Kingdom 

^4^ UCAM Research Center for High Performance Sport, UCAM Universidad Católica de Murcia, Murcia, Spain; tfreitas@ucam.edu (T.T.F.)

^5^ Facultad de Deporte, UCAM Universidad Católica de Murcia, Murcia, Spain

^6^ Strength & Conditioning Society, Murcia, Spain 

^7^ CBRu—Brazilian Rugby Confederation, São Paulo, Brazil; marina.torresbetelli@gmail.com (M.T.B.); fe.schultz@gmail.com (A.F.S.A.)

Sprint performance relies on a range of mechanical and technical factors. It is known, for example, that elite sprinters can orientate the force vector in the horizontal direction more effectively during the acceleration phase of sprinting [1]. Furthermore, at top speed, faster runners usually take longer strides than slower runners by applying greater forces in the vertical direction, which, in turn, increases the vertical velocity, flight time, and distance traveled between successive steps [2]. Mechanical outputs collected from different vertical and horizontal jump measurements are widely used to evaluate an athlete’s ability to produce and apply force in multiple directions and under different conditions [3]. We examined and compared the jump performance of sprinters (faster runners) and rugby players (slower runners) through the analysis of multiple mechanical parameters collected over a series of vertical and horizontal jump tests. Seven elite sprinters and seven rugby players from the Brazilian National Team participated in this research project. After a standardized warm-up (e.g., running at a moderate pace for 10 min, lower limb dynamic stretching, and submaximal attempts of each jump), athletes performed three repetitions of squat, countermovement, and standing long jumps (SJ, CMJ, LJ) on a porTable 3D force plate (Kistler 9260AA3; Kistler Instrument Corp, Winterthur, Switzerland), with their hands on their hips. Between-group differences in vertical–horizontal jump metrics were analyzed using an independent *t*-test. The statistical significance level was set at *p* < 0.05. Regarding vertical jump tests, sprinters exhibited higher SJ and CMJ height (+39% and +43%, respectively) and vertical take-off velocity (+18% and +20%, respectively), and greater relative vertical force production (+15% and +13%, respectively) than rugby players (*p* < 0.05). Regarding the horizontal jump test, higher horizontal take-off velocity (+19%) and greater relative horizontal force production (+36%) were observed for sprinters compared to rugby players (*p* < 0.05). In general, as expected, sprinters were able to achieve higher take-off velocities (hence, jump heights) and apply greater amounts of force onto the ground during both vertical and horizontal jump attempts. Nevertheless, the difference in force output was more pronounced in the horizontal direction (+36%) than in the vertical direction (~14%), which may support previous findings [1,4] and explain the higher rates of acceleration regularly exhibited by these highly specialized athletes. Coaches and practitioners from different sports should emphasize the development of horizontal force production to improve the acceleration capability (and consequently, the top-speed performance) of their athletes.

**Funding:** This research received no external funding.

**References**

1. Morin, J.B.; Edouard, P.; Samozino, P. Technical ability of force application as a determinant factor of sprint performance. *Med. Sci. Sports Exerc.* **2011**, *43*, 1680–1688. https://doi.org/10.1249/MSS.0b013e318216ea37.
2. Weyand, P.G.; Sternlight, D.B.; Bellizzi, M.J.; Wright, S. Faster top running speeds are achieved with greater ground forces not more rapid leg movements. *J. Appl. Physiol. (1985)* **2000**, *89*, 1991–1999. https://doi.org/10.1152/jappl.2000.89.5.1991.
3. Loturco, I.; D’Angelo, R.A.; Fernandes, V.; Gil, S.; Kobal, R.; Cal Abad, C.C.; Kitamura, K.; Nakamura, F.Y. Relationship between sprint ability and loaded/unloaded jump tests in elite sprinters. *J. Strength Cond. Res.* **2015**, *29*, 758–764. https://doi.org/10.1519/jsc.0000000000000660.
4. Colyer, S.L.; Nagahara, R.; Takai, Y.; Salo, A.I.T. How sprinters accelerate beyond the velocity plateau of soccer players: Waveform analysis of ground reaction forces. *Scand. J. Med. Sci. Sports* **2018**, *28*, 2527–2535. https://doi.org/10.1111/sms.13302.

### 2.33. Comparison of Acid-Base Profile in World-Class Sprint and Endurance Paralympic Athletes

Thiago Lourenço *, Amauri Veríssimo, Fábio Breda, Leonardo Araújo and Augusto Barbosa

Brazilian Paralympic Committee; amauriwv@hotmail.com (A.V.); fabio.breda@cpb.org.br (F.B.); leonardo.tomasello@cpb.org.br (L.A.); augusto.barbosa@cpb.org.br (A.B.)

* Correspondence: thiago.lourenco@cpb.org.br (T.L.)

Blood pH maintenance is critical to avoid fatigue during exercise and physiologically support endurance performance [1]. However, this topic is still underexploited within the Paralympic context. This study aimed to compare the resting acid-base profile of Paralympic athletes from different sports. Twenty world-class Paralympic athletes of three different sports participated in this study: athletic sprinters (SPRa, n = 5, three male T36, T37 and T63, and two females T11 and T20); endurance runners (END, n = 9, eight males, one T47 and seven T20) and swimming sprinters (SPRsw, two males S8 and S11, and three females, two S12 and an S14). All of them reached finals in major international competitions (five were medalists in individual or relay events in Tokyo 2020 Paralympic Games). Resting blood samples were collected during the competitive period using a capillary puncture from the fingertip using disposable lancets (Accu-Chek SoftClix^®^, Roche^®^) and a heparinized syringe. Plasma pH, carbonic dioxide partial pressure (pCO_2_), and hemoglobin (Hb) were immediately analyzed on the epoc^®^ Blood Analysis System (Siemens Healthcare Diagnostics Inc., Erlangen, Germany). The bicarbonate ion plasma concentration (HCO_3_^−^) was calculated by deriving pCO_2_ through the Handerson–Hasselbach equation, whereas base excess (BE) was calculated from Hb, HCO_3−_, and pH values by the Van Skyle equation [2]. One way ANOVA revealed no differences (*p* < 0.05) in blood pH (SPRa: 7.49 ± 0.02; END: 7.48 ± 0.09, SPRsw: 7.48 ± 0.19), HCO_3−_ (SPRa: 20.6 ± 0.65; END: 22.0 ± 0.56; SPRsw: 19.8 ± 0.62 mmol·L^−1^); pCO_2_ (SPRa: 27.0 ± 3.28; END: 29.7 ± 0.96; SPRsw: 27.6 ± 3.81 mmHg) or BE (SPRa: −2.62 ± 5.2; END: −1.51 ± 5.3; SPRsw: −3.62 ± 0.70 mmol·L^−1^). These data indicate that specific training stimuli used within Paralympic athletes’ routines of different sports may not induce different adaptations in resting blood acid-base profile. Considering that the sports classification can embed more variability into different sports’ training routines, a more individualized dose–response analysis of the resting blood acid-base profile may be an alternative to monitor possible training-induced adaptations within the Paralympic context.

**Funding:** This research received no external funding.

**References**

1. Joyner, M.J.; Coyle, E.F. Endurance exercise performance: the physiology of champions. *J. Physiol.* **2008**, *586*, 35–44. https://doi.org/10.1113/jphysiol.2007.143834.
2. Lang, W.; Zander, R. The Accuracy of Calculated Base Excess in Blood. *Clin. Chem. Lab. Med.* **2002**, *40*, 404–410. https://doi.org/10.1515/CCLM.2002.065.

### 2.34. Screen Time and Its Association with Physical Activity Levels, Overweight and Obesity in Spanish Children and Adolescents: A Systematic Review

Elena Marín-Cascales ^1,2,^*, César Méndez-Domínguez ^3,4^ and Tomás T. Freitas ^1,2,5,6,7^

^1^ UCAM Research Center for High Performance Sport, UCAM Universidad Católica de Murcia, Murcia, Spain; tfreitas@ucam.edu (T.T.F.)

^2^ Strength & Conditioning Society, Murcia, Spain 

^3^ Department of Physical Education and Sports, Rey Juan Carlos University, Madrid, Spain; cesarmendez4@yahoo.es (C.M.D.) 

^4^ Department of Didactics of Languages, Arts and Physical Education, Complutense University of Madrid, Madrid, Spain

^5^ Facultad de Deporte, UCAM Universidad Católica de Murcia, Murcia, Spain 

^6^ NAR—Nucleus of High Performance in Sport, São Paulo, Brazil

^7^ Department of Human Movement Sciences, Federal University of São Paulo, Santos, Brazil

* Correspondence: emarin@ucam.edu (E.M.C.)

Childhood and adolescent obesity is a major public health issue with increasing levels being observed in recent decades worldwide [1] and in Spain [2]. Several factors, including physical inactivity and the high prevalence of unhealthy lifestyle behaviors, have been identified as main contributors to this epidemic [3]. Among the latter, an elevated amount of screen time has been suggested as an important aspect to be considered [4]. Therefore, investigating the prevalence of this behavior, particularly in Spain, and its association with physical activity levels and obesity rates is important to allow effective strategies to be determined to help address this problem. The aim of the present study was to review and update the scientific literature to examine the extent to which screen time may influence levels of physical activity and overweight and obesity in Spanish children and adolescents. A computerized bibliographic search was carried out in the PubMed, Web of Science and Scopus online databases, compiling research published from 2011 to September 2022. For studies to be included, the following criteria had to be met: (1) publication language had to be English or Spanish; (2) cross-sectional and/or longitudinal design without intervention had to be followed; (3) participants had to be Spanish children and/or adolescents (5–19 years old); (4) screen time (hours in front of the computer, television, mobile phones, tablets, video games, etc.) had to be a study outcome; and (5) physical activity levels and/or obesity-related body composition variables had to be reported. After the screening process, a total of 42 manuscripts were included and the Joanna Briggs Institute critical appraisal tool was used to assess the risk of bias. The main findings indicated that screen time has increased in recent years among Spanish youth, that adolescents seem to spend more hours using screens and that this group appears to be less physically active than children. Moreover, an inverse relationship between screen time and the level of physical activity was evident in most studies, irrespective of the age group. Likewise, an association was observed between the number of screen hours and the body mass index and/or fat mass percentage in children and adolescents. The results of this systematic review suggest that, to improve the health status and body composition parameters of the Spanish youth, multifactorial strategies aimed at reducing the time spent in front of screen devices and increasing the levels of physical activity are of utmost importance.

**Funding:** This research received no external funding.

**References**

1. Di Cesare, M.; Sorić, M.; Bovet, P.; Miranda, J.J.; Bhutta, Z.; Stevens, G.A.; Laxmaiah, A.; Kengne, A.P.; Bentham, J. The epidemiological burden of obesity in childhood: a worldwide epidemic requiring urgent action. *BMC Med.* **2019**, *17*, 212. https://doi.org/10.1186/s12916-019-1449-8.
2. Bravo-Saquicela, D.M.; Sabag, A.; Rezende, L.F.M.; Rey-Lopez, J.P. Has the Prevalence of Childhood Obesity in Spain Plateaued? A Systematic Review and Meta-Analysis. *Int. J. Environ. Res. Public Health* **2022**, *19*, 5240. https://doi.org/10.3390/ijerph19095240.
3. Jebeile, H.; Kelly, A.S.; O’Malley, G.; Baur, L.A. Obesity in children and adolescents: epidemiology, causes, assessment, and management. *Lancet Diabetes Endocrinol.* **2022**, *10*, 351–365. https://doi.org/10.1016/S2213-8587(22)00047-X.
4. Mitchell, J.A.; Rodriguez, D.; Schmitz, K.H.; Audrain-McGovern, J. Greater screen time is associated with adolescent obesity: a longitudinal study of the BMI distribution from Ages 14 to 18. *Obesity (Silver Spring)* **2013**, *21*, 572–575. https://doi.org/10.1002/oby.20157.

### 2.35. Thermodynamics of the Quadriceps and Hamstrings Using Infrared Thermography during a Rectangular Test in the Race Walking World Cup Champions (Oman 2022)

**Francisco Javier Martínez-Noguera ^1^, Cristian Marín-Pagán ^1^, Alessio Cabizosu ^2^, Tomás T. Freitas ^1,3,4,5,6^, Linda H. Chung ^1^ and Pedro E. Alcaraz ^1,4^**

^1^ UCAM Research Center for High Performance Sport, UCAM Universidad Católica de Murcia, Murcia, Spain; fjmartinez3@ucam.edu (F.J.M.N.); cmarin@ucam.edu (C.M.P.); tfreitas@ucam.edu (T.T.F.); lhchung@ucam.edu (L.H.C.); palcaraz@ucam.edu (P.E.A.) 

^2^ THERMHESC Group. Chair of Ribera Hospital de Molina San Antonio UCAM Universidad Católica de Murcia, Murcia, Spain; acabizosu@ucam.edu (A.C.)

^3^ Facultad de Deporte, UCAM Universidad Católica de Murcia, Murcia, Spain

^4^ Strength & Conditioning Society, Murcia, Spain

^5^ NAR—Nucleus of High Performance in Sport, São Paulo, Brazil

^6^ Department of Human Movement Sciences, Federal University of São Paulo, Santos, Brazil

Infrared thermography is a valid tool for measuring and quantifying the metabolic response of the muscular system in sports [1]. The intensity of infrared radiation emitted by body surfaces is converted into a temperature (T^a^) pattern [2]. The purpose of this study was to evaluate how ambient T^a^ (17° vs. 28 °C) affects the skin surface T^a^ of the quadriceps (Q) and hamstrings (H) using a Flir thermographic camera and the kinetics of lactate (La¯) (Lactate Pro) before, during and after a rectangular test in international race walkers. The rectangular test had the following configuration: 15 sets of 2 min at high intensity (14.5 km/h = 90–95% of VO_2PEAK_) interspersed with 2:30 min of recovery at moderate intensity (11.5 km = 70–75% of VO_2PEAK_) between sets. The T^a^ of the Q and H was measured at rest, and after the 5th, 10th and 15th race walking set using an infrared thermographic camera at ambient T^a^ of 17 and 28 °C. In addition, lactate was measured with capillary blood (finger) at the same time points when T^a^ was taken. Friedman’s test was used to evaluate the group x time interaction and Pearson’s test for correlations. Friedman’s test detected a group x time interaction when comparing the evolution of skin T^a^ on the skin surface of Q and H between 17° vs. 28 °C (*p* = 0.020 and *p* = 0.015, respectively) during a rectangular test. Furthermore, there was a group x time interaction (Friedman test) in La¯ during the rectangular test when comparing the two ambient T^a^ (17° vs. 28 °C; *p* = 0.006). We observed a positive correlation in skin surface T^a^ changes between 17° and 28 °C from pre- to post-intervention and between Q and H (r = 0.956). The thermoregulation pattern was similar in Q and H, as there was a downward dynamic in both cases, with higher T^a^ in the warm conditions (28 °C) compared to normal T^a^ (17 °C). In addition, La¯ concentrations were higher at 28 °C compared to 17 °C, indicating greater metabolic stress and involvement of the glycolytic pathway during the rectangular test in international race walkers. Thermography can be a good method to assess metabolic changes induced by increased environmental temperature.

**Funding:** This research received no external funding.

**References**

1. Fernandes Ade, A.; Amorim, P.R.; Brito, C.J.; de Moura, A.G.; Moreira, D.G.; Costa, C.M.; Sillero-Quintana, M.; Marins, J.C. Measuring skin temperature before, during and after exercise: a comparison of thermocouples and infrared thermography. *Physiol. Meas.* **2014**, *35*, 189–203. https://doi.org/10.1088/0967-3334/35/2/189.
2. Cabizosu, A.; Carboni, N.; Martinez-Almagro Andreo, A.; Vegara-Meseguer, J.M.; Marziliano, N.; Gea Carrasco, G.; Casu, G. Theoretical basis for a new approach of studying Emery-Dreifuss muscular dystrophy by means of thermography. *Med. Hypotheses* **2018**, *118*, 103–106. https://doi.org/10.1016/j.mehy.2018.06.027.

### 2.36. Using GPS to Create Acceleration-Velocity Profiles in Brazilian Soccer

**André Martins ^1,2^, Guilherme Ramos ^3^ and Ariclécio Oliveira ^1^**

^1^ Universidade Estadual do Ceará—UECE; andre.martins@uece.br (A.M.) 

^2^ Ceará Sporting Club; fisiologia@cearasc.com

^3^ Confederação Brasileira de Futebol—CBF; guilhermepassosramos@gmail.com (G.R.) 

Based on GPS data, acceleration-speed profile (AccSpd) was presented [1]. This method does not require additional preparation time and physical demand, plus it allows acceleration and speed to be assessed during training and matches where athletes produce large amounts of linear and non-linear accelerations, and sprints with different durations and starting from different initial speeds [2,3]. Based on this method, the present study aimed to (1) show the difference between the AccSpd profile of soccer athletes measured in different contexts of the season between training and matches; and (2) propose the use of this method to establish an individual high-speed running (HSR). Acceleration and speed temporal data were collected from GPS (Catapult^®^ S7) and related to create a linear regression between them. The HSR was defined as the average of 1% of the highest accelerations expressed. Linear regression was created considering only the highest values of speed-related accelerations within the interval between speed of HSR and maximum speed expressed. Three variables were created to characterize the profile: A0 (maximum possible acceleration), S0 (maximum possible speed) and A0/S0 (slope). The AccSpd profiles and their variables were generated with data referring to the days of each analyzed period for comparison. GPS data were collected from players (n = 8) during congested (CONGEST: 3 training days and 3 matches with interval < 72 h) and no congested (NCONGEST: 16 training days and 4 matches with interval > 72 h) match context. A paired samples *t*-test was used to compare the means between groups. Maximum accelerations higher values were found related to speed close to 5 m/s. There was no statistically significant difference between the variables when the profiles were generated with data from matches and training together. However, it was observed that A0 and A0/S0 were higher in CONGEST when analyzing only match data (A0_M_ CONGEST = 8.87 ± 1.23 m/s^2^ and A0_M_ NCONGEST = 6.97 ± 0.87 m/s^2^; A0/S0_M_ CONGEST = 1.1 ± 0.18 and A0/S0_M_ NCONGEST = 0.87 ± 0.12; *p* = 0.001 for both). Analyzing only training data, A0 and S0 were higher in NCONGEST (A0_T_ CONGEST = 6.19 ± 1.78 m/s^2^ and A0_T_ NCONGEST = 8.4 ± 0.92 m/s^2^; *p* = 0.022; S0_T_ CONGEST = 6.23 ± 0.75 m/s and S0_T_ NCONGEST = 8.26 ± 0.014 m/s; *p* = 0.0001). There was no statistically significant difference between the HSR values in any data combination. AccSpd profile can provide information regarding the mechanics of an athlete’s speed actions and can be related to the contextual characteristics of the training and/or match demands, in addition to providing information about HSR.

**Funding:** This research received no external funding.

**References**

1. Morin, J.B.; Mata, Y.L.; Osgnachb C.; Barnabòb, A.; Pilatic, A.; Samozino, P.; Pramperob, P. Individual acceleration-speed profile in-situ: a proof of concept in professional football players. *J. Biomech.* **2021**, *123*, 110524. https://doi.org/10.1016/j.jbiomech.2021.110524.
2. Di Salvo, V.; Baron, R.; Tschan, H.; Calderon-Montero, F.; Bachl, N.; Pigozzi, F. Performance Characteristics According to Playing Position in Elite Soccer. *Int. J. Sports Med.* **2007**, *28*, 222–227. https://doi.org/10.1055/s-2006-924294.
3. Wisloff, U.; Castagna, C.; Helgerud, J.; Jones, R.; Hoff, J. Strong Correlation of Maximal Squat Strength with Sprint Performance and Vertical Jump Height in Elite Soccer Players. *Br. J. Sports Med.* **2004**, *38*, 285–288. https://doi.org/10.1136/bjsm.2002.002071.

### 2.37. The proposition of Cut-Off Points for Isokinetic Dynamometry of Knee Extensors and Flexors in Brazilian Soccer Athletes

Alan Herjes Oliveira da Silva ^1,2,3,^*, Kleber Eloi Gomes Barbão ^1,2,3^, Fabiano Mendes de Oliveira ^1,2,3^, Egberto Batista dos Santos ^1^, Mauri Oliveira Brito Junior ^1^, Andre Luis Perly Lima ^1^, Daniele Fernanda Felipe ^3^ and Braulio Henrique Magnani Branco ^1,2,3^

^1^ Clinisport Prime, Maringa, Parana, Brazil; kleberbarbao@hotmail.com (K.E.G.B.); profabiano.edu@gmail.com (F.M.O.); egbertobs12@hotmail.com (E.B.S.); maurijunior4873@gmail.com (M.O.B.J.); andreperly10@gmail.com (A.L.P.L.); brauliohmagnani@gmail.com (B.H.M.B.) 

^2^ Interdisciplinary Laboratory of Intervention in Health Promotion, Cesumar University, Maringa, Parana, Brazil

^3^ Graduate Program of Health Promotion, Cesumar University, Maringa, Parana, Brazil; daniele.felipe@unicesumar.edu.br (D.F.F.)

* Correspondence: alanherjes@gmail.com (A.H.O.S.)

The assessment of muscle strength, power, and muscle endurance in knee extensors and flexors is relevant in soccer athletes’ rehabilitation/training process [1,2]. The objective of the present study was to develop a normative table for evaluating parameters of strength, power, and muscular endurance for lower-body (knee extensors and flexors) in an isokinetic dynamometer in professional soccer players. Eighty-four professional male athletes were evaluated (aged: 24.2 ± 4.3 years; body mass: 76.6 ± 9.4 kg and height: 179.6 ± 7.9 cm). The collections were carried out between August 2020 and August 2021. Athletes at the Brazilian state and national levels performed isokinetic tests for knee extensors/flexors (Biodex Medical Systems, Shirley, NY, USA) to measure strength (60°/s), power (180°/s), and muscle endurance (300°/s) parameters [2], with the evaluation of dominant and non-dominant sides. The tests followed a familiarization/warm-up protocol. Data were distributed in percentiles: p10, p30, p50, p70, and p90 [2]. Statistical analyses were performed using the SPSS program (version 22.0, IBM, EUA). The results were observed for an angular velocity of 60°/s in the knee extension on the right side: p10 = 200.95 N; p30 = 224.5 N; p50 = 251.40 N; p70 = 282.30 N and p90 = 316.30 N and left: p10 = 196.75 N; p30 = 222.55 N; p50 = 250.90 N; p70 = 272.30 N and p90 = 323.90 N; angular velocity of 180°/s on the right side: p10 = 142.60 N; p30 = 163.35 N; p50 = 177.40 N; p70 = 194.10 N and p90 = 225.45 N and left: p10 = 140.70 N; p30 = 161.15 N; p50 = 178.50 N; p70 = 196.95 N and p90 = 222.85 N and 300°/s angular velocity on the right side: p10 = 109.50 N; p30 = 124.40 N; p50 = 134.20 N; p70 = 147.10 N and p90 = 165.05 N and left: p10 = 107.30 N; p30 = 122.75 N; p50 = 135.55 N; p70 = 146.05 N and p90 = 171.15 N. For knee flexion, the following results were identified for an angular velocity of 60°/s, on the right side: p10 = 110.10 N; p30 = 126.50 N; p50 = 141.15 N; p70 = 151.90 N and p90 = 170.30 N and left: p10 = 106.20 N; p30 = 125.90 N; p50 = 134.80 N; p70 = 147.45 N and p90 = 176.10 N; angular velocity of 180°/s on the right side: p10 = 86.05 N; p30 = 98.70 N; p50 = 109.75 N; p70 = 117.85 N and p90 = 143.15 N and left: p10 = 86.90 N; p30 = 96.20 N; p50 = 106.30 N; p70 = 113.35 N and p90 = 130.40 N and angular velocity of 300°/s on the right side: p10 = 72.25 N; p30 = 86.60 N; p50 = 93.60 N; p70 = 101.85 N and p90 = 116.05 N and left: p10 = 72.25 N; p30 = 82.805 N; p50 = 91.15 N; p70 = 96.70 N and p90 = 113.80 N. The cut-off points can help sports physicians, physical therapists, and exercise physiologists during rehabilitation and training for strength, power, and muscle endurance for knee extensors and flexors in soccer athletes.

**Funding:** This research received no external funding.

**References**

1. Van Melick, N.; Weegen, W.V.D.; Horst, N.V.D. Quadriceps and Hamstrings Strength Reference Values for Athletes With and Without Anterior Cruciate Ligament Reconstruction Who Play Popular Pivoting Sports, Including Soccer, Basketball, and Handball: A Scoping Review. *J. Orthop. Sport. Phys. Ther.* **2022**, *52,* 125–55. https://doi.org/10.2519/jospt.2022.10693.
2. Freedson, P.S.; Gilliam, T.B.; Mahoney, T.; Maliszweski, A.F.; Kastango, K. Industrial torque levels by age group and gender. *Isokinet. Exerc. Sci.* **1993**, *3*, 34–42. https://doi.org/10.3233/IES-1993-3105.

### 2.38. Incidence of Injuries in Elite Brazilian Volleyball and Water Polo Players throughout a Competitive Season

Anderson Oliveira *, Raphael Planas, Andre Avallone, Thiago Batista, Anderson Rodrigues and Cesar Abad

Reference Center of Sport Science of Service of Social Industry (CRCE-SESI); raphael.planas@sesisp.org.br (R.P.); avallone@sesisp.org.br (A.A.); thiago.nascimento@sesisp.org.br (T.B.); anderson.santana@sesisp.org.br (A.R.); cesar.abad@sesisp.org.br (C.A.)

* Correspondence: anderson.orodrigues@sesisp.org.br (A.O.)

Volleyball (VB) and water polo (WP) are defined as overhead sports because they elicit many upper limb technical skills, such as throwing, attacking, and blocking. Thus, shoulder injury and pain are common problems reported by VB and WP athletes [1,2]. However, as VB players compete on land, they have higher lower limb overload than WP players who compete in water (without gravity). Therefore, it would be reasonable to suppose that VB and WP players could show different etiologies, body locations and types of injuries. However, this knowledge remains scarce in the literature. This information may help sports professionals to apply preventive programs (PP) to avoid injuries in VB and WP athletes [3]. Then, the aim of the present study was to verify the incidence of injury during the 2021–2022 competitive season in Brazilian elite VB and WP athletes. The study was approved by a local ethics committee. The data were assessed retrospectively from the department of sports physiotherapy according to the daily physiotherapists’ recordings. Overall, 110 injuries (VB = 55; WP = 55) from 30 elite Brazilian athletes (VB = 16; WP = 14) were reported. Most injuries (81.8%) occurred during training (VB = 42; WP = 48), 15.5% occurred during competition (VB = 11; WP = 6), and only three injuries occurred outside of the sport environment. Fourteen injuries (12.7%) were classified as acute with contact [VB = 7 (12.7%); WP = 7 (12.7%)], 66 (60.0%) as acute without contact [VB = 35 (63.6); WP = 31 (56.4)] and 30 (27.3%) as chronic by overusing [VB = 13 (23.6); WP = 17 (30.9)]. Most VB injuries (n = 37; 67.3%) occurred in the lower limbs, especially in the knee (n = 14; 25.5%) and ankle/feet (n = 11; 20.0%), whereas most WP injuries (n = 26; 47.3%) occurred in the upper limbs, especially in the shoulder (n = 16; 29.1%) and elbow (n = 9; 16.4%). The low back was the most common location of injuries in both sports (VB = (n = 7; 12.7%; WP = (n = 8; 14.5%). Our results suggest that during a full competitive season, both VB and WP athletes present a similar incidence of injuries, but in different locations. Then, a PP for VB players should focus more on the lower limbs, whereas WP athletes should pay more attention to the upper limbs, according to each sport requirement. Finally, exercises designed to prevent low back pain must be applied for both groups.

**Funding:** This research received no external funding.

**References**

1. Lesman, J.; Jóźwik, M.; Domżalski, M.E.; Luceri, A.; Mangiavini, L.; Peretti, G.M.; Luceri, F. Sport injuries in professional volleyball players. *J. Biol. Regul. Homeost Agents Congr. Ital. Orthop. Res. Soc.* **2020**, *34* (4 Suppl. 3), 163–170.
2. Franić, M.; Ivković, A.; Rudić, R. Injuries in water polo. *CMJ* **2007**, *48*, 281–288.
3. Bolling, C; et al. Context matters: revisiting the first step of the ‘sequence of prevention’ of sports injuries. *Sport Med*. **2018**, *48*, 2227–2234. https://doi.org/10.1007/s40279-018-0953-x.

### 2.39. Locomotive and Mechanical Effort Intensities: Do They Represent the Same External Load?

**Bruno Pasquarelli ^1^, Rafael José Silva Barbosa ^2^, Gabriel Gazone dos Santos ^2^, Vinícius Costa ^2^, Carlos Fabiano Machado ^2^, Alejandro Pastor ^1^, Eduardo Rostaiser ^1^ and Rodrigo Aquino ^3^**

^1^ Catapult Group International Ltd., Melbourne, Australia; bruno.pasquarelli@catapultsports.com (B.P.); alejandro.pastor@catapultsports.com (A.P.); eduardo.rostaiser@catapultsports.com (E.R.) 

^2^ Department of Health and High Performance, Clube de Regatas do Flamengo, Rio de Janeiro, RJ, Brazil; rafael.rafael10@hotmail.com (R.J.S.B.); gabriel.gazone@flamengo.com.br (G.G.S.); vinicius.costa@flamengo.com.br (V.C.); fabiano.medici@flamengo.com.br (C.F.M.)

^3^ LabSport, Post-graduate Program in Physical Education, Centre of Physical Education and Sport (CEFD), Federal University of Espírito Santo, Vitória/ES, Brazil; aquino.rlq@gmail.com (R.A.) 

The external load (EL) in team sports can be qualified in locomotive external load (LEL) and mechanical external load (MEL), when using a GPS tracking system [1] or inertial movement sensors, respectively [2]. Often, the intensity ratings are used as important information in training and match monitoring. However, the agreement between LEL and MEL intensity assessment should be examined. In order to investigate the reliability of those effort intensity ratings, in the present exploratory study, 49 young Brazilian footballers, U16 (n = 17), U17 (n = 15), and U20 (n = 17) were monitored in 15 official games through wearable devices (Catapult Vector S7). The considered players played > 70% of a total match. In addition, the metrics were divided by total game duration (min). A k-means cluster analysis was performed to classify both LEL and MEL quantitative efforts into low, medium, and high intensity. The LEL variables used were distance per minute, high-intensity activities and accumulated distance (≥20 km·h^−1^), acceleration (≥2m·s^2^) and deceleration (≥−2m·s^2^) efforts calculated by tracking analysis of movement speed. The MEL clustering was calculated based on amount of change of direction to the right and left, acceleration and deceleration efforts derived by inertial movement analyses, total bouts of explosive efforts and jumps, and PlayerLoad^TM^. The Cohen’s Kappa Coefficient (κ) was calculated to assess the agreement of the intensity ratings in LEL and MEL [3]. The strength of agreement for the kappa coefficient was considered slight (κ = 0.132, SE = 0.07, 95% CI = 0.02–0.24, number of observed agreements = 65/131 [49.62%], *p* = 0.28). The percentages of agreement between LEL and MEL for the low-, medium-, and high-intensity ratings were 59.3%, 32.0% and 20.0%, respectively. Therefore, the results showed that locomotive and mechanical effort intensities are different qualifications for monitoring EL in young football players, and the agreement of these measures decreases as the effort intensity rating increases. However, according to the comprehensive understanding and representative practical application of effort intensities in a match context, LEL and MEL should be distinguished and analyzed as different EL metrics in football.

**Funding:** This research received no external funding.

**References**

1. Ravé, G.; Granacher, U.; Boullosa, D.; Hackney, A.C.; Zouhal, H. How to Use Global Positioning Systems (GPS) Data to Monitor Training Load in the “Real World” of Elite Soccer. *Front. Physiol.* **2020**, *11*, 944. https://doi.org/10.3389/fphys.2020.00944.
2. Iosa, M.; Picerno, P.; Morone, G. Wearable Inertial Sensors for Human Movement Analysis. *Exp. Rev. Med. Devices* **2016**, *13*, 641–659. https://doi.org/10.1080/17434440.2016.1198694.
3. Tang, W.; Hu, J.; Zhang, H.; Wu, P.; He, H. Kappa coefficient: a popular measure of rater agreement. *Shanghai Arch. Psychiatry* **2015**, *27*, 62–67. https://doi.org/10.11919/j.issn.1002-0829.215010.

### 2.40. Training on Sand or Grass Surfaces: Effects on the Linear and Multidirectional Sprint Performance of Elite Young Soccer Players

**Lucas A. Pereira ^1,2^, Renan F. H. Nunes ^3^, Carlos A. Paes ^3^, Juan H. S. Conde ^3^, Luiz F. Novack ^3^, Thiago Kosloski ^3^, Rodrigo L. P. Silva ^3^, Paulo H. S. M. Azevedo ^2^, Tomás. T. Freitas ^1,2,4,5,6^ and Irineu Loturco ^1,2,7^**

^1^ NAR—Nucleus of High Performance in Sport, São Paulo, Brazil; lucas_pereira@outlook.com (L.A.P.); irineu.loturco@terra.com.br (I.L.) 

^2^ Department of Human Movement Sciences, Federal University of São Paulo, São Paulo, Brazil; paulohasmazevedo@gmail.com (P.H.S.M.A.)

^3^ Coritiba Football Club, Curitiba, Brazil; nunesrenan85@hotmail.com (R.F.H.N.); cadre_paes@hotmail.com (C.A.P.); juan.conde@coritiba.com.br (J.H.S.C.); luiz.novack@coritiba.com.br (L.F.N.); thiagokosloski@hotmail.com (T.K.); rodrigo.pignatarodr@gmail.com (R.L.P.S.) 

^4^ UCAM Research Center for High Performance Sport, UCAM Universidad Católica de Murcia, Murcia, Spain; tfreitas@ucam.edu (T.T.F.)

^5^ Facultad de Deporte, UCAM Universidad Católica de Murcia, Murcia, Spain 

^6^ Strength & Conditioning Society, Murcia, Spain 

^7^ University of South Wales, Pontypridd, Wales, United Kingdom

The demands for sprint and powerful actions during soccer matches have progressively increased in recent years, especially in decisive moments of the game (e.g., goal scoring or goal attempts) [1,2]. Therefore, substantial attention has been paid to the proper development of these abilities in soccer athletes [2,3]. The sand surface has been used as a practical and effective way to promote meaningful improvements in the sprint performance of team-sport players [4]. However, the limited level of evidence regarding the effectiveness of this training method requires the development of new experimental studies [4]. This study aimed to test the effects of two training programs, performed on either sand or grass surfaces, on the sprint performance of elite young soccer players over an 8-week inter-season training phase. Fifteen under-20 soccer players were randomly allocated to two training groups, as follows: sand training (n = 7) and grass training (n = 8). Players performed 12 training sessions, comprising hurdle jumps, horizontal jumps, drop jumps, and linear and change-of-direction (COD) sprints. Except for the distinct training surfaces, players from both groups followed the same training routines across the 8 weeks. The physical tests were completed in the following order: sprint speed at 10 and 17 m, curve sprint test (CS), and modified Zigzag COD test. To determine significant differences of pre- and post-measurements between groups, an ANOVA two-way with repeated measures was performed. The statistical level of significance was set as *p* < 0.05. Significant increases were noticed for the sand group in 10 and 17 m linear sprint velocity after the training intervention (*p* < 0.05). No significant differences were observed for 10 and 17 m linear sprint velocities in the grass group when comparing pre- and post-tests (*p* > 0.05). Both sand and grass groups revealed similar increases in the CS performance for both right and left sides, as well as for the COD velocity after the training period (*p* < 0.05). The training programs performed on grass and sand surfaces promoted significant improvements in CS and COD speed performance, while straight sprint increased only in the sand group after the 8-week training period. The sand training surface was proven to be an important training strategy to improve linear and multi-directional sprint velocity in young players, which is of great interest and importance for coaches and sport scientists working in elite soccer.

**Funding:** This research received no external funding.

**References**

1. Faude, O.; Koch, T.; Meyer, T. Straight sprinting is the most frequent action in goal situations in professional football. *J. Sports Sci.* **2012**, *30*, 625–631. https://doi.org/10.1080/02640414.2012.665940.
2. Loturco, I.; Freitas, T.T.; Alcaraz, P.E.; Kobal, R.; Nunes, R.F.H.; Weldon, A.; Pereira, L.A. Practices of strength and conditioning coaches in brazilian elite soccer. *Biol. Sport* **2022**, *39*, 779–791. https://doi.org/10.5114/biolsport.2022.108703.
3. Loturco, I.; Bishop, C.; Freitas, T.T.; Pereira, L.A.; Jeffreys, I. Vertical force production in soccer: Mechanical aspects and applied training strategies. *Strength Cond. J.* **2020**, *42*, 6–15. https://doi.org/10.1519/SSC.0000000000000513.
4. Pereira, L.A.; Freitas, T.T.; Marín-Cascales, E.; Bishop, C.; McGuigan, M.R.; Loturco, I. Effects of training on sand or hard surfaces on sprint and jump performance of team-sport players: A systematic review with meta-analysis. *Strength Cond. J.* **2021**, *43*, 56–66. https://doi.org/10.1519/SSC.0000000000000634.

### 2.41. Effects of Sprint and Jump Training on Sand or Grass Surfaces on the Physical Performance of Elite Young Soccer Players

**Lucas A. Pereira ^1,2^, Tomás T. Freitas ^1,2,3,4,5^, Santiago Zabaloy ^6,7^, Ricardo C. A. Ferreira ^2^, Matheus L. Silva ^8^, Paulo H. S. M. Azevedo ^2^ and Irineu Loturco ^1,2,9^**

^1^ NAR—Nucleus of High Performance in Sport, São Paulo, Brazil; lucasa_pereira@outlook.com (L.A.P.); irineu.loturco@terra.com.br (I.L.) 

^2^ Department of Human Movement Sciences, Federal University of São Paulo, São Paulo, Brazil; ricardocalves@hotmail.com (R.C.A.F.); paulohsmazevedo@gmail.com (P.H.S.M.A.)

^3^ UCAM Research Center for High Performance Sport, UCAM Universidad Católica de Murcia, Murcia, Spain; tfreitas@ucam.edu (T.T.F.) 

^4^ Facultad de Deporte, UCAM Universidad Católica de Murcia, Murcia, Spain

^5^ Strength & Conditioning Society, Murcia, Spain 

^6^ Faculty of Physical Activity and Sports, University of Flores, Buenos Aires, Argentina; santiagozabaloy@hotmail.com (S.Z.) 

^7^ Faculty of Sports Sciences, Pablo de Olavide University, Seville, Spain

^8^ Secretaria de Esportes e Qualidade de Vida, Prefeitura Municipal de São José dos Campos, São José dos Campos, Brazil; matheusluisef@gmail.com (M.L.S.) 

^9^ University of South Wales, Pontypridd, Wales, United Kingdom

Optimization of sprint speed and muscle power is essential in a wide variety of sports [1,2]. Therefore, coaches are constantly searching for more effective and practical ways to improve these capacities in elite athletes [3,4]. Plyometric training is one of the most effective and commonly prescribed speed-power training methods [2,4]. Plyometrics are usually performed on firm surfaces (e.g., wood or grass) with the rationale that soft surfaces (e.g., sand) may lead to greater dissipation of stored elastic energy, thus impairing the proper development of neuromechanical qualities [2]. Nevertheless, a recent meta-analysis [2] revealed that sand surfaces are as effective as harder surfaces to improve both sprint and jump performance in team-sport players. Considering the feasibility of implementing training programs on sand and the low number of controlled trials on this topic, the need to test the actual effectiveness of this potential strategy is evident. Therefore, the aims of this research project were to (1) examine the effects of a 6-week sand training program including both sprint and jump exercises on the speed-related and vertical jump (VJ) performance of elite young soccer players, and (2) compare the changes induced by a sand training scheme with those induced by a similar training program (in terms of volume, intensity, and exercise types) performed on grass. Twenty-four under-20 soccer players were randomly allocated to two training groups (i.e., “sand” or “grass” groups). Athletes performed VJs, 20 m linear sprint, and Zigzag change-of-direction speed tests at pre-, mid- (after six training sessions), and post-intervention (after 12 training sessions). An ANOVA two-way with repeated measures along with effect sizes (ES) were employed to analyze the differences between groups at each one of the three testing points. The statistical level of significance was set as *p* < 0.05. Both groups exhibited similar increases in VJ and Zigzag performance after the 6-week training period (*p*-values ranging from 0.0001 to 0.025; ES ranging from 1.05 to 3.78, for main effect of time). No significant changes were detected for linear sprint velocity in either group (*p*-values ranging from 0.079 to 1.00; ES ranging from 0.07 to 0.65, for main effect of time). In summary, training on sand or grass surfaces resulted in similar improvements in the sprint and jump performance of elite young soccer players. This study confirms the current evidence on the effectiveness of both soft and harder training surfaces in improving the speed- and power-related performance of team-sport athletes.

**Funding:** This research received no external funding.

**References**

1. Loturco, I.; Bishop, C.; Freitas, T.T.; Pereira, L.A.; Jeffreys, I. Vertical force production in soccer: Mechanical aspects and applied training strategies. *Strength Cond. J.* **2020**, *42*, 6–15. https://doi.org/10.1519/SSC.0000000000000513.
2. Pereira, L.A.; Freitas, T.T.; Marín-Cascales, E.; Bishop, C.; McGuigan, M.R.; Loturco, I. Effects of training on sand or hard surfaces on sprint and jump performance of team-sport players: A systematic review with meta-analysis. *Strength Cond. J.* **2021**, *43*, 56-66. https://doi.org/10.1519/SSC.0000000000000634.
3. Freitas, T.T.; Calleja-González, J.; Carlos-Vivas, J.; Marín-Cascales, E.; Alcaraz, P.E. Short-term optimal load training vs a modified complex training in semi-professional basketball players. *J. Sports Sci.* **2019**, *37*, 434-442. https://doi.org/10.1080/02640414.2018.1504618.
4. Loturco, I.; Kobal, R.; Kitamura, K.; Abad, C.C.C.; Faust, B.; Almeida, L.; Pereira, L.A. Mixed training methods: Effects of combining resisted sprints or plyometrics with optimum power loads on sprint and agility performance in professional soccer players. *Front. Physiol.* **2017**, *8*, 1034. https://doi.org/10.3389/fphys.2017.01034.

### 2.42. Influence of Training Load on Neuromuscular Parameters of Professional Soccer Players: Effects of Preseason vs. Competitive Season

**Ezequias Pereira-Neto ^1^, Diêgo Augusto ^2,3^, Leandro Brandão ^4^, Rafael Henrique ^5^, Fabiano Fonseca ^6^, Pedro Menezes ^7,8^ and Fabio Yuzo Nakamura ^9^**

^1^ Associação Desportiva Confiança, Aracaju-SE, Brazil; neto.pereiraedf@gmail.com (E.P.N.) 

^2^ Laboratory of Soccer Studies (LABESFUT), Post-graduate Program in Exercise and Sport Sciences; diegoaugustoufs@gmail.com (D.A.)

^3^ Institute of Physical Education and Sports, State University of Rio de Janeiro, Rio de Janeiro, Brazil

^4^ Federal University of Minas Gerais, The Center of Cognition and Action Research (CECA-UFMG), Belo Horizonte, MG, Brazil; leeo.henriquee01@gmail.com (L.B.)

^5^ Federal University of Pernambuco, Recife, PE, Brazil; rafael.shenrique@ufpe.br (R.H.) 

^6^ Federal Rural University of Pernambuco, Recife, PE, Brazil; dr.fsfonseca@gmail.com (F.F.)

^7^ Clube de Regatas Flamengo, Rio de Janeiro, Brazil; pedromenezes@connectfit.com.br (P.M.) 

^8^ Federal University of Rio de Janeiro, Rio de Janeiro, RJ, Brazil

^9^ Research Center in Sports Science, Health Science and Human Development (CIDESD), University of Maia (ISMAI), Maia, Portugal; fabioy_nakamura@yahoo.com.br (F.Y.N.)

In soccer games, relevant moments, such as goals, are preceded by high-intensity neuromuscular actions (e.g., sprint and jump ability) which may change at different stages of the season [1]. The loads quantified during the preseason in soccer (period lasting between four and six weeks) are described as being of high magnitude. For example, higher perceived exertion (PE) session values are presented during the preseason (4343 ± 329 a.u.) in comparison to the competitive season (1703 ± 173 a.u.) [2]. Moreover, some studies show a negative relationship between training load and strength, power and speed in periods of high loads in the preseason [3]. Therefore, our study investigated the influence of preseason and competitive season training loads on neuromuscular performance in professional soccer athletes. Eleven professional soccer players (26.3 ± 4.5 years; 75.8 ± 8.2 kg; 178.6 ± 7.5 cm) from the second division of the Brazilian soccer championship were evaluated. Linear speed of 20 m (SPD20m), change of direction speed (Zigzag COD Test) and countermovement jump (CMJ) were assessed at the beginning of preseason (M1), after five weeks of preseason (M2) and three weeks after the start of the competitive season (M3). The training load was quantified using the PE scale proposed by the scientific literature [4]. A paired *t*-test was used to compare the loads of the preseason vs. competitive season and repeated measures ANOVA to compare the outcomes at the evaluated moments, adopting *p* ≤ 0.05. The results showed higher PE values in M2 (2281 ± 137 a.u.) than M3 (1484 ± 204 a.u.). Moreover, significant differences in three investigated outcomes were observed between M2 and M3 periods (M2: CMJ: 39.3 ± 4 cm vs. M3: 44.4 ± 5 cm, *p* = 0.001; SPD20m: 6.79 ± 0.28 m·s^−1^ vs. 6.84 ± 0.17 m·s^−1^, *p* = 0.003; ZigzagCOD: 3.85 ± 0.12 m·s^−1^ vs. 4.54 ± 0.13 m·s^−1^, *p* = 0.001). In addition, there were no significant differences between M1 and M3 periods in the CMJ (*p* = 0.111) and SPD20m (*p* = 1.000). We concluded that high training loads can negatively affect neuromuscular performance such as linear speed, change of direction and jumping ability in comparison to training periods with lower training loads. Training load was higher in the preseason in comparison to competitive season.

**Funding:** This research received no external funding.

**References**

1. Faude, O.; Koch, T.; Meyer, T. Straight sprinting is the most frequent action in goal situations in professional football. *J. Sports Sci.* **2012**, *30*, 625–631. https://doi.org/10.1080/02640414.2012.665940.
2. Jeong, T.S.; Reilly, T.; Morton, J.; Bae, S.W.; Drust, B. Quantification of the physiological loading of one week of “pre-season” and one week of “in-season” training in professional soccer players. *J. Sports Sci.* **2011**, *29*, 1161–1166. https://doi.org/10.1080/02640414.2011.583671.
3. Arcos, A.L.; Martínez-Santos, R.; Yanci, J.; Mendiguchia, J.; Méndez-Villanueva, A. Negative Associations between Perceived Training Load, Volume and Changes in Physical Fitness in Professional Soccer Players. *J. Sports Sci. Med.* **2015**, *14*, 394–401.
4. Foster, C.; Florhaug, J.A.; Franklin, J.; Gottschall, L.; Hrovatin, L.A.; Parker, S.; Doleshal, P.; Dodge, C. A new approach to monitoring exercise training. *J. Strength Cond. Res.* **2001**, *15*, 109–115.

### 2.43. Match-Play Demands of Elite Female Team Sports: A Systematic Review

María Luciana Pérez Armendáriz ^1^, Konstaninos Spyrou ^1,2,^* and Pedro E. Alcaraz ^1^

^1^ UCAM Research Center for High Performance Sport, UCAM Universidad Católica de Murcia, Murcia, Spain; mlperez4@alu.ucam.edu (M.L.P.A.); palcaraz@ucam.edu (P.E.A.) 

^2^ Facultad de Deporte, UCAM Universidad Católica de Murcia, Murcia, Spain

* Correspondence: kspyrou@ucam.edu (K.S.)

In female athletes, performance data are often supported by evidence derived from male counterparts [1], and sport practitioners should understand better the demands during match-play in female team sports [2]. This systematic review aimed to review the scientific literature and quantify the demands of the game in elite women’s team sports (i.e., soccer, rugby, field hockey, basketball, handball, and futsal). The study was carried out following PRISMA guidelines and considered all the published articles until July 2022 from three scientific databases (PubMed, Scopus, and Web of Science). A total of 79 studies met the inclusion criteria, of which 37 were in soccer, 21 rugby, 7 field hockey, 7 basketball, 6 handball and 1 futsal. The main results were the following: (1) female soccer players covered ~9539 m total distance (TD) per match, of which ~1418 m was in medium-speed running (MSR), ~559 m in high-speed running (HSR), and ~282 m in sprinting. Also, they performed ~30 sprints, ~152 acceleration (ACC) and ~134 deceleration (DEC) actions; (2) female players in rugby league and union covered ~5453 m TD, of which ~934 m in MSR, ~144 m in HSR, and performed ~7 sprints, while in rugby 7s players covered ~1549 m TD, of which ~355 m in MSR, ~165 m in HSR and ~108 m in sprinting, also completed ~5 sprint actions per match; (3) female field hockey players covered ~5431 m TD, of which ~823 m, ~501 m and ~371 m were in MSR, HSR, and sprinting respectively, and performed a total of ~39 sprint, ~26 ACC and ~32 DEC actions; (4) in basketball, the players covered ~7039 m TD, of which ~16% was in MSR, ~7% in HSR, and ~7% in sprinting, realized ~35 sprint actions; (5) handball players covered ~3442 m TD per match, of which ~423 m were in MSR and ~141 m in HSR, moreover their player load was ~9 a.u·min^−1^ and performed ~8.7–~2.3 ACC and DEC actions per minute; and lastly (6) female futsal players performed ~5 m·min^−1^ in HSR and ~0.4 ACC per minute (4–5 m·s^−2^), also covered a total of ~240 m·min^−1^ in ACC and did ~28 ACC and DEC actions per minute. In conclusion, women’s elite team sports have high physical demands and are different between sports. In applied settings, sports practitioners could use these data for planning a better and more tailored-made training and return-to-play sessions according to sport and player’s individuality.

**Funding:** This research received no external funding.

**References**

1. Elliott-Sale, K.J.; Minahan, C.L.; de Jonge, X.A.K.J.; Ackerman, K.E.; Sipilä, S.; Constantini, N.W.; Lebrun, C.M.; Hackney, A.C. Methodological Considerations for Studies in Sport and Exercise Science with Women as Participants: A Working Guide for Standards of Practice for Research on Women. *Sports Med.* **2021**, *5*, 843–861. https://doi.org/10.1007/s40279-021-01435-8.
2. Nassis, G.P.; Brito, J.; Tomás, R.; Heiner-Møller, K.; Harder, P.; Kryger, K.O.; Krustrup, P. Elite women’s football: Evolution and challenges for the years ahead. *Scand. J. Med. Sci. Sports* **2022**, *32*, 7–11. https://doi.org/10.1111/sms.14094.

### 2.44. Comparison of Jump and Acceleration Ability between Different Match Levels of American Football in Brazil

**José Pimentel ^1^, Bruno Rosa ^2^, André Gomes ^2^ and Eduardo Penna ^1^**

^1^ University Federal State of Pará, Pará 68746630; contato@pimentelfootball.com (J.P.); eduardomp@ufpa.br (E.P.) 

^2^ Brazilian Confederation of American Football, São Paulo 04004030; brunorosa8@gmail.com (B.R.); andre.zevedo@gmail.com (A.G.)

American football in general has a great demand in terms of acceleration; however, this demand is higher or lower according to the match position, specifically on the defensive line (DL) [1]. Therefore, the ability to accelerate could discriminate the competitive level of practitioners. The aim of this study was to compare athletes of different levels in jump and acceleration abilities. Twelve DL athletes were evaluated, five of the first division in Brazilian league (BL) (age = 25 ± 3 years; body mass = 124.7 ± 21.1 kg; height = 184.8 ± 10.2 cm) and seven to the national team (NT) (age = 31 ± 3 years; body mass = 118.4 ± 14.9 kg; height = 190.4 ± 7.3 cm). We collected (in different days) three countermovement (CMJ) and squat jumps (SJ) followed by five-meter linear sprint (LS) using a video made on iPhone X in slow motion (240 fps) analyzed in MySprint and MyJump apps. For jumps, participants were instructed to maintain hands on the hips, and jump as high as possible, without knee or hip flexion (during aerial phase). For sprint, athletes were instructed to use the usual stance (three points) and run as fast as possible after the five-meter mark. The mean of three jumps and the best sprint were used for analysis. Statistical analysis was performed using GraphPad Prism version 8.0.0 for Windows (GraphPad Software, San Diego, CA, USA). An independent *t*-test and Cohen’s d effect size (ES) were used to compare groups, the significance level was set at *p* ≤ 0.05. The SJ of the BL athletes was 28.41 ± 2.7 cm against 31.7 ± 7.5 cm the NT, (*p* = 0.3682; ES = 0.59); in CMJ = 30.7 ± 2.8 cm for BL against 33.3 ± 7.8 cm for NT (*p* = 0.3969; ES = 0.55), and to LS = 1.64 ± 0.05 s for BL against 1.38 ± 0.10 s for NT (*p* = 0.0004; ES = 3.21). The present results indicate that impulse-based capacities such as SJ and CMJ do not significantly differ between Brazilian DL athletes. However, NT athletes displayed a superior ability to accelerate fast, a difference that may be due to kinematic efficiency aspects of force application during acceleration, such as ground contact time [2] or other factors related with force orientation [3]. From the practical perspective and for future studies, it appears necessary to understand and applicate the kinetics and kinematics of running acceleration to train football athletes for specific DL positions.

**Funding:** This research received no external funding.

**References**

1. Wellman, A.D.; Coad, S.C.; Goulet, G.C.; McLellan, C.P. Quantification of competitive game demands of NCAA division I college football players using global positioning systems. *J. Strength Cond. Res.* **2016**, *30*, 11–19. https://doi.org/10.1519/JSC.0000000000001206.
2. Lockie, R.G.; Murphy, A.J.; Knight, T.J.; De Jonge, X.A.J. Factors that differentiate acceleration ability in field sport athletes. *J. Strength Cond. Res.* **2011**, *25*, 2704–2714. https://doi.org/10.1519/JSC.0b013e31820d9f17.
3. Hicks, D.S.; Schuster, J.G.; Samozino, P.; Morin, J.B. Improving mechanical effectiveness during sprint acceleration: practical recommendations and guidelines. *Strength Cond. J.* **2020**, *42*, 45–62. https://doi.org/10.1519/SSC.0000000000000519.

### 2.45. Are There Differences in Change of Direction Deficit Between Genders in Brazilian National Flag Football Teams?

**José Pimentel ^1^, Fernando Domiciano ^2^, Luciano Xavier ^2^ and Eduardo Penna ^1^**

^1^ University federal state of Pará, Pará 68746630, Brazil; contato@pimentelfooball.com (J.P.); eduardomp@ufpa.br (E.P.) 

^2^ Brazilian Confederation of American Football, São Paulo 04004030, Brazil; emaildofepersonal@gmail.com (F.D.); coachtrindade@gmail.com (L.X.)

Change of direction deficit (CODD%) has been reported as an additional metric for scientists and coaches to understand the differences between linear (LS, in seconds) and change of direction speeds (COD, in seconds) in a simple percentage-based calculation [1]. Additionally, significant differences in COD performance between males and females in different sports have been reported [2]. Therefore, this study aimed to investigate CODD% differences in flag football players from Brazilian national teams of both genders. Twenty male (age: 27 ± 4.4 years; body mass: 80.2 ± 10.2 kg; height: 179.2 ± 6.7 cm) and thirteen female athletes (age: 31 ± 3.3 years; body mass: 60.3 ± 9.8 kg; height: 165.3 ± 9.6 cm) participated in this study. All data were collected on two different days for each national team, separated by a month. All participants performed standardized tests, including 20 m linear sprint and Pro-agility (5-10-5) tests. For the 20 m sprint test, participants were instructed to position themselves 0.3 m behind the starting line and performed two maximal sprints with 3 min rest between them. For the Pro-agility test, athletes were positioned in front of equipment and sprinted until marks of 180° turn until completing 20 m. Both tests were completed on an indoor running track and the fastest time was considered for analysis. Statistical. An Independent *t*-test was used to compare male and female groups, and the significance level was set at *p* ≤ 0.05. The male group results were LS = 3.04 ± 0.1 s, COD = 5.01 ± 0.2 s, CODD = 64.7 ± 7.8% and females were LS = 3.42 ± 0.1s, COD = 5.51 ± 0.2s, CODD = 61.1 ± 3.1%. Significant differences were observed between groups in LSs (*p* = 0.0001) and CODs (*p* = 0.0001), although these differences did not remain in CODD% (*p* = 0.1246). The present results indicate that male athletes displayed a superior speed when sprinting and changing direction, in agreement with previous literature [2]. However, the efficiency to change direction relative to linear sprint times does not differ, independent of these differences. From a practical perspective, both male and female players need to incorporate acceleration, deceleration, and technical drills in training to reduce CODD%.

**Funding:** This research received no external funding.

**References**

1. Freitas, T.T.; Pereira, L.A.; Alcaraz, P.E.; Azevedo, P.H.; Bishop, C.; Loturco, I. Percentage-Based Change of Direction Deficit: A New Approach to Standardize Time-and Velocity-Derived Calculations. *J. Strength Cond. Res.* **2021**, *36*, 3521–3526. https://doi.org/10.1519/JSC.0000000000004118.
2. Freitas, T.T.; Alcaraz, P.E.; Calleja-González, J.; Arruda, A.F.; Guerriero, A.; Kobal, R.; Reis, V.P.; Pereira, L.A.; Loturco, I. Differences in change of direction speed and deficit between male and female national rugby sevens players. *J. Strength Cond. Res.* **2021**, *35*, 3170–3176. https://doi.org/10.1519/JSC.0000000000003195.

### 2.46. Forwards and Backs in Rugby: A Strength, Power and Speed Comparison

Maurício Ramos ^1^, Valter P. Mercer ^2^ and Ademir F. S. Arruda ^1,^*

^1^ Brazilian Rugby Confederation; mauricioramos90@gmail.com (M.R.)

^2^ NAR—Nucleus of High Performance in Sport, São Paulo, Brazil; valterpreis@gmail.com (V.P.M.)

* Correspondence: fe.schultz@gmail.com (A.F.S.A.)

Rugby is an intermittent sport that involves contact situations in which speed, power, and strength are key for performance. High levels of strength and speed allow players to have a greater ability to gain the defense line, break tackles, evade defenders, and score more tries. This study aimed to compare forwards (F) and backs (B) in strength, speed, and power considering absolute and relative values to body mass. Eighteen professional rugby players divided into forwards (9) and backs (9) were submitted to tests of squat 1 RM (kg), 10 and 30 m sprint time (s), Half squat (HS), and jump squat (JS). HS and JS were analyzed by the mean propulsive power (MPP) as suggested for assessing power in these exercises [1]. All variables were considered as absolute and relative to body mass; to add an important variable for this population, sprint momentum (mean speed × body mass—kg/m/s) was analyzed. An independent *t*-test, considering *p* < 0.05, was used to identify differences in each variable. As result, it was shown that 30 m sprint time was lower for backs vs. forwards (4.16 ± 0.03 s vs. 4.33 ± 0.04 s). Forwards had higher values for mass (109.9 ± 2.8 kg vs. 89.4 ± 3.9 kg), squat 1 RM (194.4 ± 8.35 kg vs. 170.6 ± 6.37 kg), 10 m momentum (617.9 ± 12.31 kg/m/s vs. 513.7± 25.34 kg/m/s), 30 m momentum (762 ± 15.87 kg/m/s vs. 645.8 ± 28.45 kg/m/s) and Half squat (867.8 ± 26.74 w vs. 739 ± 33.48 w). For 10 m (F = 1.78 ± 0.02 s vs. B = 1.75 ± 0.02 s), jump squat (F = 1045 ± 34.66 w vs. B = 956.4 ± 59.12 w), relative jump squat (F = 9.59 ± 0.43 w/kg vs. B = 10.67 ± 0.41 w/kg) and relative Half squat (F = 7.93 ± 0.24 w/kg vs. B = 8.31 ± 0.29 w/kg), no significant differences were found. Backs were found to be faster than forwards in longer distances, as already shown among the international-level players [2]. On the other hand, forwards showed a higher mass, 1RM squat, and speed momentum indices for both 10 and 30 m. These results suggest that 1RM squat and mass can be important for contact situations, which can impact performance in this specific sport [1,3].

**Funding:** This research received no external funding.

**References**

1. Freitas, T.; Pereira, L. Influence of Strength and Power Capacity on Change of Direction Speed and Deficit in Elite Team-Sport Athletes. *JHK* **2019**, *68*, 167–176. https://doi.org/10.2478/hukin-2019-0069.
2. Hart, A.; Erskine, R. Physical Characteristics Explain Ball-Carrying Capability in Sub-Elite Rugby Union Players. *JSSE* 2022, *ahead of print*. https://doi.org/10.1519/JSC.0000000000002827.
3. Zabaloy, S.; Giráldez, J. In-Season Assessment of Sprint Speed and Sprint Momentum in Rugby Players According to the Age Category and Playing Position. *JHK* **2021**, 77, 274–286. https://doi.org/10.2478/hukin-2021-0025.

### 2.47. External Load Comparison between Positions in Professional Rugby XV Players

Maurício Ramos, Walter Schildberg and Ademir F.S. Arruda *

Brazilian Rugby Confederation; mauricioramos90@gmail.com (M.R.); waltschildberg@gmail.com (W.S.)

* Correspondence: fe.schultz@gmail.com (A.F.S.A.)

Rugby union is a collision-intermittent sport, where the players are divided into two basic positions: forwards (eight players) and backs (seven players). As shown in European competitions, backs have more external loads in displacement measures; on the other hand, forwards have more collision demands [1,2,3]. However, it is not clear if this pattern happens in a South American competition. Thus, this study aimed to compare the demands of these two groups in a professional competition, via global positioning system (GPS) metrics, expressed by meters per minute (m/min), high-speed distance per minute (HSD/min), and high acceleration per minute (ACC/min), and via video analysis, expressed by the number of collisions (NOC). Data were collected from the 10 matches played by a professional franchise in the South American Rugby League (SLAR). The GPS data were collected with *S7* devices from *Catapult Sports* for m/min, HSD/min (equal or above 15 km/h), and ACC/min (equal or above 2.8 mts/s^2^). For the collision variables, *Sports Code Program* was used to identify and quantify the number of tackles and ball carries in a contact situation. Only athletes who played at least 60 min in each match were considered and the mean values for these two positional groups were presented. After an independent *t*-test analysis considering *p* < 0.05 as a significance level, it was verified that, for the displacement variables collected, backs had higher values (m/min: 73.9 ± 8.8 vs. 65.4 ± 6.1 [*p* < 0.0001]; HSD/min: 13.5 ± 3.1 vs. 7.4 ± 2.8 [*p* < 0.0001]; ACC/min: 0.35 ± 0.15 vs. 0.19 ± 0.10 [*p* < 0.0001]). However, the values attributed to collisions were higher for forwards (18.1 ± 3.1 vs. 13.1 ± 1.8 [*p* = 0.0003]). These results suggest that, as already shown in international rugby leagues, forwards have a lower demand for displacements compared to backs; meanwhile, they are more involved in actions of collisions that occur during a match [1,2,3]. Different from these studies, backs have shown higher levels of ACC/min and more attention should be given to understanding these differences. The present results indicate a way to direct the physical and technical training of a team to prepare athletes with more specificity, considering that the demands of the game are displacements for backs and collisions for forwards.

**Funding:** This research received no external funding.

**References**

1. Pollard, B.; Turner, A. The ball in play demands of international rugby union. *JSMS* **2018**, *21*, 1090–1094. https://doi.org/10.1016/j.jsams.2018.02.015.
2. Cahill, N.; Lamb, K. The movement characteristics of English Premiership rugby union players. *JSS* **2013**, *31*, 229–237. https://doi.org/10.1080/02640414.2012.727456.
3. Quarrie, K.L.; Hopkins, W.G.; Anthony, M.J.; Gill, N.D. Positional demands of international rugby union: Evaluation of player actions and movements. *JSMS* **2013**, *16*, 353–359. https://doi.org/10.1016/j.jsams.2012.08.005.

### 2.48. Relationship between Rate of Perceived Exertion, Strength, and Change of Direction Performance in Elite Futsal Athletes

Renato Ribeiro Azevedo and Felipe Pivetta Carpes *

Universidade Federal do Pampa, Laboratory of Neuromechanics, Uruguaiana, RS, Brazil

* Correspondence: carpes@unipampa.edu.br (F.P.C.)

Change of direction (COD) requires acceleration and deceleration ability and reverse or change movement direction which involves knee flexor and extensor strength [1]. Consequently, concentric, and eccentric muscle strength is important to sustain kicking, jumping, tackling, turning, changing pace, decelerating, and sprinting [2]. Although the neuromuscular capacity to produce strength is a mandatory demand for futsal athletes, little is known about any relationship with internal training load and the performance of functional tasks. In this study, we determined the relationship between internal loads, muscle strength, and performance in futsal elite athletes performing locomotion tasks requiring linear acceleration and change of direction (COD). The athletes were evaluated during a regular week of training as part of their competitive season. We quantified the internal load by the rate of perceived exertion in the training sessions (RPE Borg scale), muscle strength (isokinetic peak torque at 60 deg/s), linear speed (20 m run), and change of direction speed (COD zigzag speed test) in the 24 h after the last training session of the 27th week of the season in 14 professional male futsal athletes. Athletes achieved training load in the testing week: 6233 ± 369 a.u.; COD speed: 3.8 ± 0.5 m/s; concentric knee flexor strength right: 2.0 ± 0.5 Nm/kg; concentric knee flexor strength left: 1.9 ± 0.5 Nm/kg; eccentric knee flexor strength right: 2.7 ± 0.4 Nm/kg; eccentric knee flexor strength left: 2.5 ± 0.6 Nm/kg; concentric H:Q ratio right: 138.4 ± 28 %; eccentric H:Q ratio left: 119.3 ± 32 %, and linear speed: 6.4 ± 0.4 m/s. Internal load was inversely related with COD speed (r = −0.59, *p* = 0.02), concentric (right: r = −0.54, *p* = 0.02; left: r = −0.56, *p* = 0.04) and eccentric knee flexor strength (right: r = −0.62, *p* = 0.02). COD deficit was directly related to internal load (r = 0.54, *p* = 0.04), inversely correlated with concentric (right: r = −0.602, *p* = 0.02), and directly related with eccentric H:Q ratio (right: r = 0.58, *p* = 0.03). The linear speed showed significant correlation with the peak isokinetic flexor torque for both right (r = 0.68, *p* < 0.01) and left (r = 0.53, *p* = 0.04) leg. We conclude that the inverse relationship between internal load and strength and speed capacity suggests that knee flexor weakness influences internal load in the futsal athletes evaluated. Furthermore, stronger knee flexors may favor the performance of linear speed, as observed in sprint actions.

**Funding:** This study was financed in part by the Coordenação de Aperfeiçoamento de Pessoal de Nível Superior—Brasil (CAPES) under Grant Finance Code 001. 

**References**

1. Dos Santos, T.; McBurnie, A.; Thomas, C.; Comfort, P.; Jones, P.A. Biomechanical Determinants of the Modified and Traditional 505 Change of Direction Speed Test. *J. Strength Cond. Res.* **2019**, *34*, 1285-1296. https://doi.org/10.1519/JSC.0000000000003439.
2. Caetano, F.G.; de Oliveira Bueno, M.J.; Marche, A.L.; Nakamura, F.Y.; Cunha, S.A.; Moura, F.A. Characterization of the Sprint and Repeated-Sprint Sequences Performed by Professional Futsal Players, According to Playing Position, During Official Matches. *J. Appl. Biomech.* **2015**, *31*, 423–429. https://doi.org/10.1123/jab.2014-0159.

### 2.49. Effect of 6 Weeks of Taekwondo Training on Athletes’ Neuromuscular Performance

**Amanda Ribeiro ^1^, Larissa Faria ^1^, Márcio Júnior ^1,2^, Fabiano Fonseca ^3^ and Maicon Albuquerque ^1^**

^1^ Combat Sports Laboratory, Department of Sports, Federal University of Minas Gerais, Brazil; aribeiroufmg@gmail.com (A.R.); lof.ufv@gmail.com (L.F.); marciovmjr@gmail.com (M.J.); lin.maicon@gmail.com (M.A.) 

^2^ President Antônio Carlos University (UNIPAC)—Barbacena/Minas Gerais 

^3^ Postgraduate Program in Physical Education, Federal University of Pernambuco, Recife, Pernambuco, Brazil; dr.fsfonseca@gmail.com (F.F.) 

Taekwondo (TKD) performance is influenced by the athlete’s ability to generate muscle power [1]. The countermovement jump test (CMJ) has been used to evaluate the neuromuscular performance of lower limbs [2]. The efficiency of the neuromuscular system in producing ballistic actions of lower limbs with high power can discriminate the performance level of athletes [1]. Few studies have investigated the effect of technical-tactical TKD training combined with strength training on neuromuscular performance [3]. So, the present study aimed to analyze the effect of 6 weeks of technical-tactical TKD combined with strength training on athletes’ neuromuscular performance. Fourteen TKD athletes participated in training sessions; however, five were excluded because they enrolled in less than 70% of sessions. Therefore, the final sample was composed of nine national and international level athletes (seven men; mean ± SD, age: 17.44 ± 3.26 years). Athletes performed 6 weeks of training, with the intensity of each session prescribed based on the coach’s rating of perceived exertion (${\mathrm{RPE}}_{\mathrm{coach}})$, in which weeks 1 (${\mathrm{RPE}}_{\mathrm{coach}}$ = 6.85 ± 3.31), 2 (${\mathrm{RPE}}_{\mathrm{coach}}$ = 6.85 ± 3.31), and 4 (${\mathrm{RPE}}_{\mathrm{coach}}$ = 6.85 ± 3.31) were prescribed to be harder than weeks 3 (${\mathrm{RPE}}_{\mathrm{coach}}$ = 2.00 ± 0.02), 5 (${\mathrm{RPE}}_{\mathrm{coach}}$ = 3.85 ± 2.29), and 6 (${\mathrm{RPE}}_{\mathrm{coach}}$ = 2.71±3.53). Strength training happened three times per week for 1 h, and consisted of three sets of 6–8 repetitions of predetermined exercises, at 60 to 75% of 1RM, followed by six repetitions of plyometric exercises, with a 2 min interval between sets, whereas TKD training occurred 5 to 6 days a week lasting 60 to 90 min. TKD training was composed of combat simulations, and technical-tactical exercises using kick pads and shields. The CMJ was performed at the beginning of week 1 (pre-intervention test) and at the end of week 6 (post-intervention test). Analyses were conducted using α = 5% and performed using RStudio. The data were normally distributed and the paired *t*-student test was used. Cohen’s d was used to calculate the effect sizes. The results showed that there was an improvement in CMJ performance (*t* = −3.0832, df = 8, *p*-value = 0.015) from pre-test [CMJ (cm) = 33.89 ± 8.58] to post [CMJ (cm) = 40.04 ± 6.74] with a medium effect size (d = −0.77). Thus, we concluded that 6 weeks of technical-tactical TKD combined with strength training increased the neuromuscular performance of athletes.

**Funding:** This study was financed by the Emenda Parlamentar awarded by the Deputado Federal Luis Tibé (no.: 27620007). Also, this study was financed in part by Coordenação de Aperfeiçoamento de Pessoal de Nível Superior -Brasil (CAPES)-Finance Code 001 and by Fundação de Amparo à Pesquisa do Estado de Minas Gerais -Brazil (FAPEMIG).

**References**

1. Albuquerque, M.R.; Tavares, L.D.; Longo, A.R.; Mesquita, P.H.C.; Franchini, E. Relationship between indirect measures of aerobic and muscle power with frequency speed of kick test multiple performance in taekwondo athletes. *Int. J. Sports Med.* **2022**, *43*, 254–261. https://doi.org/10.1055/a-1546-9221.
2. Claudino, J.; Cronin, J.; Mezêncio, B.; McMaster, D.; McGuigan, M.; Tricoli, V.; et al. The countermovement jump to monitor neuromuscular status: A meta-analysis. *J. Sci. Med. Sport* **2016**, *20*, 397–402. https://doi.org/10.1016/j.jsams.2016.08.011.
3. Santos, J.; Herrera-Valenzuela, T.; Franchini, E. Can different conditioning activities and rest intervals affect the acute performance of taekwondo turning kick? *J. Strength Cond. Res.* **2015**, *29*, 1640–1647. https://doi.org/10.1519/JSC.0000000000000808.

### 2.50. Effect of Exercise, Vitamin D and Calcium on Bone Mineral Density in Elderly People: A Systematic Review of Randomized Controlled Trials

**Liz Graciela Sanabria Rivarola ^1^, Lucas de Souza Campos Santos ^2^ and Ricardo Fernandes ^3^**

^1^ Programa de Pós-Graduação, Faculdade de Ciências da Saúde, Universidade Federal da Grande Dourados (UFGD); liz.graciela.sanabria@gmail.com (L.G.S.R.) 

^2^ Faculdade de Ciências da Saúde, Universidade Federal da Grande Dourados (UFGD); lucasouacampos@hotmail.com (L.S.C.S.)

^3^ Programa de Pós-Graduação em Alimentos, Nutrição e Saúde, Faculdade de Ciências da Saúde, Universidade Federal da Grande Dourados (UFGD); ricardofernandes@ufgd.edu.br (R.F.) 

Aging has degenerative effects on musculoskeletal, cardiovascular and central nervous system structures. Associated with this, physical inactivity causes a marked decline in muscle mass and bone mass [1]. The aim of this systematic review was to summarize the body of evidence on the relationship of exercise combined with oral vitamin D and/or calcium supplementation in increasing bone mineral density (BMD) in men and women aged 50 years and older. The review and meta-analysis were conducted according to the Cochrane Handbook for Systematic Reviews of Interventions [2], and registered in the International Prospective Register of Systematic Reviews (PROSPERO) under registration number: CRD42021237473. The comprehensive search was performed in the electronic databases MEDLINE/PubMed, Web of Science, Scopus and Cochrane Central Register of Controlled Trials (CENTRAL) until March 2021 and updated in September 2021. The PICO search strategy was used. Eligible studies were randomized clinical trials including men and women (aged ≥ 50 years) in all contexts: institutionalized, communities, hospitals, and others; enlisting supervised exercise interventions and vitamin D and/or calcium supplementation provided to patients during the study; including outcomes of BMD of the lumbar spine, femoral neck or hip; or serum vitamin D/calcium status comparing results with a control group with supplementation, sedentary or low-intensity unsupervised physical activity. The literature search identified 4621 results distributed in MEDLINE/PubMed databases: 944; Web of Science: 1656; Scopus: 1339 and Cochrane Central Register of Controlled Trials (CENTRAL): 682. After selection and application of inclusion and exclusion criteria, 15 studies were included in the systematic review and 10 studies in the meta-analysis. To assess the risk of bias in the studies, the Cochrane RoB (Risk of Bias) 2.0 tool was used. The studies included 1327 participants with a mean age of 66.9 years. Three studies included men and women and twelve studies included only women. The results showed a significant effect on lumbar spine BMD in the exercise and vitamin D plus calcium when compared to the vitamin D plus calcium. Furthermore, there was a significant effect on femoral neck and hip BMD in the exercise and calcium supplementation group vs. calcium alone, supporting studies with high-intensity exercise intervention. This study has some limitations such as the number of studies included in the meta-analysis, which suggests that attention should be paid to the results. Although modest, exercise associated with vitamin D and/or calcium supplementation showed significant results for BMD of the lumbar spine, femoral neck and hip.

**Funding:** The author LGSR was funded by Foundation for Support to the Development of Education, Science and Technology of the State of Mato Grosso do Sul (FUNDECT), number 17/2019.

**References**

1. Seene, T.; Kaasik, P.; Riso, E.M. Review on aging, unloading and reloading: changes in skeletal muscle quantity and quality. *Arch. Gerontol. Geriatr.* **2012**, *54*, 374–380. https://doi.org/10.1016/J.ARCHGER.2011.05.002.
2. Higgins, J.P.T.; Thomas, J.; Chandler, J.; Cumpston, M.; Li, T.; Page, M.J.; Welch, V.A. Eds. *Cochrane Handbook for Systematic Reviews of Interventions* version 6.3 (updated February 2022). Cochrane, 2022. Available online: http://www.training.cochrane.org/handbook (accessed on 24 April 2022).

### 2.51. Internal Load Analysis after High-Intensity Interval Training with Fixed and Self-Selected Recovery Time

Leandro Sant’Ana ^1,^*, Diogo Monteiro ^2^ and Jeferson Vianna ^1^

^1^ Post Graduate Program in Physical Education, Federal University of Juiz de Fora, Minas Gerais, Brazil; jeferson.vianna@ufjf.edu.br (J.V.) 

^2^ ESECS, Polytechnic of Leiria, Leiria, Portugal 2; diogo.monteiro@ipleiria.pt (D.M.)

* Correspondence: leandrosantana.edufisica@hotmail.com (L.S.)

This study analyzed the internal load in high-intensity interval training sessions with different conditions of recovery time between stimuli: fixed (1 min) and self-selected. Nineteen individuals participated in the study: 13 men and 6 women (19 ± 1.0 years; 64.0 ± 9.2 kg; 169 ± 8.5 cm; 22.0 ± 2 BMI). For the internal load analysis, heart rate variability (LnRMSSD) [1], perceived effort (PE) [2] and mood scale-BRUMS (MS) [3] were used. LnRMSSD and MS were evaluated before and after the sessions. The PE was evaluated during each session, immediately after each stimulus. The protocol was 10 × 30s (95% Vpeak) with active recovery (40% Vpeak) of fixed or self-selected duration. ANOVA-RM (2 [interventions] × 2 [time points]) for LnRMSSD and MS and (2 [interventions] × 10 [time points]) for PE was used. Between condition and time*condition, no differences were observed for LnRMSSD (*p* = 0.626; *p* = 0.879, respectively), PE (*p* = 0.191; *p* = 0.792, respectively), and MS (tension: *p* = 0.673; *p* = 0.463; depression: *p* = 0.867; *p* = 0.359; anger: *p* = 0.867; *p* = 0.359; vigor: *p* = 0.811; *p* = 0.778; fatigue: *p* = 0.144; *p* = 0.998; mental confusion: p = 0.828; *p* = 0.752, respectively). In terms of time, significant differences were observed in LnRMSSD (*p* < 0.001) and PE (1 ≠ 3–10; 2 ≠ 4–10; 3 ≠ 5–10; 4 ≠ 5–10; 5 ≠ 7–10; 6 ≠ 7–10; 7 ≠ 9,10; 8 ≠ 10, *p* < 0.001). In MS, differences were found in domains of tension (*p* < 0.001), depression (*p* < 0.015), anger (*p* < 0.033), and mental confusion (*p* < 0.001) but not for vigor (*p* = 0.339) and fatigue (*p* = 0.419) which are associated with internal load. However, both recovery conditions showed similar acute internal load responses. Additionally, it is suggested that recovery with self-selected time (46.70 ± 1.6.58s) may be a suitable recovery option in HIIT prescription.

**Funding:** This research received no external funding.

**References**

1. Schmitt, L.; Regnard, J.; Millet, G.P. Monitoring Fatigue Status with HRV Measures in Elite Athletes: An Avenue beyond RMSSD? *Front. Physiol.* **2015**, *6*, 1–3. https://doi.org/10.3389/fphys.2015.00343.
2. Utter, A.C.; Robertson, R.J.; Green, J.M.; Suminski, R.R.; McAnulty, S.R.; Nieman, D.C. Validation of the Adult OMNI Scale of Perceived Exertion for Walking/Running Exercise. *Med. Sci. Sports Exerc.* **2004**, *36*, 1776–1780. https://doi.org/10.1249/01.MSS.0000142310.97274.94.
3. Morgan, W.P.; Brown, D.R.; Raglin, J.S.; O’Connor, P.J.; Ellickson, K.A. Psychological Monitoring of Overtraining and Staleness. *Brit. J. Sport Med.* **1987**, *21*, 107–114. https://doi.org/10.1136/bjsm.21.3.107.

### 2.52. Bibliometric Analysis of Brazilian Studies About Female Soccer Published between 2010 and 2020

Ignácio A. Seixas-da-Silva ^1,2,3,^*, Victor A. Principe ^2,3,4^ and Rodolfo de Alkmim M. Nunes ^3,4^

^1^ UNESA Research Productivity Program Scholarship, Estácio de Sá University, Rio de Janeiro, Brazil

^2^ Estácio de Sá University, Cabo Frio, Brazil; vitor.principe@estacio.br (V.A.P.)

^3^ Laboratory of Exercise and Sports (LABEES), Rio de Janeiro State University, Brazil; rodolfoalkmim@gmail.com (R.A.M.N.) 

^4^ Physical Education and Sports Institute, Rio de Janeiro State University, Rio de Janeiro, Brazil

* Correspondence: ignacio.silva@estacio.br (I.A.S.S.)

Bibliometric analysis in sports is an important tool to evaluate the quality of studies and comprehensive mapping analysis can identify the areas of greater interest and potential new studies [1,2]. This study aimed to identify the Brazilian publications about female soccer between 2010 and 2020. The searches were conducted on Pubmed, SportDiscus, Scopus and ISI Web of Science. The final search phrase was (Football* OR Soccer*) AND (Woman* OR female*). Python 3.6 was used to analyze the results found. It is possible to verify, in all Brazilian studies published, the main author; the study profile; the sports science area; the instruments used; and the results found, respectively: Ramos, Guilherme; Locomotor activity profiles; Technology; GPS Tracking; Development of specific methodologies for soccer training. Pavin, Larissa; Muscle fatigue; Sports Training and Exercise Physiology (STEP); Compression stockings and physical tests; Compression stockings can improve physical performance after the match. Loturco, Irineu; Physical Performance; STEP; Physical Tests; Players with better physical performances present superior sports performances. Dias, Rodrigo; Pre-season performance; STEP; Physical Tests; Improvement of physical abilities. Ferreti, Marco; Media; Psychology and Social; Gender difference in sports media; Little space in the sports media for women’s soccer. Gonçalves, Livia; Food intake and dietary; STEP; Glycemic index, glycemic load, and 24 h dietary recalls; Athletes’ diets were inadequate. Morais, André; Cardiac hypertrophy differs between genders in professional soccer; STEP; Image Evaluation; The existing changes are due to the body differences. Nicolao, Ana; Maturity status and lactate tthreshold; STEP; Physical tests; No correlation between lactate threshold and maturational level; Andrade, Marília; Muscular strength; Injury and Recovery (IR); Isokinetic strength evaluations; Injury prevention programs in young players. Silva Neto, Moacir; Muscular strength; STEP; Isokinetic strength evaluations; Proposal of references to assist in the prescription of training. Vargas, Valentine; Muscular strength; STEP; Isokinetic strength evaluations; The best development of muscle strength happens at U-13 and U-17. Andaku, Daniela; Cardiovascular function and long-term effects of exercise training; STEP; Ultrasound and physical test; Robust brachial artery at rest in elite female players. Andrade, Marília; Evaluation of test–retest reliability; IR; Isokinetic strength evaluations; Test–retest reliability seems to be affected by the type of muscle and test velocity. Ribas, Letícia; Proprioception and muscle strength; IR; Physical Test; Physical training can prevent injury. The Brazilian scientific literature has a scarcity of material in the areas of injuries, technology, and social aspects, these being the main gaps to be filled by future studies in female soccer.

**Funding:** This research received no external funding.

**References**

1. Brito, J.; Nassis, G.P.; Seabra, A.T.; Figueiredo, P. Top 50 most-cited articles in medicine and science in football. *BMJ Open Sport Exerc. Med.* **2018**, *4*, e000388. https://doi.org/10.1136/bmjsem-2018-000388.
2. Coimbra, D.R.; Dominski, F.H.; Brandt, R.; Bevilacqua, G.G.; Andreato, L.V.; Andrade, A. Twenty Years of Scientific Production in Sport and Exercise Psychology Journals: A Bibliometric Analysis in Web of Science. *J. Sports Psychol.* **2022**, *31*, 245–261.

### 2.53. Effect of 3 Weeks of Home-Based Lockdown Training on Change of Direction in Young Basketball Athletes

**Allan Serafim**

Universidade Metodista de São Paulo; allan.serafim1@metodista.br (A.S.) 

The COVID-19 pandemic caused basketball athletes to have their training routines adjusted, such as including training in a home setting during lockdown [1,2,3]. In basketball, it is important to improve physical capacities such as change of direction [2]. Therefore, the aim of this study was to evaluate the effects of 3 weeks of home-based training (lockdown) on the change of direction of young basketball athletes. We evaluated 10 basketball athletes (age 14 ± 6 years, height (cm) 160 ± 25; body mass (kg) 60 ± 12) from an amateur team from São Paulo, Brazil. The training protocol (5 min warm-up + 25 min body weight exercises: air squat, push up, lunge, isometric plank and single leg deadlift) was proposed three times a week on alternate days via video call. The change of direction was evaluated before and after lockdown, using the modified T40 test with frontal, lateral and backward displacement. A descriptive analysis of the data and Student’s *t* test were used to compare means. The analysis was conducted using SPSS 20 software with a significance level of *p* < 0.05. The results reported that there was an improvement in the athletes’ change of direction (Pre 9.2 ± 0.29 s vs. Post 8.9 ± 0.33 s, ES = 0.96); however, without statistical significance. It is considered that 30 min of home-based training, performed three times a week on alternate days, with eccentric exercises, can be enough to at least sustain the physical capacity of change of direction in young basketball athletes.

**Funding:** This research received no external funding.

**References**

1. Zu, Z.Y.; Di Jiang, M. Coronavirus disease 2019 (COVID-19): a perspective from China. *Radiology* **2020**, 296. 15–25. https://doi.org/10.1148/radiol.2020200490.
2. Luna, B.; Chiner, P.M. Cambios en fuerza explosiva y agilidad tras un entrenamiento online en jóvenes jugadores de baloncesto confinados por COVID-19. *Retos Nuevas Tend. Educ. Física Deporte Recreación* **2021**, *41*, 256–264. https://doi.org/10.47197/retos.v0i41.83011.
3. Stojanovic, E.; Stojiljković, N. The activity demands and physiological responses encountered during basketball match-play: A systematic review. *Sport. Med.* **2018**, *48,* 111–135. https://doi.org/10.1007/s40279-017-0794-z.

### 2.54. Symmetry of Lower Limb Power in Vertical Jump Performance

Yuri Silva ^1,2,^*, Giullio da Silva ^1,2^, Larissa Bastos ^3^, Andressa dos Santos ^1,2^, Henri Lima ^3^, Dayane Costa ^3^, Vicente Lima ^1,2,3^ and Rodolfo Nunes ^1,2^

1 Institute of Physical Education and Sports (IEFD), Graduate Program in Exercise and Sports Science (PPGCEE), State University of Rio de Janeiro (UERJ); Rio de Janeiro-RJ, Brazil; giulliocesar.gc@hotmail.com (G.S.); professoraoliveira.andressa@gmail.com (A.S.); professorvicentelima@gmail.com (V.L.); rodolfoalkmim@gmail.com (R.N.)

^2^ Laboratory of Exercise and Sport (LABEES), State University of Rio de Janeiro (UERJ); Rio de Janeiro, RJ, Brazil

^3^ Biodynamics of Performance, Exercise and Health Research Group, State University of Rio de Janeiro (UERJ); Rio de Janeiro-RJ, Brazil; larissaruizbastoss@gmail.com (L.B.); henrilima@gmail.com (H.L.); dayane.costamarins10@gmail.com (D.C.)

* Correspondence: professoryurirolim@gmail.com (Y.S.)

It is estimated that among men and women, about 265 million people around the world play football. Of these, the female audience has increased considerably in recent years [1]. The practice of soccer requires specific efforts from athletes that can generate muscle imbalances that accentuate asymmetries between the limbs [2]. For the above, the present study aims to correlate the values of the differences between the performances in the triple hop test with the results of the vertical jump tests in young female soccer athletes. The sample consisted of eight female soccer athletes, from a team of the west of the city of Rio de Janeiro, 21.63 ± 2.97 years old, 55.80 ± 5.49 kg of total body mass (TBM) and 161.88 ± 7.06 cm in height (HGT). Athletes completed a testing battery in two different visits to the training center. On the first day, total body mass and height were registered, and countermovement vertical jump with (CMJ) and without arms (ABALAKOV) and squat jump (SJ) were performed. Jump data were collected using a device that demonstrates valid and reliable conditions for measuring jump height (*Vert*). *Vert* device was placed at the height of the iliac crest, close to the superior edge of the sacrum and kept fixed with an elastic tape [3]. On the second day, triple hop test (HT) and right and left lower limb height tests (RHT and LHT, respectively) were performed. This test was performed with three attempts of three consecutive maximal hops forward on the same limb, with the performance measured by the distance in meters from the starting position [4]. Asymmetry between limbs was quantified as the difference in performance between limbs (asymmetry = best jump − worse jump) of the HT. For statistical analysis, the Pearson Correlation Test was performed and the results showed a strong negative correlation between HT asymmetry with SJ and ABALAKOV height (r = −0.754; *p* = 0.031) and (r = −0.930; *p* = 0.001), respectively. In conclusion, individuals with more symmetrical TH performance achieved better performance in the vertical jump. Thus, it can be concluded that minimizing asymmetry between limbs improves performance in the vertical jump. However, it is not possible to extrapolate the results of the present study, considering the current sample size. The present investigation is in progress and will soon have a larger size, new variables and different categories of the modality.

**Funding:** This research received no external funding.

**References**

1. Pappalardo, L.; Rossi, A.; Natilli, M.; Cintia, P. Explaining the difference between men’s and women’s football. *Plos ONE* **2021**, *16*, 1–17. https://doi.org/10.1371/journal.pone.0255407.
2. Hart, N.H.; Nimphius, S.; Weber, J.; Spiteri, T.; Rantalainen, T.; Dobbin, M.; Newton, R.U. Musculoskeletal asymmetry in football athletes: A product of limb function over time. *Med. Sci. Sports Exerc.* **2016**, *48*, 1379–1387. https://doi.org/10.1249/MSS.0000000000000897.
3. Borges, T.O.; Moreira, A.; Bacchi, R.; Finotti, R.L.; Ramos, M.; Lopes, C.R.; Aoki, M.S. Validation of VERT wearable jump monitor device in elite youth volleyball players. *Biol. Sport* **2017**, *34*, 239–247. https://doi.org/10.5114/biolsport.2017.66000.
4. Hamilton, R.T.; Shultz, S.J.; Schimitz, R.J.; Perrin, D.H. Triple-Hop Distance as a Valid Predictor of Lower Limb Strength and Power. *J. Athl. Train.* **2008,** *43*, 144–151. https://doi.org/10.4085/1062-6050-43.2.144.

### 2.55. Vertical Jump Test Performance, Eccentric Ratio and Arm Swing Effect in Elite Brazilian Swimmers

**Geovani Silva, Jerusa Lara, Luiz Zanelato, Fernando Vanzella and Cesar Abad**

Reference Center of Sport Science of Service of Social Industry (CRCE-SESI); geovaniveridiano@outlook.com (G.S.); jeura.laura@sesisp.org.br (J.L.); luiz.zanelato@sesisp.org.br (L.Z.); vanzella@sesisp.org.br (F.V.); cesar.abad@sesisp.org.br (C.A.)

The squat jump (SJ) and the countermovement jump (CMJ) are two common vertical jump tests (VJs) used to assess lower limb muscle power in many sports [1]. By the CMJ:SJ ratio, it is possible to assess the eccentric utilization ratio (EUR), which serves as an indicator of performance [2]. CMJ with arms swing (CMJa) is another jump test applied in many athletes because it is a more natural and functional approach that allows an increase in jumping performance [3]. The performance of SJ, CMJ and CMJa is well described in land sports, but is not completely understood in swimmers. This knowledge may help to better comprehend the concentric and eccentric muscle actions of swimmers, which are correlated with start swim, turns, and overall swimming performance [4]. The aims of the present study were (i) to compare the jump height of SJ, CMJ and CMJa in sprint swimmers; (ii) to introduce the arm swing effect (ASE) calculated by the CMJa:CMJ ratio; and (iii) to verify the relationship among SJ, CMJ, CMJa, EUR and ASE. Fifteen elite (seven male and four female) junior and senior elite Brazilian sprint swimmers (age male 20 ± 2.98, female 19 ± 5.48) volunteered to take part in the study. All VJs were performed in a single session during the starting season. Significant differences were found among all VJs (SJ = 45.4 ± 7.1 cm; CMJ = 48.6 ± 7.4 cm; CMJa = 55.4 ± 8.9 cm; *p* < 0.001). EUR was 3.3 ± 2.4 cm (7.4 ± 5.8%), ASE was 6.7 ± 3.0 cm (13.8 ± 6.0%), and CMJa was 10.0 ± 3.2 cm (22.1 ± 6.3%) greater than SJ. A near perfect association was found among all VJ tests (SJ × CMJ r = 0.94; CMJ × CMJa r = 0.95; SJ × CMJa r = 0.95). EUR showed a negative trivial correlation with SJ (r = −0.04), a positive small association with CMJa r = 0.13) and a positive moderate relationship with CMJ (r = 0.30). The correlation found for ASE was positive moderate with SJ and CMJ (r = 0.50; r = 0.37), negative moderate with EUR (r = −0.33), and positive large with CMJa (r = 0.64). In conclusion, 89% of any VJ performance can be explained by the jump height found in any other VJ test. SJ, CMJ and CMJa are able to explain 25, 13 and 41% of the ASE, respectively, which had an inverse correlation with EUR. These results suggest that an arm swing executed during the CMJ elicits an additional coordinative function that seems to change both concentric and eccentric muscle actions related to SJ and CMJ performance of sprint swimmers.

**Funding:** This research received no external funding.

**References**

1. Kozinc, Ž.; Žitnik, J.; Smajla, D.; Šarabon, N. The difference between squat jump and countermovement jump in 770 male and female participants from different sports. *Eur. J. Sport Sci.* **2022**, *22*, 985–993. https://doi.org/10.1080/17461391.2021.1936654.
2. Mcguigan, M.R.; Doyle, T.L.; Newton, M.; Edwards, D.J.; Nimphius, S.; Newton, R.U. Eccentric utilization ratio: effect of sport and phase of training. *J. Strength Cond. Res.* **2003***, 20*, 992–995. https://doi.org/10.1519/R-19165.1.
3. Laffaye, G.; Wagner, P.P.; Tombleson, T.I. Countermovement jump height: Gender and sport-specific differences in the force‒time variables. *J. Strength Cond. Res.* **2014**, *28*, 1096–1105. https://doi.org/10.1519/JSC.0b013e3182a1db03.
4. Keiner, M.; Wirth, K.; Fuhrmann, S.; Kunz, M.; Hartmann, H.; Haff, G.G. The influence of upper-and lower-body maximum strength on swim block start, turn, and overall swim performance in sprint swimming. *J. Strength Cond. Res.* **2022**, *35*, 2839-2845. https://doi.org/10.1519/JSC.0000000000003229.

### 2.56. CNTF as a Potential Biomarker of Post-COVID Muscle Performance

**Matheus Sinhorelli Cioccari, Rafael Lopes Rosa, Walter Orlando Beys-da-Silva, Larissa Daniele Bobermin and André Quincozes Santos**

Federal University of Rio Grande do Sul; mathcioccari@gmail.com (M.S.C.); rafaelbiotec@gmail.com (R.L.R.); walter.beys@ufrgs.br (W.O.B.S.); llarissa.bobermin@ufrgs.br (L.D.B.); andrequincozes@ufrgs.br (A.Q.S.) 

Ciliary neurotrophic factor (CNTF) is a member of the interleukin 6 (IL-6) family highly expressed in neural cells and skeletal muscle. Alterations in muscle function may impact the central nervous system response; additionally, neuromuscular adaptations and synaptic plasticity are closely associated with the muscle performance [1]. Although most studies aim to determine the relation between exercise and neurons, it is important to note that glial cells, particularly astrocytes, are extremely versatile and can be positively modulated by exercise training. Moreover, these cells are directly associated with synaptic processing, neural plasticity, and excitability, as well as trophic and metabolic support [2,3] and CNTF is highly expressed in astrocytes. Regarding the role of CNTF, it can prevent motor neuron degeneration through the modulation of myotrophic factor related to the PI3K/Akt (phosphatidylinositol 3-kinase/protein kinase B) and JAK/STAT (Janus kinase/signal transducer and activator of transcription) signaling pathways in skeletal muscle [4]. In line with this, we believe that CNTF may act as a potential predictive marker of muscle performance, because the PI3K/Akt and JAK/STAT are directly related to increases in strength and muscle hypertrophy. In addition, we highlight that IL-6 is strongly associated with cytokine storm in COVID-19 infection, which changes plasticity, cognitive, sensorial, and muscle function [5]. It is noteworthy that IL-6 is an unspecific marker of neural degeneration and consequently muscle function, but CNTF can emerge as a potential biomarker of post-COVID muscle performance. Therefore, bioinformatic analyses can correlate the potential effect of CNTF on long COVID-19 and/or post-COVID-19 conditions. It may be speculated that neuromuscular function is also correlated with astrocytes, which then can improve exercise performance. The evaluation of CNTF can be performed easily from blood or saliva and can predict neuromuscular/astrocyte damage, as well as avoid the decrease in the number of competing athletes. In summary, we point out that due to long COVID-19 and its impacts on the brain and neuromuscular function, in a few years, there may be a smaller number of high performance athletes participating in training.

**Funding:** This research received no external funding.

**References**

1. Park, I.S.; Lee, K.J.; Han, J.W.; Lee, N.J.; Lee, W.T.; Park, K.A., Rhyu, I.J. Basketball training increases striatum volume. *Hum. Mov. Sci.* **2011**, *30*, 56–62. https://doi.org/10.1016/j.humov.2010.09.001.
2. Sancho, L.; Contreras, M.; Allen, N.J. Glia as sculptors of synaptic plasticity. *Neurosci. Res.* **2021**, *167*, 17–29. https://doi.org/10.1016/j.neures.2020.11.005.
3. Quincozes-Santos, A.; Santos, C.L.; de Souza Almeida, R.R.; da Silva, A.; Thomaz, N.K.; Costa, N.; Weber, F.B.; Schmitz, I.; Medeiros, L.S.; Medeiros, L.; Dotto, B.S.; Dias, F.; Sovrani, V.; Bobermin, L.D. Gliotoxicity and Glioprotection: the Dual Role of Glial Cells. *Mol. Neurobiol.* **2021**, *58*, 6577–6592. https://doi.org/10.1007/s12035-021-02574-9.
4. Hiatt, K.; Lewis, D.; Shew, M.; Bijangi-Vishehsaraei, K.; Halum, S. Ciliary neurotrophic factor (CNTF) promotes skeletal muscle progenitor cell (MPC) viability via the phosphatidylinositol 3-kinase–Akt pathway. *J. Tissue Eng. Regen. Med.* **2012**, *8*, 963–968. https://doi.org/10.1002/term.1598.
5. Douaud, G.; Lee, S.; Alfaro-Almagro, F.; Arthofer, C.; Wang, C.; McCarthy, P.; Lange, F.; Andersson, J.; Griffanti, L.; Duff, E.; Jbabdi, S.; Taschler, B.; Keating, P.; Winkler, A.M.; Collins, R.; Matthews, P.M.; Allen, N.; Miller, K.L.; Nichols, T.E.; Smith, S.M. Sars-Cov-2 is associated with changes in brain structure in UK Biobank. *Nature* **2022**, *604*, 697–707. https://doi.org/10.1038/s41586-022-04569-5.

### 2.57. Molecular Alterations Related to Muscle Performance and Brain Functions

**Matheus Sinhorelli Cioccari, Yorran Hardman Araujo Montenegro, Larissa Daniele Bobermin and André Quincozes-Santos**

Federal University of Rio Grande do Sul; mathcioccari@gmail.com (M.S.C.); yorran_montenegro@hotmail.com (Y.H.A.M.); larissa.bobermin@ufrgs.br (L.D.B.); andrequincozes@ufrgs.br (A.Q.S.) 

Molecular biomarkers associated with muscle performance have increasingly interested scientists/coaches who seek to improve the performance of athletes, which is also closely associated with central nervous system (CNS) function. Therefore, our study sought to identify molecular alterations (genes and/or proteins) related to muscle performance and brain adaptations, using the GWAS (Genone Wide Association Studies) Catalog. The markers RIT2 (Ras Like Without CAAX 2), SYT4 (Synaptotagmin 4), PML (PML Nuclear Body Scaffold), MEF2C (Myocyte Enhancer Factor 2C), and CIR1 (Corepressor Interacting With RBPJ) were found linking muscle and brain. An interactome between these genes was made by The Human Reference Protein Interactome Mapping Project (HuRI) and the association between them resulted in 76 interactions and 42 different protein sets, which may be predictive biomarkers of muscle performance. These proteins are associated with cellular processes and metabolic functions, being 94% regulatory, 88% involved in amino acid metabolism, and 75% related to gene expression protein translation. In addition, regarding brain regions, striatum and cerebellum have a direct relationship with the control of strength and physical movement, as well as motor and sensory functions [1,2]. In this regard, increased striatum volume has been determined as one of the key distinguishing characteristics in high-level athletes [3], and we found increased molecular expression of SYT4 and RIT2 for both striatum and cerebellum. SYT4 coordinates several functions including control of synaptic activity, such as glutamatergic synapse and calcium homeostasis. It is noteworthy that both functions are fundamental for the plasticity of the CNS with direct impact on muscle strength and conditioning. RIT2 can modulate energy metabolism and interacts with the dopaminergic synapse, fundamental in the reward mechanism, and consequently, in high muscle performance. Furthermore, MEF2 plays a role in cellular differentiation, including muscle cells; CIR1 is directly linked with protein functions and histone deacetylase binding activity; and PML is related to circadian regulation of gene expression, as well as cell cycle functions and DNA repair. Finally, it is important to consider that the evaluation of these molecular markers may be easily available through saliva and/or blood collection and can represent potential and innovative molecular markers to predict high muscle performance, particularly associated with brain functions/adaptations.

**Funding:** This research received no external funding.

**References**

1. Taubert, M.; Wenzel, U.; Draganski, B.; Kiebel, S.J.; Ragert, P.; Krug, J.; Villringer, A. Investigating Neuroanatomical Features in Top Athletes at the Single Subject Level. *Plos ONE* **2015**, *10*, e0129508. https://doi.org/10.1371/journal.pone.0129508.
2. Peterburs, J.; Desmond, J.E. The role of the human cerebellum in performance monitoring. *Curr. Opin. Neurobiol.* **2016**, *40*, 38–44. https://doi.org/10.1016/j.conb.2016.06.011.
3. Park, I.S.; Lee, K.J.; Han, J.W.; Lee, N.J.; Lee, W.T.; Park, K.A.; Rhyu, I.J. Basketball training increases striatum volume. *Hum. Mov. Sci.* **2011**, *30*, 56–62. https://doi.org/10.1016/j.humov.2010.09.001.

### 2.58. Correlation between Two Assessment Devices during the Optimum Power Load Test

Paolo Sirieiro, Igor Nasser and Humberto Miranda *

Universidade Federal do Rio de Janeiro; paolosirieiro@ufrj.br (P.S.); igor_nasser@hotmail.com (I.N.)

* Correspondence: humbertomirandaufrj@gmail.com (H.M.)

The squat jump exercise has been widely used as a power exercise in several sports, and training it at optimum power load (OPL) seems to be an effective and practical way to maximize the muscle power output [1]. In this sense, alternatives and cheap tools are recommended as practical applications of researchers and coaches. The aim of this study was to compare two technological assessment instruments during the OPL test: a linear encoder (Peak Power, Cefise, SP, Brazil) and a previously validated and reliable [2] wearable wireless device (WWD) (Beast Sensor, Milan, Italy). Fifteen competitive athletes from different disciplines (aquatics: n = 7, lands: n = 8), (age: 18.67 ± 2.99 year; body mass (BM): 70.93 ± 8.41 kg, height: 181 ± 0.09 cm) took part in this study. The protocol test was the same as that used by Loturco et al., [3] and was performed on a Smith-machine device (Matrix Fitness, Cottage Grove, WI, USA). The test started at a load of 40% of the athletes’ BM, and a load of 10% of BM was gradually added until a decrement in the bar peak power (PP) was observed by linear encoder and recorded by the WWD coupled on the bar simultaneously. A 5 min interval was provided between sets. Pearson’s correlation analysis between devices on BM at the PP moment and PP at each BM’s percentual were checked. Furthermore, the paired *t*-test between PP with OPL was examined. The statistical significance level at *p* < 0.05 was used for all tests. Nearly perfect correlation was found at all loads corresponding to 40–110% athletes’ BM (r = 0.99; *p* = 0.00) and at BM on PP moment (BM%: 110 ± 18.89 to WWD and 112 ± 16.56 to encoder; r = 0.94; *p* = 0.00). Furthermore, the paired *t*-test showed no meaningful difference at PP with OPL (PP = 1737.86 ± 479.47 w to encoder and 1696.13 ± 499.5 w to WWD; *p* = 0.08). As presented, a nearly perfect association was found between the OPL obtained with the WWD and the one attained with the encoder. Moreover, similar power output levels were found between devices irrespective of the BM load. Interestingly, as previously documented [4] the athletes’ OPL was also achieved at a mean propulsive velocity close to 1.0 m·s^−1^ (0.98 ± 0.08) by WWD. These findings are important to practitioners and for sports coaches to be able to conduct the OPL test at low cost and with a practical and reliable WWD.

**Funding:** This research received no external funding.

**References**

1. Soriano, M.A.; Jiménez-Reyes, P.; Rhea, M.R.; Marín, P.J. The Optimal Load for Maximal Power Production During Lower-Body Resistance Exercises: A Meta-Analysis. *Sports Med. (Auckland, N.Z.)* **2015**, *45*, 1191–1205. https://doi.org/10.1007/s40279-015-0341-8.
2. Balsalobre-Fernández, C.; Marchante, D.; Baz-Valle, E.; Alonso-Molero, I.; Jiménez, S.L.; Muñóz-López, M. Analysis of Wearable and Smartphone-Based Technologies for the Measurement of Barbell Velocity in Different Resistance Training Exercises. *Front. Physiol.* **2017**, *8*, 649. https://doi.org/10.3389/fphys.2017.00649.
3. Loturco, I.; McGuigan, M.R.; Freitas, T.T.; Valenzuela, P.L.; Pereira, L.A.; Pareja-Blanco, F. Performance and reference data in the jump squat at different relative loads in elite sprinters, rugby players, and soccer players. *Biol. Sport* **2021**, *38*, 219–227. https://doi.org/10.5114/biolsport.2020.98452.
4. Loturco, I.; Nakamura, F.Y.; Tricoli, V.; Kobal, R.; Cal Abad, C.C.; Kitamura, K.; Ugrinowitsch, C.; Gil, S.; Pereira, L.A.; González-Badillo, J.J. Determining the Optimum Power Load in Jump Squat Using the Mean Propulsive Velocity. *PloS ONE* **2015**, *10*, e0140102. https://doi.org/10.1371/journal.pone.0140102.

### 2.59. The Effects on Neuromuscular and Physiological Parameters during Congested Period on Under-19 Futsal Players: A Pilot Study

Konstantinos Spyrou ^1,2,3,^*, Pedro E. Alcaraz ^1,3,^*, Elena Marín-Cascales ^1,3^ and Tomás T. Freitas ^1,2,3,4,5^

^1^ UCAM Research Center for High Performance Sport, UCAM Universidad Católica de Murcia, Murcia, Spain; emarin@ucam.edu (E.M.C.); tfreitas@ucam.edu (T.T.F.)

^2^ Facultad de Deporte, UCAM Universidad Católica de Murcia, Murcia, Spain

^3^ Strength and Conditioning Society, Murcia, Spain

^4^ NAR—Nucleus of High Performance in Sport, São Paulo, Brazil

^5^ Department of Human Movement Sciences, Federal University of São Paulo, Santos, Brazil

* Correspondence: kospyrou@gmail.com (K.S.); palcaraz@ucam.edu (P.E.A.)

Futsal is a high-intensity intermittent sport, in which players are exposed to high physiological and neuromuscular stress during the match, in addition to a congested schedule of games during the season [1,2]. The aim of this study was to analyze the effects of match-congestion on countermovement jump (CMJ) metrics, total recovery quality (TQR), and heart rate variability (HRV) on a sample of under-19 futsal players. Fourteen youth futsal players (age: 17.5 ± 0.5 years; body mass: 70.2 ± 8.5 kg; height: 1.80 ± 0.1 m) participated in this study. HRV, TQR questionnaire, and CMJ metrics (i.e., CMJ height, relative peak power [PP_REL_], eccentric peak force, eccentric and concentric force impulse, braking time, and time to peak force [PF_TIME_]) were registered during a congested period (i.e., three games in four days). Futsal players were separated into two groups—those who played more minutes (HIGH_MIN_) and less minutes (LOW_MIN_). A linear mixed model was used to assess the differences between groups and time-points. The group with HIGH_MIN_ only presented a significant decrease in TRQ score on Day 3 (*p* = 0.003; ES: 1.99; 95% CI: 0.55–3.15) and 4 (*p* = 0.009; ES: 1.91; 95% CI: 0.49–3.06) when compared to Day 1, and in PP_REL_ on Day 2 (*p* = 0.045; ES: 0.43; 95% CI: −0.66–1.46) and 3 (*p* = 0.030; ES: 0.42; 95% CI: 0.67–1.45) when compared to Day 1. Non-significant differences were found for the group LOW_MIN_. Considering the mean from both groups, a significant decrease (*p* ≤ 0.05; ES: 0.28–0.96) in the TQR score and PP_REL_ was found on Days 2, 3, and 4 when compared to Day 1. Lastly, a significant day*group effect was detected (*p* = 0.042) for PF_TIME_; however, non-significant and small-moderate changes were detected following the Post-Hoc comparison. In summary, neuromuscular performance (i.e., CMJ PP_REL_) and subjective recovery were impaired in players with higher playing minutes during a match-congested period when compared to those with less on-court time. In applied settings, sport practitioners should consider that athletes’ playing time is a determinant factor to consider during match-congested periods. Coaches should focus on players’ recovery, and distribute the time and competition load in short tournaments. Moreover, “alternative” variables of the CMJ, such as PP_REL_, should be assessed, when possible, as they could be more appropriate to detect changes in neuromuscular performance during congested periods.

**Funding:** This research received no external funding.

**References**

1. Oliveira, R.S.; Leicht, A.S.; Bishop, D.; Barbero-Alvarez, J.C.; Nakamura, F.Y. Seasonal changes in physical performance and heart rate variability in high level futsal players. *Int. J. Sports Med.* **2013**, *34*, 424–430. https://doi.org/10.1055/s-0032-1323720.
2. Spyrou, K.; Freitas, T.T.; Marín-Cascales, E.; Alcaraz, P.E. Physical and physiological match-play demands and player characteristics in futsal: a systematic review. *Front. Psychol.* **2020**, *11*, 2870. https://doi.org/10.3389/fpsyg.2020.569897.

### 2.60. Reduced Training Load before Competition Is Related to Improved Sleep, Fatigue and Performance in Para-Athlete Swimmers

**Eduardo Stieler ^1,2^, Marco Túlio de Mello ^1,2^, Isadora Grade ^1^, Gustavo Ramos Dalla Bernardina ^1^, Christopherson Dias Nascimento ^1^, Renato Guerreiro ^1^ and Andressa Silva ^1,2^**

^1^ Centro de Treinamento Esportivo da Universidade Federal de Minas Gerais, Belo Horizonte, MG, Brazil; eduardostieler@hotmail.com (E.S.); tmello@demello.net.br (M.T.M.); isadoragrade@hotmail.com (I.G.); gustavordalla@gmail.com (G.R.D.B.); christodias@hotmail.com (C.D.N.); guerreirorenato@hotmail.com (R.G.); andressa@demello.net.br (A.S.) 

^2^ Academia Paralímpica Brasileira, Comitê Paralímpico Brasileiro, São Paulo, SP, Brazil

The monitoring of perceptual, sleep and performance variables during a period of gradual reduction of the training load (tapering) is important to identify which variables are associated with better performance in swimming para-athletes. Therefore, the objective of the present study was to compare sleep, recovery, mood and well-being, sport performance, and reaction time (RT) of para-athlete swimmers during tapering, in addition to investigating the relationship between the variables during this training phase. Eight para-athlete swimmers were evaluated for 16 days before the main competition with an actigraphy to record sleep [1]. Assessments of well-being, recovery perception, mood (Brazilian mood scale) [2], TR (psychomotor vigilance test) [3] and performance (50 m test in the pool) were performed before the start of the training session on Mondays (Evaluation 1 = E1) and Fridays (E2) during the first and second weeks (E3 and E4), and on Tuesday (E5) of the third week, the day of travel to the competition. The training load was evaluated by the ratings of perceived exertion (RPE) [4]. The statistical analysis used was ANOVA with repeated measures, Friedman’s test, paired *t* test and Spearman’s correlation, considering a *p* ≤ 0.05. The training load gradually decreased between evaluations. The performance in the 50m test in the pool was better in E5 (37″43 ± 11″36 s) when compared to E1 (38″62 ± 12″41 s). TR was better at E5 (273 ± 65 ms) compared to E1 (313 ± 128 ms). The perception of fatigue was higher in E1 and E2 when compared to E4 and E5. Total sleep time (TST), awakenings after sleep onset (WASO) and sleep efficiency (ES) improved in the second week (TST = 480 ± 60 min; WASO = 32 ± 18 min; SE = 91 ± 3%) compared to the first (TST = 437 ± 27 min; WASO = 34 ± 16 min; ES = 90 ± 5%). Furthermore, in E5, the TST correlated with TR and performance, and the fatigue correlated with performance. It is concluded that the increase in TST and the decrease in the fatigue close to competition were correlated with the improvement of TR and performance. In addition, the tapering period with para-athlete swimmers improved performance, TR, sleep parameters and decreased the fatigue.

**Funding:** This research was funded by Conselho Nacional de Desenvolvimento Científico e Tecnológico, Fundação de Amparo à Pesquisa do Estado de Minas Gerais, Coordenação de Aperfeiçoamento de Pessoal de Nível Superior, Fundação de Apoio ao Ensino, Pesquisa e Extensão, Ministério da Cidadania, and Centro de Estudos em Psicobiologia e Exercício.

**References**

1. Samson, D.R.; Yetish, G.M.; Crittenden, A.N.; Mabulla, I.A.; Mabulla, A.Z.; Nunn, C.L. What is segmented sleep? Actigraphy field validation for daytime sleep and nighttime wake. *Sleep Health* **2016**, *2*, 342–347. https://doi.org/10.1016/j.sleh.2016.09.006.
2. Rohlfs, C.M.; Rotta, T.M.; Luft, C.D.B.; et al. Brunel Mood Scale (BRUMS): an instrument for early detection of overtraining syndrome. *RBME* **2008**, *14*, 176–181. https://doi.org/0.1590/S1517-86922008000300003.
3. Rosa, J.P.P.; Silva, A.; Rodrigues, D.F.; Simim, M.A.; Narciso, F.V.; Tufik, S.; Bichara, J.J.; Pereira, S.R.D.; Da Silva, S.C.; de Mello, M.T. Effect of bright light therapy on delayed sleep/wake cycle and reaction time of athletes participating in the Rio 2016 Olympic Games. *Chronobiol. Int.* **2018**, *8*, 1095–1103. https://doi.org/10.1080/07420528.2018.1459660.
4. Sinnott-O’Connor, C.; Comyns, T.M.; Warrington, G.D. Validity of session-rate of perceived exertion to quantify training loads in paralympic swimmers. *J. Strength Cond. Res.* **2021**, *9*, 2611–2615. https://doi.org/10.1519/JSC.0000000000003181.

### 2.61. Comparison of Resting Metabolic Rate Measured by Indirect Calorimetry and Cunninghan’s Equation in Judo Athletes during a Short Weight Loss Period

**Aline Tritto, Alexandre Dae Jin Lee, Bruno Teobaldo Campos, Danilo Magalhães Jesus, Marcos Antonio Ignácio, Victor Leopoldo, Fabiano Teixeira and Cesar Abad**

Reference Center of Sport Science of Service of Social Industry (CRCE-SESI); aline.tritto@sesisp.org.br (A.T.); alexandre.lee@sesisp.org.br (A.D.J.L.); bruno.campos@sesisp.org.br (B.T.C.); danilo.jesus@sesisp.org.br (D.M.J.); marcos.inacio@sesisp.org.br (M.A.I.); victor.leopoldo@sesisp.org.br (V.L.); fteixeira@sesisp.org.br (F.T.); cesar.abad@sesisp.org.br (C.A.) 

Judo competitions are divided into weight classes. Aiming to obtain a competitive advantage against their smaller opponents, many athletes reduce their body weight a few days prior to weigh-in. Rapid weight reductions in short periods of time require a combination of different procedures, including severe food restriction and dehydration [1]. To promote weight loss, it is well known that caloric deficit is necessary. Thus, food intake should provide fewer calories than the energy expenditure (EE). The largest component of EE is the resting metabolic rate (RMR), which accounts for 60% to 70% of total EE. It is well established that food restriction influences RMR [2], and to promote a more precise caloric deficit, measuring RMR properly could benefit judo athletes managing their body weight before competition. The methods to estimate RMR include equations such as Cunningham’s equation (CE), validated for athletes [3,4] and indirect calorimetry (the gold standard method). Concerning the importance of a precise prescription of a food plan to manage the weight loss of judo athletes before competition, the aim of this study was to compare the RMR measured by indirect calorimetry (IC) and CE. Eight professional judo athletes volunteered to participate in the study. Thirty days before the competition (−30; weight management period) and 3 days before the competition (−3; weight loss period), athletes were submitted to IC tests and anthropometric measures to calculate RMR by CE. The ANOVA two-way (method and time) was applied for mean comparisons between the RMR obtained by the CI and the CE at the two periods (−30 and −3) followed by the Bonferroni post hoc test. In addition, a comparison of the RMR delta between both methods was carried out each time. The mean RMR values found in period −30 were 2148 ± 406 kcal for CI and 1746 ± 194 kcal for CE (*p* = 0.02). In the −3 period, the mean value for CI was 1823 ± 309 kcal against 1717 ± 179 kcal for CE (*p* = 0.03). The deltas were −326 + 178 kcal for CI and −29 + 18 kcal for CE. Student’s *t* test showed a significant difference (*p* < 0.01) between the methods, suggesting the inaccuracy of CE to predict RMR of judo athletes during a rapid weight loss period. In conclusion, CE does not seem to be a sensitive method to estimate RMR. In contrast, IC is recommended because it enables accurate information for individualized nutrition planning for weight loss in judo athletes during a short weight loss period.

**Funding:** This research received no external funding.

**References**

1. Artioli, G.G.; Gualano, B.; Franchini, E.; Scagliusi, F.B.; Takesian, M.; Fuchs, M.; Lancha Jr, A.H. Prevalence, magnitude, and methods of rapid weight loss among judo competitors. *Med. Sci. Sports Exerc.* **2010**, *42*, 436–442. https://doi.org/10.1249/MSS.0b013e3181ba8055.
2. Martin, C.K.; Heilbronn, L.K.; de Jonge, L.; DeLany, J.P.; Volaufova, J.; Anton, S.D.; Redman, L.M.; Smith, S.R.; Ravussin, E. Effect of calorie restriction on resting metabolic rate and spontaneous physical activity. *Obesity* **2007**, *15*, 2964–2973. https://doi.org/10.1038/oby.2007.354.
3. Cunningham, J.J. Body composition as a determinant of energy expenditure: a synthetic review and a proposed general prediction equation. *Am. J. Clin. Nutr.* **1991**, *54*, 963–969. https://doi.org/10.1093/ajcn/54.6.963.
4. Freire, R.; Pereira, G.; Alcantara, J. M.; Santos, R.; Hausen, M.; Itaborahy, A. New Predictive Resting Metabolic Rate Equations for High-Level Athletes: A Cross-validation Study. *Med. Sci. Sports Exerc.* 2022, *Epub ahead of print*. https://doi.org/10.1249/MSS.0000000000002851.

### 2.62. Effects of 1 Week of Training Cessation after the End of the Competitive Season on Physical Performance of Youth Basketball Players

Christian H. Van der Berg ^1^, Antonio Martínez-Serrano ^1,2^, Elena Marín-Cascales ^1,2^, Konstantinos Spyrou ^1,2^, Pedro E. Alcaraz ^1,2^ and Tomás T. Freitas ^1,2,3,4,5,^*

^1^ UCAM Research Center for High Performance Sport, UCAM Universidad Católica de Murcia, Murcia, Spain; chberg@live.no (C.H.V.B.); amartinez30@ucam.edu (A.M.S.); emarin@ucam.edu (E.M.C.); kspyrou@ucam.edu (K.S.); palcaraz@ucam.edu (P.E.A.)

^2^ Strength and Conditioning Society, Murcia, Spain

^3^ Facultad de Deporte, UCAM Universidad Católica de Murcia, Murcia, Spain 

^4^ NAR—Nucleus of High Performance in Sport, São Paulo, Brazil

^5^ Department of Human Movement Sciences, Federal University of São Paulo, Santos, Brazil

* Correspondence: tfreitas@ucam.edu (T.T.F.) 

Long-term detraining periods may lead to reduced sports performance [1]. However, evidence is conflicting when it comes to the effects of short-term detraining or training cessation [2,3]. Moreover, the literature is scarce regarding specific neuromuscular adaptations to these periods in youth athletes. The aim of this study was to analyze the effects of 1 week of training cessation on youth basketball players. Forty-three athletes, male (n = 24, age 14.5 ± 1.3, years, height 182.1 ± 9.5 cm, weight 69 ± 11.8 kg) and female (n = 19, age 16.3 ± 1.3 years, height 171.2 ± 6.8 cm, weight 62.2 ± 7.5 kg), of three different age groups (Under-14 [U14], U16, and U18) underwent a test battery before and after a training cessation period of 1 week, immediately following the end of the competitive season. Countermovement jump (CMJ), horizontal jump (HJ), 10 m sprint and L-drill change of direction (COD) tests were completed. To analyze the pre–post changes among the different groups and time-points, a repeated-measure ANOVA with post-hoc was conducted. Results showed that HJ performance increased significantly (*p* = 0.003) following the training cessation period. In the 10 m sprint, a time*group interaction was observed with the U16 displaying significant declines in performance after training cessation when compared to the U14 and U18 (*p* = 0.50). The L-drill test improved significantly (*p* = 0.022) but no time*group interaction was found. Regarding CMJ performance, no changes were observed following training cessation in jump height and concentric peak power in any of the groups. Although a significant time*group effect was found for eccentric peak power, no significant differences were observed following post-hoc analysis. Reactive strength index modified (RSImod), significantly improved in the U14 (*p* = 0.018) and declined in the U18 group (*p* = 0.046). Finally, regarding braking time, a significant time*group interaction was observed as the U14 group displayed greater improvements (*p* = 0.017) than the U16 and U18. In summary, a 1-week training cessation resulted in improvements in HJ and COD performance in all age groups. Furthermore, it appears that the U14 group benefited the most from the training cessation period, as seen by the greater improvements in selected CMJ variables (i.e., RSImod and braking time) and the faster sprint times compared to the other groups. Practitioners should consider that training cessation periods should be adjusted according to the athletes’ age and that, in U14 players (i.e., circa-peak height velocity), 1 week of training cessation at the end of the competitive season seems to be beneficial for neuromuscular performance.

**Funding:** This research received no external funding.

**References**

1. Mujika, I.; Padilla, S. Detraining: loss of training-induced physiological and performance adaptations. Part II: Long term insufficient training stimulus. *Sports Med.* **2000,** *30*, 145–154. https://doi.org/10.2165/00007256-200030030-00001.
2. Mujika, I.; Padilla, S. Detraining: loss of training-induced physiological and performance adaptations. Part I: short term insufficient training stimulus. *Sports Med.* **2000,** *30,* 79–87. https://doi.org/10.2165/00007256-200030020-00002.
3. Pereira, L.A.; Freitas, T.T.; Pivetti, B.; Alcaraz, P.E.; Jeffreys, I.; Loturco, I. Short-term detraining does not impair strength, speed, and power performance in elite young soccer players. *Sports* **2020**, *8*, 141. https://doi.org/10.3390/sports8110141.

### 2.63. The Validity and Reliability of Jumpo2 and MyJump2 for Estimating Vertical Jump Variables

Amilton Vieira ^1,^*, Gabriela Ribeiro ^1^, Victor Macedo ^1^, Valdinar Rocha-Junior ^1^, Roberto Baptista ^1,2^, Carlos Gonçalves ^1^, Rafael Cunha ^1^ and James Tufano ^3^

^1^ Strength and Conditioning Laboratory, Faculty of Physical Education, University of Brasilia (UnB), Brasilia, DF, Brazil; gabriela-lima.gl@aluno.unb.br (G.R.); victor.macedo10@hotmail.com (V.M.); valdinar@gmail.com (V.R.J.); robertobaptista@unb.br (R.B.); carlos.goncalves@aluno.unb.br (C.G.); rafaelcunha05@gmail.com (R.C.) 

^2^ Automation and Robotics Laboratory, Faculty of Technology, University of Brasilia (UnB), Brasilia, DF, Brazil

^3^ Faculty of Physical Education and Sport, Charles University in Prague, Prague, Czech Republic; james.j.tufano@gmail.com (J.T.) 

* Correspondence: amiltonvieira@unb.br (A.V.)

Vertical jump testing is widely used to monitor neuromuscular performance. Smartphone applications (App) have been developed to measure jumping performance as alternatives to expensive devices, such as force plates or contact mats. One of the most popular Apps, MyJump, which operates on the iOS system, has been shown to provide valid and reliable measures of the height of countermovement (CMJ) and squat jumps (SJ) (estimated from flight time) [1]. More recently, another study found valid and reliable measures of both CMJ and SJ flight time when using the JumPo App, which runs on an Android operating system, allowing for a far broader user base than Apps on iOS [2]. As these Apps directly measure flight time, it is logical that these data can be used to estimate jump height. However, these Apps take things a step further and claim to calculate the mean values of force, velocity, and power produced during the propulsive phase (i.e., the ascendant aspect of the movement) of the vertical jump [3]. We investigated the concurrent validity and test–retest reliability of the JumPo 2 and MyJump 2 Apps for estimating jump height and the mean values of force, velocity, and power produced during CMJ and SJ. Physically active university-aged men (n = 10, 20 ± 3 years, 176 ± 6 cm, 68 ± 9 kg) jumped on a force plate (i.e., criterion) while being recorded by a smartphone slow-motion camera. The videos were analyzed using JumPo 2 and MyJump 2 using a Samsung Galaxy S7 powered by Android System. Validity and reliability were determined by regression analysis, typical error of estimates and measurements, and intraclass correlation coefficients. Both Apps provided a reliable estimate of jump height and the mean values of force, velocity, and power. Furthermore, estimates of jump height for CMJ and SJ and the mean force of the CMJ were valid. However, the Apps presented impractical or poor validity correlations for velocity and power. Compared with the criterion, the Apps underestimated the velocity of the CMJ. Therefore, JumPo 2 and MyJump 2 both provide a valid measure of jump height, but the remaining variables provided by these Apps must be viewed with caution since the validity of force depends on jump type, while velocity (and as consequence power) could not be well estimated from the Apps.

**Funding:** This research received no external funding.

**References**

1. Balsalobre-Fernández, C.; Glaister, M.; Lockey, R.A. The Validity and Reliability of an IPhone App for Measuring Vertical Jump Performance. *J. Sports Sci.* **2015**, *33*, 1574–1579. https://doi.org/10.1080/02640414.2014.996184.
2. Vieira, A.; Blazevich, A.J.; DA Costa, A.S.; Tufano, J.J.; Bottaro, M. Validity and Test-retest Reliability of the Jumpo App for Jump Performance Measurement. *Int. J. Exerc. Sci.* **2021**, *14*, 677–686.
3. Samozino, P.; Morin, J.B.; Hintzy, F.; Belli, A. A Simple Method for Measuring Force, Velocity and Power Output during Squat Jump. *J. Biomech.* **2008**, *41*, 2940–2945. https://doi.org/10.1016/j.jbiomech.2008.07.028.

### 2.64. Effects of Exercise Volume in Aerobic Fitness, Body Weight and Antioxidant Capacity Promoted by HIIT

Michele Xavier ^1,2^, Diego Gomes ^1,^* and Veronica Salerno ^1^

^1^ Universidade Federal do Rio de Janeiro; michelefreitasxavier@hotmail.com (M.X.); vpsalerno@gmail.com (V.S.)

^2^ Marinha do Brasil

* Correspondence: diegoefd@gmail.com (D.G.)

Regular exercise improves and maintains physical fitness, body weight and antioxidant capacity [1]. However, many people commonly cite “lack of time” as the greatest barrier to adopting and maintaining a more physically active lifestyle [2]. To achieve exercise benefits with the shortest time commitment, high-intensity interval training (HIIT) programs have been developed. Here, we assessed the impact of two different frequencies of performing the same, low-volume HIIT program on weight loss, physical performance, and oxidative stress in Wistar rats. Animals were divided into three groups: sedentary (control; n = 7), trained three times a week (3×; n = 7), and trained seven days a week (7×; n = 7) for 2 months. Training was a HIIT protocol consisting of fourteen, twenty-second bouts of swimming with an additional load of up to 12% of their body weight. There was a ten-second recovery interval between repetitions. Body weight was measured before and after the study period while physical performance was measured in a maximal swimming test. Oxidative stress markers and antioxidants were measured in plasma or erythrocytes. At the end of the study, body weight was lower in the 7× group compared with the sedentary and 3× group. The 7× group also displayed a significant increase in maximum swimming time compared to the control and 3× groups. For oxidative stress markers, the group that trained every day (7×) showed lower protein carbonylation levels compared to the control group and less lipid peroxidation compared to the low frequency (3×) group. The enzymatic antioxidant activity of catalase was significantly increased in erythrocytes from animals in the seven days a week group (7×) compared to the three times a week (3×) group. Overall, the improvement in physical performance was directly proportional to exercise volume while weight gain was inversely proportional. Training volume was also directly related to decreased oxidative stress, increased antioxidant capacity and body weight control.

**Funding:** This research received no external funding.

**References**

1. Shen, Y.; Xu, X.; Yue, K.; Xu, G. Effect of different exercise protocols on metabolic profiles and fatty acid metabolism in skeletal muscle in high-fat diet-fed rats. *Obesity (Silver Spring)* **2015**, *23*, 1000–1006. https://doi.org/10.1002/oby.21056.
2. Bauman, A.E.; Reis, R.S.; Sallis, J.F.; Wells, J.C.; Loos, R.J.F.; Martin, B.W. Correlates of physical activity: why are some people physically active and others not? *Lancet* **2012**, *380*, 258–271. https://doi.org/10.1016/S0140-6736(12)60735-1.

